# Strain features and distributions in pneumococci from children with invasive disease before and after 13-valent conjugate vaccine implementation in the USA

**DOI:** 10.1016/j.cmi.2015.08.027

**Published:** 2016-01

**Authors:** B.J. Metcalf, R.E. Gertz, R.A. Gladstone, H. Walker, L.K. Sherwood, D. Jackson, Z. Li, C. Law, P.A. Hawkins, S. Chochua, M. Sheth, N. Rayamajhi, S.D. Bentley, L. Kim, C.G. Whitney, L. McGee, B. Beall, Active Bacterial Core surveillance team

**Affiliations:** 1) Centers for Disease Control and Prevention, National Center for Immunization and Respiratory Diseases, Atlanta, GA, USA; 2) Wellcome Trust Sanger Institute, Hinxton, UK; 3) Department of Medicine, Addenbrookes Hospital, University of Cambridge, UK; 4) Centers for Disease Control and Prevention, National Center for Emerging and Zoonotic Infectious Diseases, Atlanta, GA, USA

**Keywords:** Antimicrobial susceptibility, clonal complexes, pneumococcal conjugate vaccines, serotype distributions, whole genome sequence

## Abstract

The effect of second-generation pneumococcal conjugate vaccines on invasive pneumococcal disease (IPD) strain distributions have not yet been well described. We analysed IPD isolates recovered from children aged <5 years through Active Bacterial Core surveillance before (2008–2009; *n* = 828) and after (2011–2013; *n* = 600) 13-valent pneumococcal conjugate vaccine (PCV13) implementation. We employed conventional testing, PCR/electrospray ionization mass spectrometry and whole genome sequence (WGS) analysis to identify serotypes, resistance features, genotypes, and pilus types. PCV13, licensed in February 2010, effectively targeted all major 19A and 7F genotypes, and decreased antimicrobial resistance, primarily owing to removal of the 19A/ST320 complex. The strain complex contributing most to the remaining β-lactam resistance during 2011–2013 was 35B/ST558. Significant emergence of non-vaccine clonal complexes was not evident. Because of the removal of vaccine serotype strains, positivity for one or both pilus types (PI-1 and PI-2) decreased in the post-PCV13 years 2011–2013 relative to 2008–2009 (decreases of 32–55% for PI-1, and >95% for PI-2 and combined PI-1 + PI-2). β-Lactam susceptibility phenotypes correlated consistently with transpeptidase region sequence combinations of the three major penicillin-binding proteins (PBPs) determined through WGS analysis. Other major resistance features were predictable by DNA signatures from WGS analysis. Multilocus sequence data combined with PBP combinations identified progeny, serotype donors and recipient strains in serotype switch events. PCV13 decreased the frequency of all PCV13 serotype clones and concurrently decreased the frequency of strain subsets with resistance and/or adherence features conducive to successful carriage. Our results serve as a reference describing key features of current paediatric IPD strains in the USA after PCV13 implementation.

## Introduction

*Streptococcus pneumoniae* can quickly adapt to selective pressures through horizontal transfer and intrachromosomal mutation events. Even so, the conjugate vaccine strategy of targeting currently predominant invasive serotypes has been highly successful [1], [2] and durable [3] in decreasing the frequency of invasive pneumococcal disease (IPD) and pneumococcal antimicrobial resistance. However, individual variants that originated through serotype-switching events substantially contributed to disease burdens in the USA in the post-seven-valent pneumococcal conjugate vaccine (PCV7) era [4], [5], [6], [7], [8]. Although the monitoring of pneumococcal serotypes and antimicrobial susceptibilities provides the major information for devising prevention strategies, approaches that deduce the causal chromosomal changes that lead to the emergence of individual invasive strains allow for a better biological and epidemiological understanding of pneumococcal strains. Here, we have provided a strain reference that supplies distributions and genetic features of each invasive serotype that we detected in a US surveillance system during a key 5-year period of IPD surveillance.

## Materials and methods

### Patients

Active Bacterial Core surveillance (ABCs) is an active, population-based and laboratory-based surveillance system that is part of the CDC Emerging Infections Program. Cases of IPD were defined by the isolation of pneumococci from a normally sterile site in residents of the surveillance areas in ten different states [1], [2], [3] (see ABCs surveillance reports for population sizes and IPD incidence at http://www.cdc.gov/abcs/reports-findings/surv-reports.html). Throughout the entire 2005–2013 period, the number of individuals aged <5 years in the ABCs catchment area remained approximately 2.1 million.

### Isolates

For the years of the primary focus (2008–2009 and 2011–2013), isolates corresponding to 84.7–90.4% of reported cases were available to the CDC *Streptococcus* Laboratory for characterization (average, 87.9%). For each of these 5 years, all of these isolates were characterized with respect to serotypes, multilocus sequence type (MLST)-based clonal complexes (CCs), resistance features, and pilin subunit presence or absence.

**Fig. S1:**
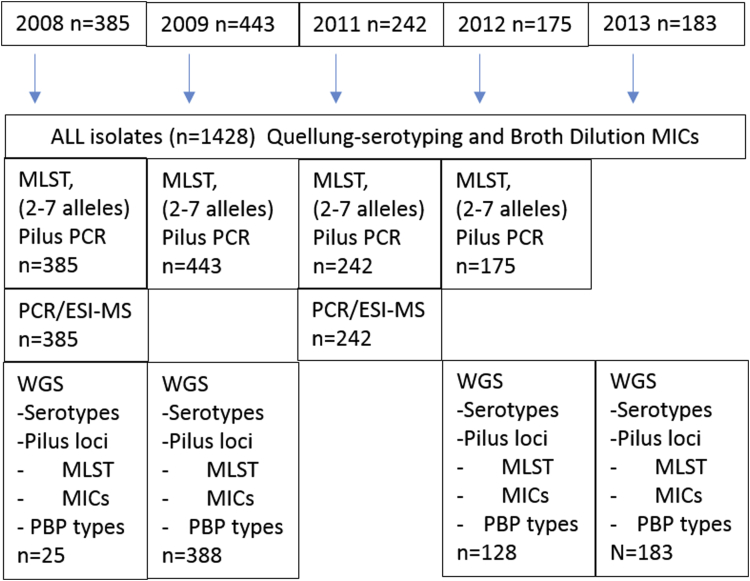
Flow diagram description of the testing performed on 1428 year 2008–2009 and 2011–2013 ABCs isolates.

Isolates were characterized by use of a combination of conventional testing, PCR/electrospray ionization mass spectrometry (ESI-MS), and short-read whole genome sequence (WGS) analysis. In total, 1428 isolates, corresponding to 87.9% of the 1625 documented IPD cases in children aged <5 years, were available for characterization (828/948 for 2008–2009, and 600/677 for 2011–2013) (Supplementary Fig. S1).

### Capsular serotyping

All isolates from 2005 through 2013 were serotyped with latex agglutination and the Quellung reaction employing CDC antisera.

### WGS analysis

Of the 801 combined year 2009, 2012 and 2013 IPD isolates described in this study, serotypes, antimicrobial susceptibilities, MLSTs and pilus types for 699 (87.3%) were determined by the use of our pneumococcal typing pipeline with Illumina WGS fastq files provided by the Sanger group (*n* = 516 from year 2009 and 2012 IPD isolates) and the CDC Biotechnology Core Facility (*n* = 183 from year 2013 IPD isolates). The WGS-derived data from these 699 isolates recovered during 2009, 2012 and 2013 were used to supplement the already accumulated conventional serotyping, antimicrobial susceptibility testing, MLST, pilus locus PCR and PCR/ESI-MS data (described below). WGS-based identification of serotypes, pilus loci and non-β-lactam antimicrobial resistance features employed query DNA sequences described in Table S1 (serotypes and pili) and Table S2 (non-β-lactam antimicrobial resistance). Transpeptidase domain amino acid sequences of 277–359 residues from penicillin-binding proteins (PBPs) 1a, 2b, and 2x were extracted from approximately 1600 ABCs strains characterized over the years 1998–2013. From this, databases of 69, 77 and 127 unique transpeptidase domain amino acid sequences were compiled for PBP1a, PBP2b, and PBP2x, respectively (Tables S3–S5). Each unique sequence was assigned an identifier (sequences 1a-0 to 1a-68 for PBP1a, sequences 2b-0 to 2b-78 for PBP2b, and sequences 2x-0 to 126 for PBP2x). The three-number combination from each isolate was correlated with MICs for each of the six different β-lactam antibiotics. For example, the basally β-lactam-sensitive TIGR4 strain (genbank accession AE005672) contains a composite amino acid sequence pattern of 1a-0, 2b-0, and 2x-0 (abbreviated as 0:0:0). The corresponding DNA sequences (e.g. *1a-0*, *2b-0*, and *2x-0*) are italicized. Full-length PBP genes are referred to with standard nomenclature (*pbp1a*, *pbp2b*, and *pbp2x*).

Detection of non-core genome-conferred resistance loci was performed according to homology with known determinants. Core genome-encoded resistance determinants (fluoroquinolones, co-trimoxazole, ribosomal protein mutation-conferred macrolide/lincosamide/streptogramin resistance, and rifampin) were screened by identifying specific amino acid substitutions with previously described targets (Table S2). Table S2 describes the sequence coordinates used for resistance queries. The bioinformatics methods used are described in Doc. S1.

### Serotype verification, CC determination and pilus determinant detection for year 2008 and 2011 isolates

Quellung-determined serotypes were verified from IPD isolates (*n* = 622) from 2008 and 2011 by use of the previously described PCR/ESI-MS) [9]. CCs were assigned on the basis of known clone and serotype associations with partial to complete MLST profiles (obtained by Sanger sequencing) and antimicrobial resistance profiles as previously described [8], [10]; these CCs were then verified with PCR/ESI-MS. Year 2009 isolates, most of which were also subjected to WGS analysis as described above, were processed in the same manner, except without PCR/ESI-MS. Pilus determinant PCR-based detection was performed with previously described primers [11]. Twenty-five of the year 2008 isolates were also retrospectively subjected to the WGS typing pipeline. Serotypes, pilus determinants and MLSTs obtained with WGS were in complete concordance with the results obtained with the other methods.

### Assignment of CC reference MLSTs

Multilocus sequence typing relied upon the database at http://pubmlst.org/spneumoniae/. Forty-seven new sequence types (STs) and 21 new MLST alleles from this study were deposited in this database. CCs were defined as sharing four or more alleles with a given reference ST. CC reference STs were chosen according to abundance within a given serotype and/or their presence within multiple serotypes of the study, and differed by at least three alleles from other CC reference STs.

### Antimicrobial susceptibility

MICs were determined with the microbroth dilution method as previously described [8]. The antimicrobials tested included penicillin, amoxycillin, meropenem, cefotaxime, ceftriaxone, cefuroxime, erythromycin, clindamycin, co-trimoxazole, tetracycline, chloramphenicol, rifampin, levofloxacin, synercid, linezolid, telithromycin, and vancomycin. MICs were interpreted according to 2013 CLSI guidelines [12], with exceptions for the different β-lactam antibiotics and chloramphenicol. Penicillin susceptibility, intermediate resistance, resistance and high-level resistance were defined as (in mg/L) ≤0.06, 0.12–1.0, 2.0, and ≥4, respectively. The highest MIC observed for cefotaxime and/or ceftriaxone was used as a single MIC value for third-generation cephalosporins (these two antibiotics usually showed the same MIC, and never varied by more than a single dilution), with susceptibility, intermediate resistance, resistance and high-level resistance defined as ≤0.5, 1.0, 2.0, and ≥4, respectively. For amoxycillin, susceptibility, intermediate resistance, resistance and high-level resistance were defined as ≤0.12, 0.25–1, 2.0, and ≥4, respectively. For meropenem, susceptibility, intermediate resistance, resistance and high-level resistance were defined at ≤0.25, 0.5, 1.0, and ≥2.0, respectively. Only chloramphenicol MICs of >8 mg/L were considered to indicate resistance, differing from the CLSI value of ≥8 mg/L. Intermediate and full co-trimoxazole (trimethoprim–suphamethoxazole) resistance was defined as MICs (for trimethoprim–sulphamethoxazole) of 1/19–2/38 mg/L and ≥4/76 mg/L, respectively.

### Fastq file accession

Accession information and sequencing methodology details for the 727 fastq files utilized in this work are provided in Table S6.

## Results

### IPD due to PCV13-targeted serotypes

**Fig. 1:**
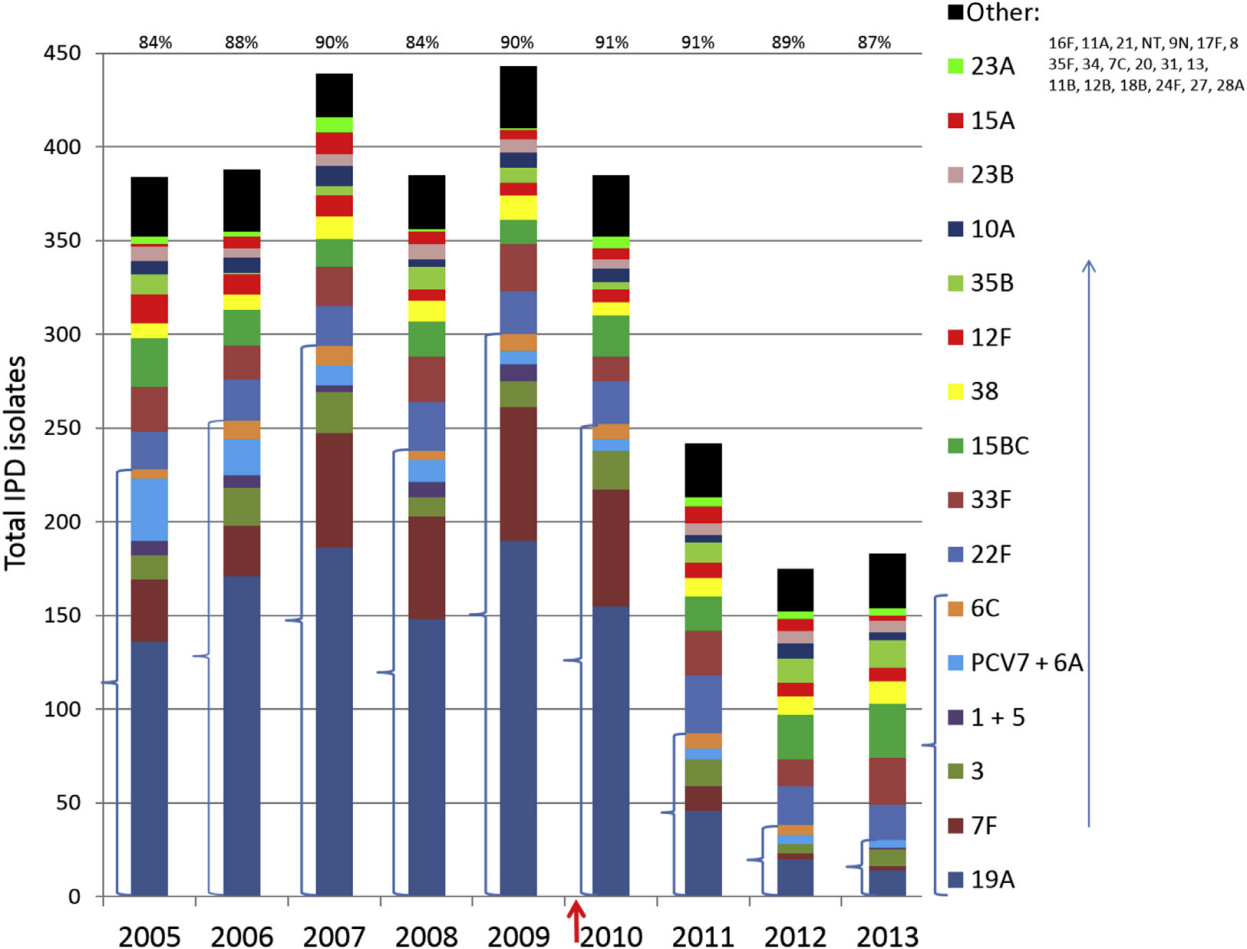
Invasive pneumococcal disease (IPD) isolate serotype distributions in children aged <5 years during 2005–2013 as identified through Active Bacterial Core surveillance. Thirteen-valent pneumococcal conjugate vaccine (PCV13) serotypes and 6C are bracketed. The arrow indicates PCV13 implementation in February, 2010. The percentages shown above each year indicate the ratios of IPD isolates to documented IPD cases (see http://www.cdc.gov/abcs/reports-findings/surv-reports.html). NT, non-typeable; PCV7, seven-valent pneumococcal conjugate vaccine.

A total of 3024 isolates were collected through ABCs during 2005–2013 from 3443 cases of IPD in children aged <5 years. The percentage of IPD cases with corresponding isolates ranged from 84% to 91% (Fig. 1). During 2005–2009, overall rates of IPD in children aged <5 years of age remained at 21–23 cases per 100 000 individuals [2], as reflected by 384–443 IPD isolates for each of these years within the surveillance population (Fig. 1). The yearly number of 13-valent pneumococcal conjugate vaccine (PCV13) serotype isolates ranged from 223 to 290, accounting for 58–65% of the total IPD isolates. As recently reported [2], the major causes of IPD in children aged <5 years immediately prior to PCV13 implementation (early 2010) were PCV13 serotypes 19A and 7F. During 2005–2009, the numbers of serotype 19A and 7F isolates for each year were 136–190 and 27–71, respectively, with the largest numbers of isolates for both serotypes being recovered during 2009. The incidence of these serotypes dropped dramatically during 2011–2013, with only 14 serotype 19A isolates and two serotype 7F isolates being recovered during 2013. PCV7 serotypes and serotype 6A cumulatively accounted for only 76 isolates during 2005–2009 (3.7%) and for 15 isolates during 2011–2013 (2.5%). Serotype 19F was the most common PCV7-targeted serotype encountered, with 33 isolates (43.4%) being recovered during 2005–2009 and nine isolates (60%) being recovered during 2011–2013. Throughout the 9-year period, there was a general trend for a decrease in the number of PCV7-targeted serotypes, from a high of 28 isolates recovered during 2005 to a low of four isolates recovered during 2013. There were no isolates recovered of the four PCV7-targeted serotypes 6A, 6B, 9V and 23F during 2011–2013, as compared with 23 recovered during 2005–2009.

Whereas there were a small number of IPD cases due to PCV13 serotypes 1 and 5 during 2005–2009 (29 serotype 1 and five serotype 5 isolates recovered), there were no cases detected during 2010–2012, and there was only one serotype 1 isolate recovered during 2013. The minor paediatric IPD serotype 6C is also predicted to be targeted by PCV13 through cross-protection from the 6A antigen [13], which adds a small increment to the total of potential PCV13-preventable cases (5–12 cases during each of the years 2005–2012; total of 64 isolates). There was no reported serotype 6C paediatric IPD during 2013; however, paediatric serotype 6C IPD in prior years was too rare for the significance of this finding to be calculated.

### IPD due to non-PCV13 serotypes

The four most frequent causes of IPD during 2011–2013 were serotypes 33F, 22F, 15B/15C, and 35B; there were cumulatively 244 isolates recovered of these four serotypes during these three years (72–88 isolates per year). Serotypes 38, 12F, 23B, 10A, 15A and 23A combined accounted for 120 isolates during these three years (36–42 each year). Each of these non-vaccine serotypes showed relatively little fluctuation in IPD incidence throughout the entire period (2005–2013), and there were no clear indications of so-called serotype replacement disease. Although the number of 33F IPD isolates nearly doubled in 2013 (*n* = 25) relative to 2012 (*n* = 14), the numbers of serotype 33F IPD isolates recovered were nearly identical during each of the years 2005, 2008, 2009, 2011, and 2013 (Fig. 1). The remaining, less frequently encountered, non-PCV13 serotypes that were encountered during the 2005–2013 period, listed according to frequency (cumulative isolates), were 16F (38), 11A (37), 21 (29), 8 (19), 9N (19), 17F (18), 35F (17), 34 (14), 7C (12), 20 (8), 31 (8), 13 (3), 11B (1), 12B (1), 18B (1), 24F (1), 27 (1), and 28A (1). These serotypes also showed no clear indications of becoming increasingly common after PCV13 introduction, and cumulatively accounted for only 18–31 isolates each year during 2005–2013 (18–28 during 2011–2013). There were 24 total non-typeable (NT) IPD isolates recovered during 2005–2013. Features of the nine NT isolates recovered during 2008–2009 and 2011–2013 are described below.

### Serotype and genotype diversity of invasive pneumococci recovered during 2008–2013

**Fig. 2:**
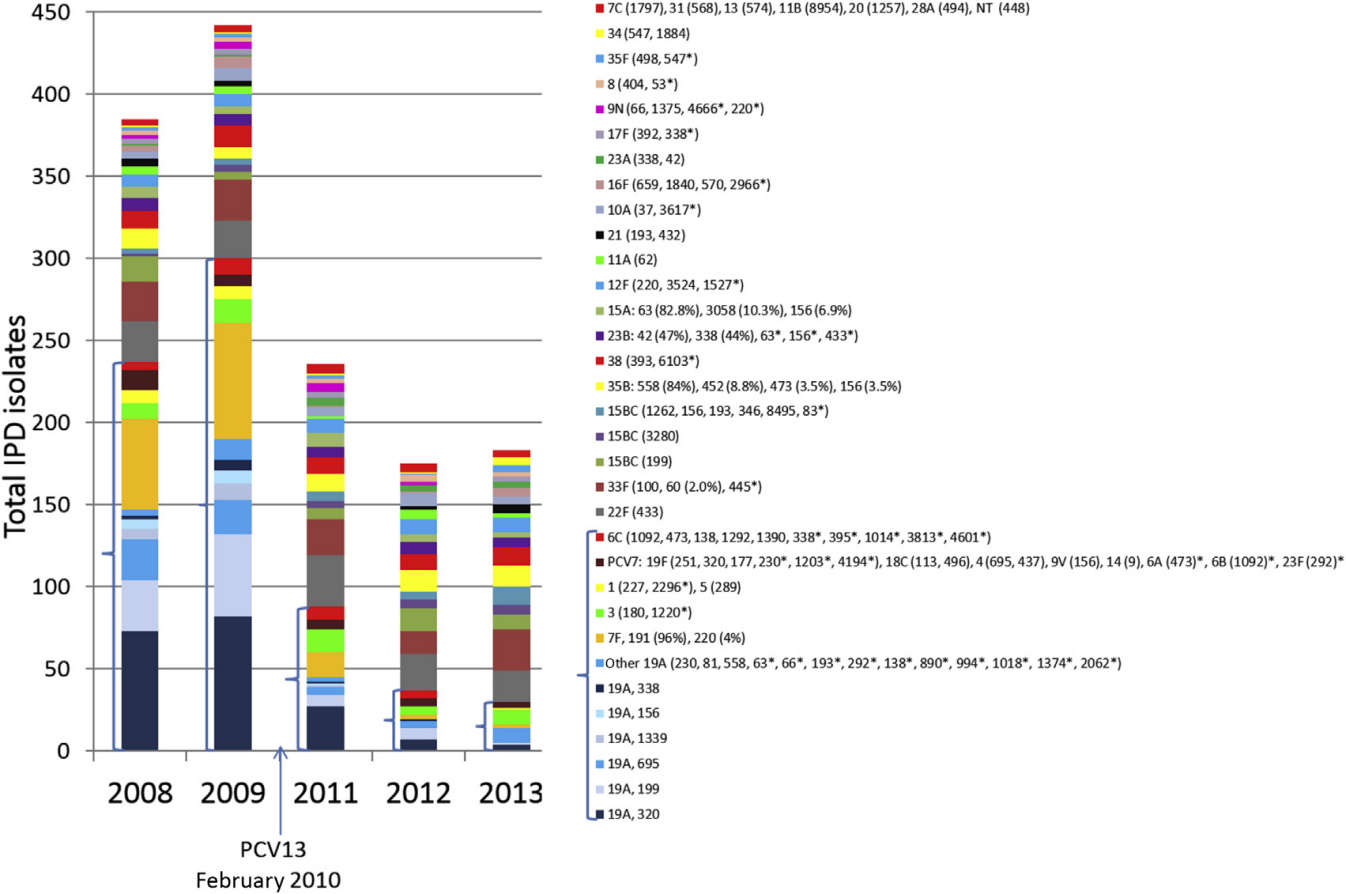
Comprehensive listing of clonal complex (CC) associations with the serotypes encountered in invasive pneumococcal disease (IPD) isolates during 2008–2009 and 2011–2013. Asterisks indicate CCs that were encountered in single isolates. Thirteen-valent pneumococcal conjugate vaccine (PCV13) serotypes and 6C are bracketed. Certain variants are presented as percentages; for example 7F (191) constitutes 96% of serotype 7F, and 7F (220) constitutes 4%. NT, non-typeable.

A total of 1428 IPD isolates were characterized from the years 2008–2009 and 2011–2013. Fig. 2 is provided to give a comprehensive representation of the CC diversity within each serotype during these 5 years. This work included WGS-based characterization of 699 isolates recovered during 2009, 2012, and 2013 (Table 1), representing 35 of the 39 serotypes that, together with nine NT isolates, accounted for all IPD cases during the 5 years of the study (serotype 6A, 6B, 14 and 20 isolates were not recovered during 2009, 2012, and 2013). The WGS data included WGS-derived capsular serotypes, MLSTs, transpeptidase protein sequence type patterns, pilus determinant detection, and sequence-based antimicrobial resistance markers (correlated with conventional MIC-based results).

### Genetic composition of individual non-vaccine type (NVT) serotypes

Below, we provide selected information regarding specific NVT serotypes of interest from among the 27 total NVTs encountered. We have restricted this text to predominant serotypes and individual variants that potentially reflect past serotype switch events or express unusual resistance properties within given serotypes.

### Serotypes 22F and 33F

Serotypes 22F and 33F were each composed primarily of single MLST-based complexes that appear to have been largely unchanged throughout 2008–2013 and in comparison with the year immediately before PCV7 introduction (1999) [14]. Both serotypes uniformly lacked pilus determinants PI-1 and PI-2 (Table 1). As also shown in Table 1, approximately 25% of serotype 22F isolates recovered during 2009, 2012 and 2013 expressed *mef*-associated erythromycin resistance. Most serotype 33F isolates were resistant to erythromycin (*mef-*positive) and were primarily intermediately resistant to co-trimoxazole. Intermediate co-trimoxazole resistance in these and most other isolates of this phenotype, was associated with one or two codon insertions between bases 168 and 201 of the *folP* structural gene. A small number of serotype 33F isolates were fully co-trimoxazole resistant, and this was was associated with both *folP* insertion and the *folA* I100L substitution. The single β-lactam-non-susceptible (NS) serotype 33F strain (phenotype IISSR) featured a PBP profile (4:23:7) suggestive of previous horizontal transfer(s) of PBP gene sequences from a CC558 donor strain into a common 33F/CC100 strain, as 1a-4 and 2x-7 are found nearly exclusively in β-lactam-NS 35B/CC558 strains (Table 1). Although 2b-23 is unique in our database to this specific serotype 33F isolate, it also appears to have originated from a recombination event with a β-lactam-NS strain, as it contains six substitutions that closely neighbour the active site serine SXXK and SXN. These include three of four substitutions in this region (T426K, Q427L, and T446A) that are associated with intermediate penicillin resistance [15].

### Serotypes 15B and 15C

Serotypes 15B and 15C show nearly identical CC distributions (data not shown), owing to their frequent interconversion based on variation within *wciZ15B* [16]. Of the 185 combined serotype 15B and 15C isolates recovered during 2005–2013, 115 (62%) were serotype 15C. The ability to resolve these two serotypes was included within our typing pipeline, owing to the reported low cross-reactivity of functional antibodies against serotype 15B with serotype 15C [17], which is a consideration in the formulation of new vaccines. These two serotypes accounted for 84 isolates for the years for which we provided genotyping (2008–2009 and 2011–2013), and showed a high level of genetic heterogeneity as compared with the pre-PCV7 years, when ABCs serotype 15B/15C isolates were primarily comprised of CC199 [14]. Although CC199 was the single most common serotype 15B/15C CC, numerous additional CCs as a group constituted up to 65% of these two serotypes (Fig. 2), with CC3280 being the most frequent secondary CC. As shown in Table 1, CC3280 has markedly more resistance features than the other common serotype 15B/C CCs (resistance to macrolides and co-trimoxazole; low-level β-lactam resistance). Several other CCs were evident at low frequencies, including CC156. CC156, which is characteristic of the major serotype 9V strain that flourished prior to PCV7 implementation [14], featured a number of single-locus variants within serotypes 15A, 15B/15C and 23B that, unlike their serotype 9V counterparts, were uniformly susceptible to β-lactams and generally susceptible to other antibiotics (Table 1).

A single highly resistant serotype 15C strain was a single-locus variant (ST83) of ST81, which is the genotype of the highly successful multiresistant PMEN-1 [18]. This strain showed moderate to high resistance to all five β-lactam classes, and was resistant to the five additional antimicrobials listed in Table 1. This ST83 strain shared PBP profile 15:12:18, which was also found within a multiresistant serotype 19A/ST2346 strain (ST81 single-locus variant) and two serotype 9V/ST156 strains in this study (Table 1). The 15:12:18 profile is also shared among numerous pre-PCV7 IPD isolates of serotypes 9V, 19F and 23F within CC81 and CC156 in our master pneumococcal genome database (data not shown). Prior to this study, ST83 was associated in the global MLST database solely with strains collected in Asian countries during 1995–2013 that primarily included serotype 23F strains, but also included serotype 15B and 15C strains.

### Serotype 35B

Serotype 35B was primarily comprised of the serotype 35B ST558 complex during all 5 years, which slightly increased in frequency during 2011–2013 (Fig. 2). This strain complex is typically associated with the presence of pilus PI-1 and elevated MICs for penicillin and other β-lactam antibiotics (Table 1; Fig. 4, Fig. 5). It is interesting that, during the pre-PCV7 years, serotype 35B strains rarely caused IPD in children, and the now uncommon β-lactam-sensitive serotype 35B complex (CC452, one isolate depicted in Table 1) accounted for approximately 31% of 35B IPD isolates from patients of all ages [19]. Two additional interesting CCs were observed within serotype 35B. A single-locus serotype 35B variant of ST473 (ST3822; also described in Table 1) was recovered during 2009. CC473 has been previously associated with serotypes 6A and 6C [10], and ST3822 has been recorded from a single serotype 6A blood isolate recovered during 2008 in Ireland (http://pubmlst.org/spneumoniae/). Because of PBP allele and MLST associations, the two serotype 35B/CC156 isolates (Table 1) appear to be progeny of serotype switch events, and are described in more detail in the section below pertaining to capsular locus switching.

### Serotype 6C

The most genetically heterogeneous NVT serotypes were serotypes 6C and 15B/15C (already described). Although it caused a low level of IPD in children aged <5 years (five to nine isolates during each of the years 2008–2009 and 2011–2012), serotype 6C was composed of two different CCs, four of which were represented by single isolates (Fig. 2; Table 1). The absence of recovered serotype 6C IPD isolates during 2013 possibly reflects the previously predicted PCV13 cross-protection against serotype 6C [13].

We have verified that all of the depicted CCs shown for serotype 6C (Fig 2), except for CC3813, have been previously associated with serotypes 6A and 6B, including CC473 found in the single serotype 6A isolate and CC1092 from the single serotype 6B isolate recovered during the 5 years of the study (both recovered during 2008) [7], [10]. No serotype 6A-mediated or serotype 6B-mediated IPD was observed during 2009–2013 in children aged <5 years, presumably because of the protection of PCV7 (during 2009) and PCV13 (during 2012–2013). Serotype switching events targeting the *cps*-internal *wciN* genes that distinguish serotypes 6A and 6C appear to have occurred many times between the different serogroup 6 lineages [7].

### Serotype 15A

Most of these isolates were within CC63, which is characteristic of the global PMEN clone PMEN-23 (http://www.pneumogen.net/pmen/). This CC gradually became predominant within ABCs serotype 15A during 2001–2007 [10]. CC63 strains contained highly related PBP patterns that were generally associated with intermediate resistance to penicillin and amoxycillin, with the exception of the single 24:73:114 pattern, which was associated with intermediate resistance to cefotaxime and ceftriaxone, and high-level resistance to cefuroxime. CC63 isolates were nearly uniformly resistant to erythromycin, clindamycin, and tetracycline (*ermB* and *tetM* positive), with *folA* and/or *folP* mutations conferring co-trimoxazole non-susceptibility (Table 1).

Two ST3811 (ST156 single-locus variant) type 15A isolates were recovered in the post-PCV13 period, presumably reflecting a past serotype switching event between a serotype 15A donor and a CC156 recipient strain. Both isolates were sensitive to antibiotics and featured the PBP 1:0:0 profile, which is associated with basal β-lactam sensitivity (see serotypes 4, 9N and 11B and 15B/C for additional examples of this widely shared profile in Table 1). All of the various CC156 clones described in this study (Table 1) retain pilus PI-1 positivity that is characteristic of the presumed serotype 9V/ST156 ancestral strain. The major serotype 9V complex CC156, which was predominant in the pre-PCV7 years [14], was associated with six additional serotypes besides serotype 9V in this study (Table 1), including 15A, 15B, 15C, 19A, 23B, and 35B.

Of the CC156 non-9V strains, only the serotype 19A and 35B variants expressed levels of β-lactam resistance similar to that of serotype 9V/ST156. The serotype 19A/ST156 and serotype 19A/ST166 strains each share 2b-12, which is also found in the 9V/ST156 strain, with the 19A/ST166 strain additionally carrying the CC156-associated 1a-15.

### Serotypes 23A and 23B

It is interesting that these two serotypes are completely (23A) or primarily (23B) associated with the two same CCs (CC338 and CC42). Although they feature different predominant STs within CC338, strains of both serotypes within CC338 show similar antimicrobial resistance features (Table 1).

Serotype 23B variants of CCs that are thought to have originated within other serotypes were additionally recovered (one or two isolates each of CC156 (9V), CC63 (15A), and CC433 (22F)).

### Serotype 17F

Serotype 17F isolates were associated with CC392 genotypes as in the pre-PCV7 years, with the exception that a single ST338 isolate, which was intermediately penicillin resistant and a presumed serotype switch variant, was observed. ST338 is a primary genotype of US serotype 23A strains, is a minor serotype 19A genotype (Table 1), and has been reported in the past as a major serotype 23F clone [20].

### NT IPD isolates

As was recently reported, isolates apparently lacking a capsule were rare among strains causing IPD [21], consistent with the capsule being required for establishing most invasive infections. Of the nine NT isolates recovered over the entire 5-year period, seven were of clonal clusters and *cps* locus signatures typical of commonly occurring serotypes, including 19A/CC695, 15B/CC199, 6C/CC1390, 6C/CC1292, 7F/CC191, and 8/CC1480. Although the *cps* loci of these nine isolates were not examined in detail for this study, our recent population-based findings with NT IPD isolates from patients of all ages recovered during 2006–2009 indicated that 90% of such isolates contained *cps* loci, with serotype-specific *cps* locus signatures and MLST types being highly associated with known serotypes [21]. Within the 801 isolates representing 2009, 2012, and 2013, there were only four NT isolates (Table 1). The study isolates from the 5 years included two NT ST448 isolates (recovered during 2008 and 2012) that are of a distinct pneumococcal lineage highly associated with carriage and conjunctivitis. This lineage lacks capsular biosynthetic genes, and has evolved with divergent virulence and adhesion factors [22].

### Clonal distributions of PCV13 serotypes

#### Subset PCV7

PCV7 serotypes showed high genetic diversity, considering their very small numbers throughout the time period (Table 1; Fig. 2).

For 2009, 2012, and 2013, PCV7-targeted serotypes accounted for a total of 16 isolates of serotypes 4, 9V, 18C, 19F, and 23F. Serotype 19F accounted for half of these isolates within five different CCs that each accounted for one to three isolates (Table 1).

#### Serotype 19A

The frequency of serotype 19A IPD decreased by approximately 90% in 2012 and 2013 relative to 2009 (Fig. 1), with all year 2009 CCs being either greatly decreased in incidence or not found (Fig. 2). It is noticeable, however, that the PCV7 escape variant 19A ST695 complex that was originally discovered among early post-PCV7 IPD isolates [5], [6] and that spread throughout the US during subsequent years [8], [23] showed a disproportionally higher residual IPD incidence in 2013 than the other serotype 19A complexes (Fig. 2). Three rare variants were observed within serotype 19A within CC138, CC2062, and CC558. To our knowledge, serotype 19A/CC138 strains have not been previously described, as this complex has been largely restricted to serotype 6B [14]. Serotype 19A/CC2062 represents numerous isolates recovered recently in South Africa (see http://pubmlst.org/spneumoniae/). Isolates within CC138 and CC2062 were not detected in a comprehensive analysis of year 2005–2007 ABCs serotype 19A isolates [8], [24]; however, a single serotype 19A/ST558 isolate was recovered during 2006 [8]. The two serotype 19A/558 variants described here (from 2006 and 2009) appear to indicate capsular locus switching from serotype 35B to serotype 19A, and are described in more detail below.

#### Serotype 7F

The second most abundant IPD serotype during 2008–2009, serotype 7F, was composed almost exclusively of highly uniform (PI-2 positive, antimicrobial susceptible) ST191 isolates (Fig. 2; Table 1), and its incidence declined to nearly non-detectable levels during 2013 (only two isolates recovered).

### Other PCV13 serotypes

The serotype 3/ST180 complex was associated with a small number of cases throughout 2008–2009 and 2012–2013 (5–14 isolates each year). A single ST1220 isolate was recovered during 2012.

The relatively rare IPD serotypes 1 and 5 each mainly represented single CCs, although, as shown in Table 1, there was considerable MLST variability within a small sample of the PI-2 positive CC227. The single outlier genotype ST2296 was from the single serotype 1 isolate recovered during 2013. There were no serotype 1 isolates recovered during 2011–2012.

### Identification of donor, recipient and progeny strains in serotype switch events by the use of MLST and PBP data

**Fig. 3:**
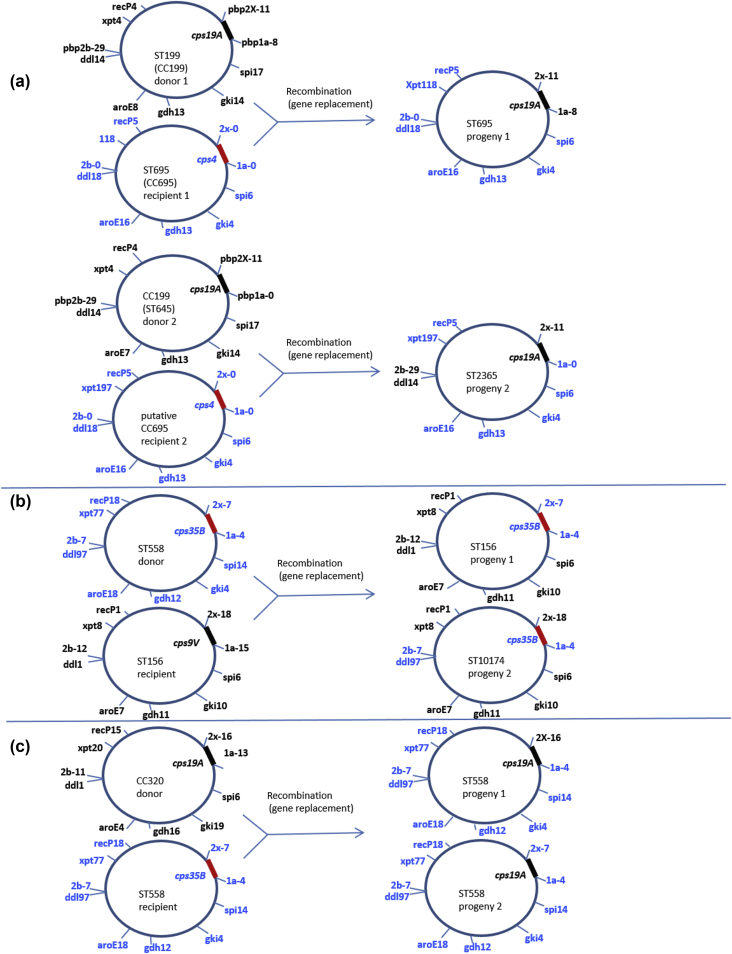
(a). Diagrammatic representations of independent and potentially PCV7 vaccination driven serotype switch involving PCV13 serotype donors (19A) and PCV7 serotype (4) recipient. (b) Two independent potential vaccine-escape events with non-vaccine serotype donor (35B) and PCV7 serotype recipient (9V). (c). Two independent serotype switch events involving non-vaccine type (35B) recipient and PCV13 type donor(s). Putative donor, recipient and progeny strains were deduced from multilocus sequence type and penicillin-binding protein profiles. Markers in the progeny that are shared with the donor share the same colour font, which differs from that in the recipient. CC, clonal complex; ST, sequence type.

The two serotype 19A/CC695 variants 19A/ST695 and 19A/ST2365 were initially discovered and described as vaccine escape variants in the post-PCV7 years [5], [24], and were both later characterized at the genomic sequence level [6], [23]. Genomic analysis of several independent isolates of these variants was performed. In addition, several independent isolates of the deduced serotype 19A/CC199 donor strains and serotype 4/ST695 recipient (recipient 1 in Fig. 3A) [6] were also identified, although a serotype 4 recipient strain corresponding to serotype 19A/ST645 donor strain 2 and serotype 19A/ST2365 progeny strain 2 was not found among ABCs surveillance isolates. Genomic comparisons of multiple isolates of the donor, recipient and progeny strains depicted in Fig. 3A revealed that each of the two progeny strains arose through an independent multifragment recombination event from serotype 19A/CC199 donor strains [6], [23]. The published *pbp1a* and *pbp2x* sequences from the putative serotype 19A/ST199 donor and serotype 4/ST695 recipients in the previous study [6] were identical within their overlaps with the entire *1a-8* and *2x-11* sequences found in all 22 19A/ST695 isolates recovered during 2009, 2012, and 2013, and also in a single serotype 19A/ST199 isolate (8:29:11) recovered during this period (Table 1). The 8:29:11 PBP profile is also represented in multiple serotype 19A/ST199 isolates in our pre-PCV7 ABCs genomic database (not shown). The sensitive PBP pattern 0:0:0 was predominant (14 of 20 isolates) among serotype 4/ST695 isolates in our database from 1998 to 1999 (whereas serotype 4/ST695 IPD isolates were common during this time, serotype 19A/ST695 isolates were not found [10], [25]). Thus, it is likely that *2x-11*, *cps19A* and *1a-8* were transferred and integrated into a serotype 4/ST695 recipient to concurrently facilitate a serotype 4 to serotype 19A switch and reduced β-lactam susceptibility (penicillin MICs of 0.06–0.12 mg/L) [6]. We observed that the *1a-0* and *2x-0* sequences from the serotype 4/ST695 isolates are identical to the sensitive TIGR4 strain, and that the donor and progeny 1 *1a-8* and *2x-11* contain mosaic sequences with substitutions previously associated with β-lactam resistance [26], [27].

In the previous study [6], another serotype 19A/CC199 (ST645, a single-locus variant of ST199) strain was identified as the likely donor in a second independent serotype 4 to serotype 19A switch. Although a CC695 recipient strain was not identified, the preponderance of serotype 19A/CC199 isolates with a 0:29:11 PBP profile (Table 1) and the observation of this same profile in serotype 19A/ST2365 (progeny 2) are indicative of co-replacement of the *cps4* and flanking 2x marker of a recipient CC695 strain with the donor *cps19A* and *2x-11*. This is also consistent with the single observed unlinked recombination event whereby the closely linked CC695 *ddl18* (exclusively found within serotype 4/CC695 strains in our database) and *2b-0* chromosomal segment was replaced by the corresponding DNA fragment from serotype 19A/CC199 containing *ddl14* and *2b-29* (Fig. 3A) [23]. We believe that the serotype 19A/ST2365 progeny 2 strain has reduced penicillin susceptibility (we have observed these strains to have MICs of 0.06–0.12 mg/L) owing to the *2x-0* to *2x-11* replacement event. The putative unlinked replacement of *2b-0* with *2b-29* is not expected to contribute to β-lactam resistance, given the lack of key substitutions within 2b-29 associated with resistance [15], even though these two pbp2b transpeptidase regions vary in six of 278 residues. The progeny 2 variant 19A/ST2365 shares the complete 0:29:11 profile with the serotype 19A/CC199 donor (Fig. 3A). These two different recombination events represent two of the previously depicted nine fragments within serotype 19A/ST2365 progeny strains that did not match known serotype 4/CC695 recipient genomes and matched a common serotype 19A/CC199 putative donor strain [23].

Another likely example (Fig. 3B) of pneumococcal conjugate vaccine escape capsular switching is shown by two putative independent switch events involving serotype 35B/ST558 and serotype 9V/ST156 as donor and recipient, respectively. The serotype 35B/ST156 (progeny 1 strain in Fig. 3B) retained all seven ST156 MLST alleles and the *2b-12* characteristic of this complex; however, *1a-4* and *2x-7* were apparently co-integrated with *cps35B* into this progeny 1 strain during the double-crossover serotype replacement event. In the progeny 2 strain (serotype 35B/ST10174), it appears that the region encompassing *cps9V* and its flanking *1a-15* was replaced by *cps35B* and its flanking *1a-4*, whereas *2x-18* flanking the opposite end of *cps9V* was unaffected during this replacement event. As in the unlinked replacement event described in the Fig. 3A progeny 2 strain, a second, unlinked, double-crossover event seems likely to have occurred within this progeny strain, resulting in replacement of the CC156 *ddl-1* and neighbouring *2b-12* with *pbp2b-7* and *ddl-97*. Besides this serotype 35B/ST10174 variant, the *ddl-97* allele has been exclusively associated with ABCs 35B/CC558 strains during the past 20 years (data not shown).

A third example of a likely serotype switch strain set (donor, recipient, two progeny strains) is shown in Fig. 3C (serotype 35B to serotype 19A). Both serotype 19A/ST558 isolates are depicted in Table 1 and in Fig. 3C. Each isolate has two or three of the PBP alleles within pattern 4:7:7, and susceptibility profiles that are found in most common serotype 35B/ST558 isolates (Table 1). As in the case with the putative serotype 9V to serotype 35B replacement event depicted in Fig. 3A, the differing PBP profiles of the progeny strains are suggestive of two independent serotype switch events: one involving co-replacement of *cps35B* and *2x-7* with *cps19A* and *2x-16* (progeny 1, recovered during 2006), and the other solely involving replacement of *cps35B* with *cps19A* (progeny 2, recovered during 2009). Although the predominance of 2x-16 within the serotype 19A/CC320 strain (Fig. 1) that was highly prevalent during the pre-PCV13 period is strongly suggestive of its role as a donor strain in this switch event, the PBP pattern data of the progeny 2 serotype 19A strain does not provide evidence of the 19A donor strain genotype.

### Removal of strains positive for pilus determinants and resistance genes by continued vaccination

**Fig. 4:**
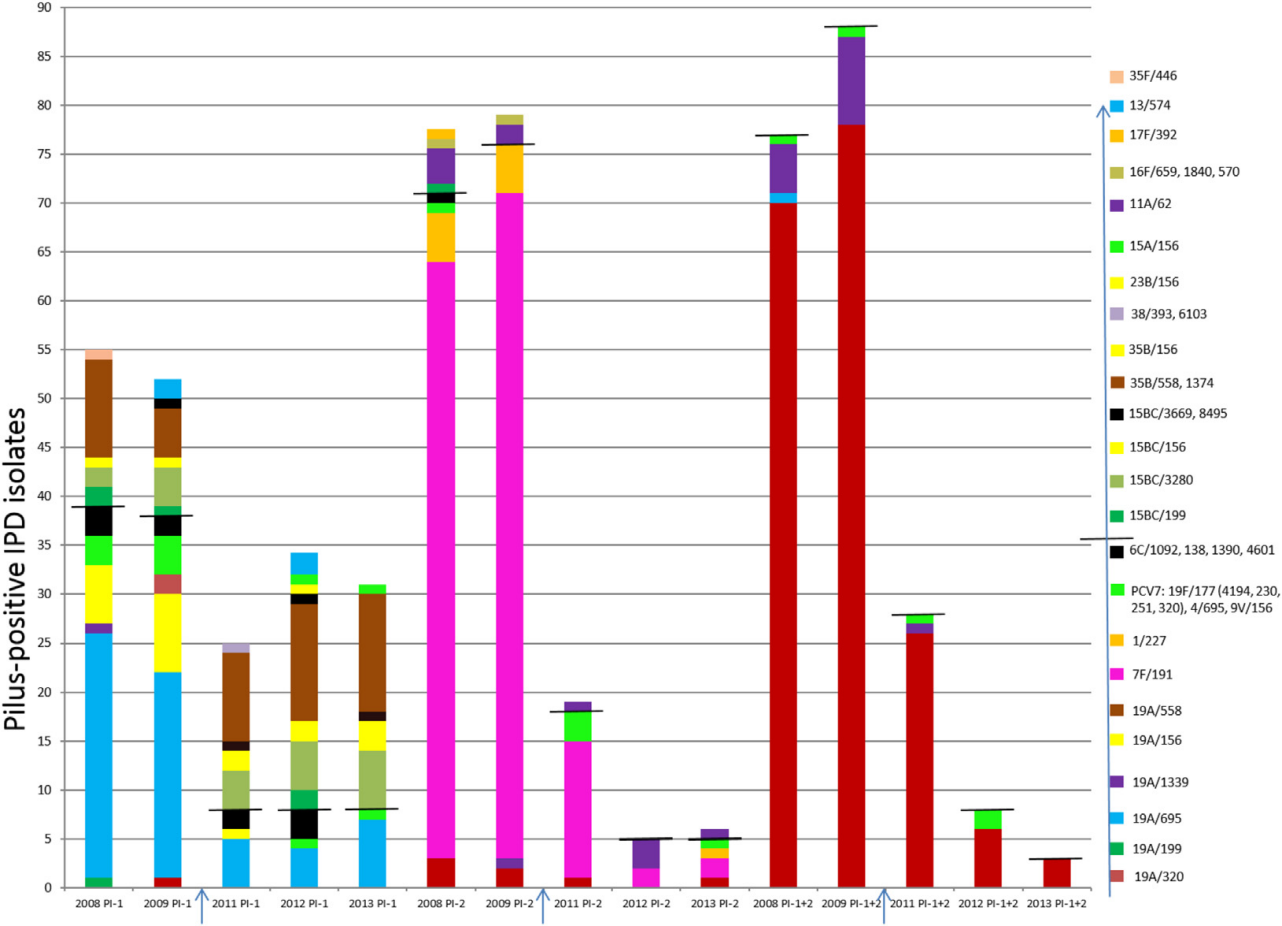
Decreases in the incidence rates of invasive pneumococcal disease (IPD) isolates positive for pilus determinants associated with decreases in pneumococcal clonal complexes after 13-valent pneumococcal conjugate vaccine (PCV13) implementation (2011–2013) in early 2010 (arrows). Bars 1–5 and 6–10 from the left indicate isolates positive for only PI-1 and PI-2, respectively. Bars 11–15 indicate isolates positive for both PI-1 and PI-2. A horizontal line on each bar indicates the cut-off between PCV13 serotype plus 6C strains (below the line) and non-PCV13 serotype strains (above the line). PCV7, seven-valent pneumococcal conjugate vaccine.

Concurrently with the dramatic decrease in the incidence of IPD that occurred in the period 2011–2013, there were decreases in the incidence rates of strains genetically positive for all three categories of pilus determinants, including PI-1-positive strains, PI-2-positive strains, and strains positive for both PI-1 and PI-2 (Fig. 4). The incidence of strains positive for only PI-1 decreased by 32–55% in 2008–2009 relative to 2011–2013, primarily because of the removal of the serotype 19A/ST695 and serotype 19A/ST156 strains. A small increase in the incidence of the PI-1 positive NVT serotype 35B complex was observed. The incidence of strains positive for PI-2 decreased even more dramatically post-PCV13, owing to the decrease in the incidence of the serotype 7F/ST191 complex. Serotype 19A/ST320 accounted for almost all of the isolates that were positive for both PI-1 and PI-2 throughout all years, and was the sole positive strain detected during 2013 after a decrease of approximately 97% relative to 2009. The residual strains positive for both PI-1 and PI-2 during 2011–2012 were also of PCV13 serotypes, including serotype 19A/ST1339 and the rarely occurring PCV7 serotype 19F strains that are highly related to 19A/ST320 (see Table 1 for strain features).

**Fig. 5:**
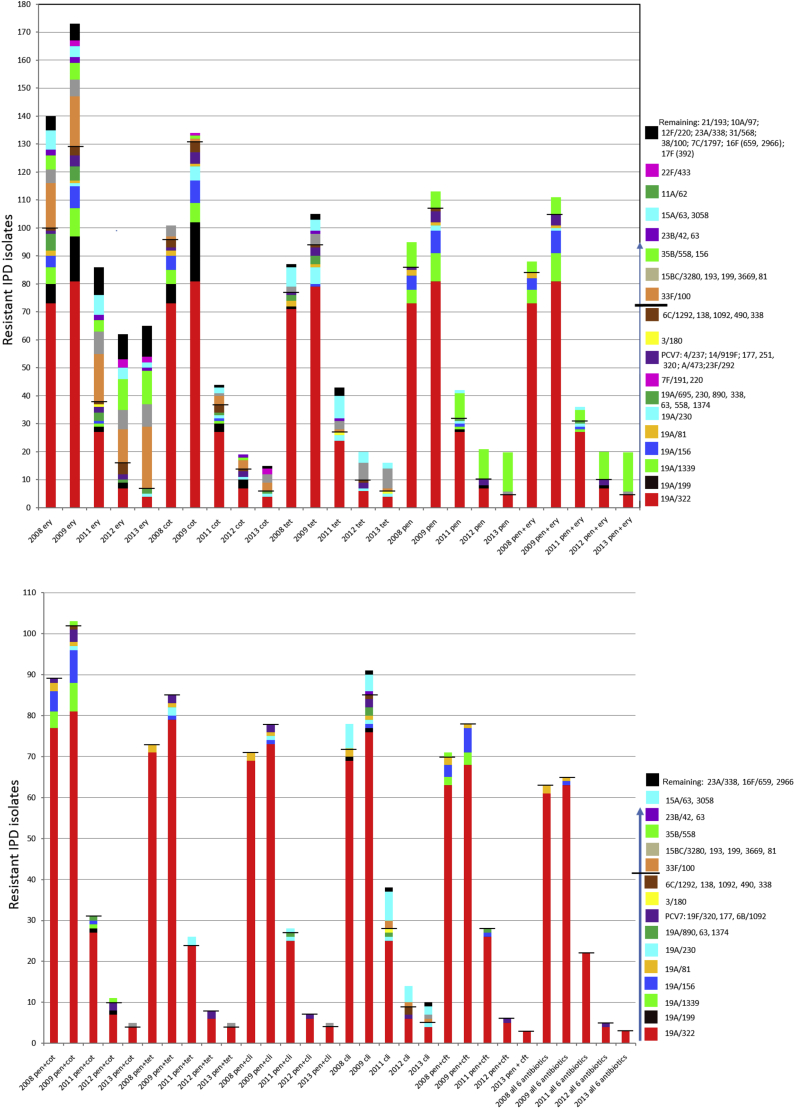
Decreases in antimicrobial resistance to six different antimicrobials and their combinations associated with decreases in the incidence rates of pneumococcal clonal complexes after 13-valent pneumococcal conjugate vaccine (PCV13) implementation in 2010. The horizontal line on each bar indicates the cut-off between PCV13 serotype plus 6C strains (below the line) and non-PCV13 serotype strains (above the line). Note that intermediate resistance is not included. co-trimoxazole; ery, erythromycin; pen, penicillin; tet, tetracycline; cli, clindamycin; cft, cefotaxime.

The major overall contributor to all of the 11 categories of resistance throughout the period 2008–2009 was serotype 19A/CC320 (Fig. 5). Resistance during this period became more common among pneumococci, because of a combination of additional serotype 19A CCs and several other residual PCV7 CCs. The major contributor to the penicillin resistance phenotype (with and without erythromycin resistance) during 2012–2103 was serotype 35B/CC558, which showed a moderate increase relative to previous years. Serotype 33F/CC100 became the predominant erythromycin-resistant/clindamycin-sensitive IPD strain complex during 2013. The incidence of isolates that were erythromycin resistant (owing to *mef* and/or *ermB* determinants) decreased by 56–60% during 2012–2013 relative to 2009, whereas the clindamycin resistance conferred by *ermB* decreased by 81–87% (*ermB* co-confers erythromycin resistance in almost all strains). Other resistance phenotypes showed similar dynamics, with serotype 19A/CC320 and other PCV13 CCs declining, leaving non-PCV13-targeted serotypes as the residual contributors to resistance. The last six combinations of resistance depicted in Fig. 5 were largely eliminated through removal of serotype 19A/CC320, with no indications of new emergent complexes. The only strains that were resistant to all six antimicrobials depicted in Fig. 5 (penicillin, cefotaxime and/or ceftriaxone, erythromycin, clindamycin, co-trimoxazole and tetracycline) during 2008–2013 were serotype 19A/CC320 (many isolates) serotype 19A/CC81 (four recovered during 2008–2009), serotype 19F/CC320 (one recovered during 2012), and serotype 15C/CC81 (one recovered during 2013). Representatives of these strain sets are described in Table 1.

### Resistance determinants

Among the 1428 isolates from the 5 years of the study, there was no observed resistance to fluoroquinolones, rifampin, linezolid, telithromycin, quinupristin–dalfopristin, or vancomycin. Of these 1428 isolates, 724 were subjected to WGS analysis and our automated algorithm for detecting specific resistance determinants. Only two of the 104 instances of combined resistance to erythromycin and clindamycin were not attributable to *ermB* (Table 1). In one (serotype 6C/ST138), the A2061G substitution, which is known to confer resistance to macrolides and lincosamides (Table S2), was detected. In the second, no causal mechanism was detected (serotype 19A/ST1756). In one isolate (serotype 19A/ST320), *ermB* was detected; however, the isolate was clindamycin sensitive (Table 1). The erythromycin-resistant/clindamycin-susceptible and chloramphenicol-resistant phenotypes in these isolates were entirely accounted for by the *mef* (166 instances) and *cat* (four instances; see serotypes 3/CC180, 15BC/CC81, 19A/CC81, and 16F/CC2966) determinants, respectively (Table 1). Tetracycline resistance was associated with *tetM* in all instances; however, three of the 134 *tetM*-positive isolates were tetracycline sensitive (two serotype 19A/ST320 and one serotype 15A/ST63). Intermediate to full co-trimoxazole resistance was always attributable to mutations within one or both of *folA* and *folP*. Most instances (107/112) of intermediate resistance were due to insertions within a hotspot of *folP*, with the other five instances being attributed to the well-known *folA* I100L substitution (Table S2). Full co-trimoxazole resistance was attributed to two unlinked mutations (*folP* insertion and *folA* I100L) in 159 of 160 instances (Table 1). In one resistant isolate, only a *folP* insertion was observed (serotype 19A/ST10160).

### Predicting β-lactam MICs from PBP patterns

The utility of composite PBP transpeptidase domain profiles as an additional genetic and phenotypic marker in tracking recombination events is apparent (see above). These profiles are also useful for rapidly assessing MICs for different β-lactam antibiotics. For example, 83 isolates in Table 1 that represented seven different CCs shared profile 2:0:2 and were basally β-lactam sensitive. There were numerous additional instances of basally sensitive profiles, several of which were very common. There was no instance of a basally sensitive profile being seen among strains showing resistance or reduced susceptibility to β-lactams. As expected, most PBP profiles associated with β-lactam resistance were highly associated with specific CCs. The association of eight basally sensitive CC156 variants (presumably descendants of the highly resistant serotype 9V/ST156 strain) with two common sensitive profiles (0:0:0 and 1:0:0) is consistent with the notion that clonally associated genetic determinants other than PBP transpeptidase-encoding sequences may not commonly play dominant roles in β-lactam resistance phenotypes. Also consistent with the dominant role of PBP transpeptidase regions in conferring β-lactam resistance is the observation that PBP pattern 15:12:18 was solely found among β-lactam-resistant strains of both CC156 and CC81 (serotype 9V/ST156, serotype 19A/ST2346, and serotype 15B/ST83) that shared closely similar β-lactam MICs (data not shown). Within given PBP patterns, there was little deviation evident in the β-lactam resistance profiles for the five different classes of antibiotics shown. When profiles SSSSS and ISSSS were shared within isolates sharing a PBP profile (e.g. 0:29:11, shared by many serotype 19A/CC199 isolates), the penicillin MICs for susceptible and intermediate isolates were always 0.06 mg/L and 0.12 mg/L, respectively. Similar observations of a one dilution difference were generally observed throughout there data when both intermediate and resistant, or both resistant and highly resistant, MIC results for the different β-lactams were observed for isolates sharing the same PBP transpeptidase profiles (data not shown).

## Discussion

The most important strain characteristics tracked by IPD strain surveillance have not changed in decades. At present, the most critical features required for vaccine evaluation and planning and treatment recommendations are still the capsular serotypes and antimicrobial susceptibilities. Nonetheless, population-based assessment of invasive pneumococcal strain features at a deeper level within individual serotypes can potentially detect undesirable developments within this very adaptable species that could undermine prevention efforts. For example, although this work provides reassuring evidence that the various lineages of the genetically complex serotype 19A appear to have either disappeared or to cause only a small residual level of IPD, it is conceivable that an antigenic variant of serotype 19A could escape being targeted by PCV13. It would be logical that such a variant would be initially detectable as a minor genetic lineage within the serotype that shows an IPD incidence that is inconsistently elevated as compared with other lineages of the same serotype. Serotype 6C is an example of a serotype 6A immunological variant that escaped notice for decades, owing to its serological similarity to serotype 6A [28]. The detection of serotype 6C with monoclonal antibody-based methodology [28] allowed the use of simple PCR-based methods [7] to easily discern serotype 6C from serotype 6A, and to allow serotype 6C inclusion for retrospective and prospective surveillance. This serotype was almost never detected in children prior to PCV7 implementation; however, it became readily detectable, although not common, in paediatric IPD patients after PCV7 implementation. Serotype 6C also became a commonly carried serotype in children [29], and has become a common cause of IPD in adults [2]. The lack of serotype 6C IPD isolates recovered in this study during 2013 from the paediatric population gives hope that a protective effect might eventually be seen in adults.

The invasive serotype 35B is presently composed primarily of one CC that is potentially poised to take advantage of reductions in the frequencies of other strains in the carriage reservoir that are occurring in the post-PCV13 era. In some ways, this can be compared with the serotype 19A situation immediately prior to PCV7 implementation. Like the most frequent serotype 19A IPD-causing CC during 1999 (CC199), the major serotype 35B/CC558 strain set caused a moderate level of IPD during the pre-PCV13 period, was β-lactam NS, and has shown indications of increased carriage during recent years [29]. It is now conceivable that, as the major β-lactam NS CC in the nasopharyngeal reservoir, and with the removal of most PCV7 and PCV13 serotype competitors, serotype 35B/CC558 and perhaps other variants, such as serotype 35B/CC156, will thrive. Although the serotype 35B/CC156 variant is very rare, we did not detect serotype 19A/CC320 strains, despite extensive genotypic surveillance of paediatric ABCs isolates during 1999 and 2001 [14], [25]; yet this complex became the single most common cause of IPD among people of all ages during the mid-2000s to late 2000s [8]. It is with some concern that two independent invasive serotype 35B/CC156 isolates (ST156 and its single-locus variant ST9910) have already been recovered during ongoing year 2015 IPD strain surveillance (data not shown). The serotype 19A/CC695 complex, which originated after PCV7 implementation from independent serotype 4 to serotype 19A switch events [5], [6], [8], [23], [24], also emerged significantly in a very short period after PCV7 implementation [8]. The two serotype 35B/CC156 strains described in this study are likely to be independent immune escape variants (serotype 9V to serotype 35B) that occurred after PCV7 implementation. In contrast, the two putative instances of serotype switch that involved replacement of the NVT *cps35B* locus with the PCV13 type *cps19A* locus (Fig. 3C) are consistent with naturally occurring intraspecies transfers of capsular biosynthetic and PBP genes, which were shown to be widespread between commonly occurring strains before conjugate vaccine implementation [30].

It is interesting that four of the current non-vaccine serotypes appeared to concurrently undergo a clonal shift during the years between the implementation of PCV7 and the implementation of PCV13 [10]. Each of serotypes 15A, 35B, 23A and 6C were primarily associated with penicillin-susceptible IPD isolates during 1999–2001. Within each of serotypes 15A, 35B, and 23A, single penicillin-NS CCs (serotypes 15A/CC63, CC35B/CC558, and 23A/CC338) gradually became predominant during subsequent post-PCV13 years [10]. Serotypes 15B and 15C showed increased antimicrobial resistance and genetic complexity as compared with the pre-conjugate vaccine era, and is potentially poised to significantly emerge in the post-PCV13 era. It remains to be determined whether post-PCV13 clonal shifts will occur within the two predominant non-vaccine serotypes, 22F and 33F, that are each primarily represented by single β-lactam-susceptible CCs.

The primary purpose of this work was to provide a thorough descriptive reference for important features of IPD strains afflicting our paediatric population. The secondary aim was to implement a more informative and efficient method for the routine determination of serotypes, resistance features, pilus determinants, and genotypes. WGS-based serotype deduction was successful because there is remarkable sequence conservation within the *cps* loci for individual serotypes. An example of this conservation is given by the *wcwD7F* locus used for distinguishing serotype 7F from serotype 7A, where inactivating mutations distinguish the rarely occurring serotype 7A from serotype 7F. Of the 75 Quellung-typed serotype 7F isolates examined, 74 shared a perfectly conserved 9630bp *wcwD7F*, and one contained a single missense (A25V) base substitution. We feel that harnessing our first version of an automated WGS pipeline allows us to monitor the changes within IPD strains that are primarily driven by horizontal genetic transfer events at a much deeper and detailed level. For example, although serotype switch events can readily be identified within isolate sets through performing multilocus sequence typing and serotyping, the examination of switch variants in more detail will allow better elucidation of donor lineages, and identification and tracking of unlinked determinants that are often simultaneously co-transferred during these events. Genomic analyses of pneumococcal strains indicate that the term ‘serotype switch’ is an oversimplification, and that it often involves simultaneous recombination events at multiple unlinked chromosomal loci [4], [18], [23].

Removing the primary IPD-causing serotypes concurrently removed from the pneumococcal population strain reservoirs for resistance and adhesion that are likely to serve as advantages in carriage. It follows that additional advantageous features within the species are removed or depleted from the carriage reservoir through this strategy. It is hoped that the durability of the protection afforded by PCV13 will approximate that of PCV7, which, as compared with the pre-vaccination years, reduced the incidence of paediatric IPD throughout the 2000s by approximately 80% [2], [3].

## Transparency declaration

The authors declare that they have no conflicts of interest.

## Figures and Tables

**Table 1:** Summary of key features of year 2009, 2012 and 2013 IPD isolates: WGS-based identification of serotype, multilocus sequence types, pilus subunit detection, penicillin-binding protein transpeptidase profiles, and resistance determinants correlated with phenotypic data^a^

Serotype, isolates characterized in years 2009:2012:2013 b	Reference CC ST^c^	CC percentage of the serotype during		ST^d^ (relation to reference CC ST)^e^ (no. identified)	PI-1, PI-2 (no. when variation)	PBP 1a:2b:2x profile (no. when variation)	β-Lactam R profile^f^ (no. when variation)	Non-β-lactam R profile^g^ (no. when variation)	Resistance determinant profile h (no. characterized as shown^a^)
2009	2012–2013
1, 7:0:1 (6:0:1)	227	85.7	100	227 (4)	–, +	23:0:2	SSSSS	SSSSS	Negative (4)
304 (TLV) (1)	–, +	23:4:2	SSSSS	SSSSS	Negative (1)
306 (DLV) (1)	–, +	23:6:5	SSSSS	SSSSS	Negative (1)
2296	14.3	0	2296 (1)	–, –	0:4:0	SSSSS	SSSSS	Negative (1)
3, 13:5:9	180	100	93.3	180 (18)	–, –	2:3:2 (15) 2:0:2 (3)	SSSSS	SSSSS	Negative (17)
RRSRR	*ermB*, *tetM*, *cat* (1)
10179 (SLV) (1)	–, –	2:3:2	SSSSS	SSSSS	Negative (1)
1220	0	6.7	1220 (1)	–, –	62:0:0	SSSSS	SSSSS	Negative (1)
4, 1:1:2	695	100	33.3	899 (SLV) (1)	+, –	0:0:3	SSSSS	SSSSS	Negative (1)
205 (TLV) (1)	+, –	0:0:3	SSSSS	SSSSS	Negative (1)
437	0	66.7	10173 (TLV) (1)	–, –	1:0:0	SSSSS	SSISS	*folP* insertion (1)
10172 (DLV) (1)	–, –	3:0:0	SSSSS	SSISS	*folP* insertion (1)
5, 2:0:0	289	100	0	Not done (2)	–, –	Not done	SSSSS	SSSSS	Not done (2)
6C, 9:5:0	1092	11.1	40.0	1092 (2)	+, –	6:7:8	IISSR	SSRSS	*folA* I100L + *folP* insertion (2)
15:7:8	RRIIH
490 (SLV) (1)	+, –	1:4:2	SSSSS	RSISS	*mef*, *folA* I100L (1)
138	22.2	40.0	138 (2)	+, –	2:0:3	SSSSS	RRSSS	23S-A2061G (1)
0:0:3	SSSSS	SSISS	*folP* insertion
639 (DLV) (1)	–, –	2:0:0	SSSSS	RSSSS	*mef* (1)
4343 (SLV) (1)	+, –	Not done	SSSSS	SSRSS	Not done (1)
1292	22.2	0	1292 (1)	–, –	19:31:8	IISSR	RSRSS	*mef*, *folA* I100L + *folP* insertion (1)
9455 (SLV) (1)	–, –	19:31:8	IISSS	RSRSS	*mef*, *folA* I100L + *folP* insertion (1)
473	22.2	0	473 (1)	–, –	0:4:2	SSSSS	SSSSS	Negative (1)
5501 (SLV) (1)	–, –	1:0:21	SSSSS	SSSSS	Negative (1)
338	11.1	20.0	338 (1)	–, –	Not done	ISSSS	SSISS	Not done (1)
2777 (SLV) (1)	2:1:1	ISSSS	SSSSS	Negative (1)
4343 (SLV) (1)	–, –	Not done	SSSSS	SSISS	Not done (1)
395	11.1	0	395 (1)	–, –	2:6:0	SSSSS	SSSSS	Negative (1)
7C, 0:1:1	1797	0	100	1797 (1)	–, –	2:4:6	SSSSS	SSSSS	Negative (1)
10151 (SLV) (1)	–, –	2:4:6	SSSSS	SSSSS	Negative (1)
7F, 71:3:2	191	97.2	80.0	191 (62)	–, +	2:0:2	SSSSS	SSSSS	Negative (62)
9072 (SLV) (1)	–, +	2:0:42	SSSSS	SSSSS	Negative (1)
10162 (SLV) (1)	–, +	2:0:2	SSSSS	SSSSS	Negative (1)
220	2.8	20.0	218 (SLV) (1)	–, –	2:0:2	SSSSS	SSSSS	Negative (1)
1176 (TLV) (2)	–, –	2:0:2	SSSSS	SSSSS	Negative (2)
8, 3:4:2	1480	66.7	100	1480 (6)	–, –	3:5:5 (5) 63:5:5 (1)	SSSSS	SSSSS	Negative (6)
1268 (1)	–, –	3:5:5	SSSSS	SSSSS	Negative (1)
53	33.3	0	10155 (SLV) (1)	–, –	3:6:5	SSSSS	SSSSS	Negative (1)
9N, 4:2:0	66	50.0	100	66 (3)	–, –	1:0:0 (2) 1:70:66 (1)	SSSSS	SSSSS	Negative (3)
632 (SLV) (1)	–, –	1:0:0	SSSSS	SSSSS	Negative (1)
1257	25.0	0	1257 (1)	–, –	1:4:2	SSSSS	SSSSS	Negative (1)
4666	25.0	0	4666 (1)	–, –	23:0:0	SSSSS	SSSSS	Negative (1)
9V, 2:0:0	156	2 (100)	0	156 (2)	+, –	15:12:18	RIIIH	RSRSS	*mef*, *folA* I100L + *folP* insertion (1)
HRIRH	RSRSS	*mef*, *folA* I100L + *folP* insertion (1)
10A, 8:8:4	97	87.5	91.7	816 (DLV) (12)	–, –	0:0:2	SSSSS	SSSSS	Negative (11)
RSSSS	*mef* (1)
97 (4)	–, –	0:0:2 (2) 0:40:2 (2)	SSSSS	SSSSS	Negative (4)
585 (SLV) (1)	–, –	0:0:2	SSSSS	SSSSS	Negative (1)
3617	12.5	0	3617 (1)	–, –	23:4:0	SSSSS	SSIRS	*folP* insertion, *tetM* (1)
3735	0	8.3	10177 (DLV) (1)	–, –	2:0:0	SSSSS	SSISS	*folP* insertion (1)
11A, 5:6:2	62	100	100	62 (8)	–, + (3) –, – (5)	2:6:0 (5) 2:6:38 (1) 2:6:10 (2)	SSSSS	SSSSS	Negative (2)
RSSSS	*mef* (2)
RSRSS	*mef*, *folA* I100L + *folP* insertion (1)
SSISS	*folA* I100L (1) *folP* insertion (1)
SSRSS	*folA* I100L + *folP* insertion (1)
10154 (SLV) (1)	–, –	0:6:10	SSSSS	RSRSS	*mef*, *folA* I100L + *folP* insertion (1)
11B, 0:0:1	8954	0	1 (100)	10153 (DLV) (1)	–, –	1:0:0	SSSSS	RSRSS	*mef*, *folA* I100L + *folP* insertion (1)
12F, 7:7:7	220	85.7	100	220 (8)	–, –	0:0:2 (6) 0:0:17 (4)	SSSSS	SSSSS	Negative (8)
RSSSS	*mef* (1)
SSISS	*folP* insertion (1)
218 (SLV) (6)	–, –	0:0:0 (5) 0:0:2 (1)	SSSSS	RSSSS	*mef* (2)
RSISS	*mef*, *folA* I100L (1)
SSSSS	Negative (3)
10171 (DLV) (1)	–, –	0:0:0	SSSSS	RSISS	*mef*, *folP* insertion (1)
1176 (TLV) (1)	–, –	Not done	SSSSS	SSSSS	Not done
3776	14.3	0	3776 (1)	–, –	23:4:1	SSSSS	SSSSS	Negative (1)
13, 2:0:0	574	100	0	574 (1)	+, –	11:0:0	SSSSS	SSSSS	Negative (1)
9462 (SLV) (1)	+, –	11:0:0	SSSSS	SSSSS	Negative (1)
15A, 5:5:3	63	80.0	75.0	63 (4)	–, –	24:27:28 (2)	ISSSS	RRIRS	*ermB*, *folP* insertion, *tetM* (2)
24:27:35 (1)	IISSS	RRS**S**S	*ermB*, *tetM* (1)
24:73:114 (1)	IISIH	RRIRS	*ermB*, *folP* insertion, *tetM* (1)
9939 (SLV) (3)	–, –	24:27:28 (1) 24:22:28 (2)	ISSS	RRIRS	*ermB*, *folP* insertion, *tetM* (3)
3058	20.0	0	Not done (1)	–, –	Not done	SSSSS	SSSSR	Not done (1)
156	0	25.0	3811 (SLV) (2)	+, –	1:0:0	SSSSS	SSSSS	Negative (2)
15B + 15C, 14:24:28 (14:24:26)	199	35.7	47.0	199 (8)	–, –	2:0:2	SSSSS	SSSSS	Negative (8)
RSSSS	*mef* (1)
0:0:0	SSSSS	SSSSS	Negative (2)
2:0:0	SSSSS	SSSSS	Negative (2)
RSSSS	*mef* (1)
2:0:12	SSSSS	SSSSS	Negative (2)
1:0:0	SSSSS	SSSSS	Negative (1)
1:0:6	SSSSS	SSSSS	Negative (1)
5:0:2	SSSSS	SSSSS	Negative (1)
200 (SLV) (1)	–, –	1:0:0	SSSSS	SSSSS	Negative (1)
10156 (DLV) (1)	–, –	0:0:0	SSSSS	SSSSS	Negative (1)
3280	28.6	23.5	3280 (10)	+, – (10) –, – (1)	7:8:9	IISSS (9) ISSSS (1) IISSR (1)	RSIRS (10)	*mef*, *folP* insertion, *tetM* (10)
RSIIS (1)	*mef*, *folP* insertion (1)
10611 (SLV) (3)	+, –	7:8:9	IISSS	RSISS	*mef*, *folP* insertion, *tetM* (3)
7479 (SLV) (2)	+, –	7:8:9	IISSS	RSISS	*mef*, *folP* insertion, *tetM* (2)
8300 (DLV) (1)	–, –	7:1:30	ISSSS	SSRSS	*folA* I100L + *folP* insertion (1)
1262	7.1	13.7	1262 (7)	–, –	2:0:2 (6) 14:0:2 (2) 2:0:31 (1)	SSSSS	SSISS (7)	*folP* insertion (7)
SSSSS (2)	Negative (2)
156	7.1	9.8	162 (SLV) (5)	+, –	1:0:0	SSSSS	SSSSS	Negative (5)
608 (SLV) (1)	+, –	2:12:2	SSSSS	SSISS	*folA* I100L (1)
346	7.1	2.0	346 (2)	–, –	20:18:35	IISSS	SSSSS	Negative (2)
81	0	2.0	83 (SLV) (1)	–, –	15:12:18	HRIIH	RRRRR	*ermB*, *folA* I100L + *folP* insertion, *tetM*, *cat* (1)
193	14.3	0	10227 (SLV) (1)	–, –	0:0:0	SSSSS	RSISS	*mef*, *folA* I100L (1)
8495	0	2.0	8459 (1)	+, –	Not done	SSSSS	RSRSS	*mef*, *folP* insertion (1)
16F, 7:1:6 (7:1:5)	659	71.4	50.0	659 (2)	–, – (1) –, + (1)	9:0:3	SSSSS	SSSSS	Negative (2)
3157 (DLV) (1)	–, –	0:0:3	SSSSS	SSSSS	Negative (1)
995 (SLV) (2)	–, –	9:4:3	SSSSS	SSSSS	Negative (2)
10170 (SLV) (1)	–, –	9:0:3	SSSSS	SSSSS	Negative (1)
10152 (DLV) (1)	–, –	9:4:3	SSSSS	RSSSS	*mef* (1)
10224 (DLV) (1)	–, –	9:4:3	SSSSS	SSSSS	Negative (1)
1840	14.3	33.3	1840	–, –	0:10:2	SSSSS	SSSSS	Negative (1)
10148 (SLV) (2)	–, –	0:10:14	SSSSS	SSSSS	Negative (2)
570	0	16.7	570	–, –	0:6:2	SSSSS	SSSSS	Negative (1)
2966	14.3	0	2966	–, –	37:4:2	SSSSS	RRSRR	*ermB*, *tetM*, *cat* (1)
17F, 4:0:3	392	75.0	100	392	–, –	11:0:0	SSSSS	SSSSS	Negative (3)
RSSSS	*mef* (1)
10176 (SLV)	–, –	11:0:0	SSSSS	RSSSS	*mef* (1)
338	25.0	0	338	–, –	Not done	ISSSS	SSISS	Not done (1)
18C, 0:1:0	496	0	100	496	–, –	0:0:0	SSSSS	SSSSS	Negative (1)
19A, 190:20:14 (189:20:14)	320	42.9	32.4	320	+, + (66)	13:11:16 (58) 13:14:16 (1)	HHRRH (46) HHHRH (5) HHRIH (5) RHRIH (2) RHRRH (1) RRRSH (1)	RRRRS (63) R**S**RRS (1) RRR**S**S (2)	*mef*, *ermB*, *folA* I100L + *folP* insertion, *tetM* (66)
13:14:26 (3) 13:11:33 (2)	HHRHH (5)
13:11:26 (1)	RHRHH (1)
1451 (SLV) (16)	+, +	13:11:16 (12) 13:14:16 (1)	HHRRH (11) HHRIH (1) RHRIH (1)	RRRRS	*mef, ermB*, *folA* I100L + *folP* insertion, *tetM* (15) *mef, ermB*, *folA* I100L, *tetM* (1)
13:28:37 (1) 13:11:113 (1)	HHHHH (2)
271 (SLV) (2)	+, +	13:14:20	HHRIH	RRRRS	*mef, ermB*, *folA* I100L + *folP* insertion, *tetM* (2)
2514 (SLV) (2)	+, +	13:11:16	HHRIH HHRRH	RRRRS	*mef, ermB*, *folA* I100L + *folP* insertion, *tetM* (2)
9356 (SLV) (2)	+, +	13:11:16	HHRIH HHRRH	RRRRS	*mef, ermB*, *folA* I100L + *folP* insertion, *tetM* (2)
9630 (SLV) (1)	+, +	13:11:16	HHRRH	RRRRS	*mef, ermB*, *folA* I00L + *folP* ins, *tetM* (1)
10143 (SLV) (1)	+, +	13:11:16	HHRHH	RRRRS	*mef, ermB*, *folA* I100L + *folP* insertion, *tetM* (1)
10223 (SLV) (1)	+, +	13:11:16	HHRRH	RSRSS	*mef*, *folA* I100L + *folP* insertion (1)
10144 (SLV) (1)	+, +	13:14:26	HHRHH	R**S**RRS	*mef, ermB*, *folA* I100L + *folP* insertion, *tetM* (1)
199^f^	27.0	23.5	199 (27)	–, –	0:29:11 (22) 33:29:11 (3) 8:29:11 (1) 32:29:11 (1)	ISSSS (21) SSSSS (5) IISSS (1)	SSSSS (10)! RSRSS (8)+ RSISS (1)+ SSRSS(6)∗ SSISS (1)# RSSSS (1)@	Negative (10)! *mef*, *folA* I100L + *folP* insertion (9)+ *folA* I100L + *folP* insertion (6)∗ *folP* insertion (1)# *mef* (1)@
18:37:8 (1)	IHRIH (1)
667 (SLV) (8)	–, –	0:0:0	SSSSS	SSSSS	Negative (8)
1756 (SLV) (2)	–, –	0:29:11	ISSSS	SSSSS (1) **RR**SSS (1)	Negative (2)
1673 (SLV) (1)	–, –	0:29:11	ISSSS	SSSSS	Negative (1)
2540 (SLV) (2)	–, –	0:29:11	SSSSS	SSSSS	Negative (1)
ISSSS	SSISS	*folP* insertion (1)
2273 (DLV) (1)	–, –	0:29:11	ISSSS	SSRSS	*folA* I100L + *folP* insertion (1)
2269 (SLV) (1)	–, –	33:29:11	ISSSS	RSRSS	*mef*, *folA* I100L + *folP* insertion (1)
4262 (SLV) (1)	–, –	8:29:8	ISSSI	RSRSS	*mef*, *folA* I100L + *folP* insertion (1)
2344 (SLV) (2)+	–, –	18:12:8	RIIIH	RSRSS	*mef*, *folA* I100L + *folP* insertion (1)
IISIH	SSRSS	*folA* I100L + *folP* insertion
695	11.1	32.4	695 (24)	+, –	8:0:11	ISSSS (20) SSSSS (4)	SSSSS (20)	Negative (20)
RSSSS (4)	*mef* (4)
2365 (TLV) (1)	+, –	0:29:11	SSSSS	SSSSS	Negative (1)
230	3.2	5.9	230 (3)	–, –	17:15:22 (2)	ISSSS	SSRRS	*folA* I100L + *folP* insertion, *tetM* (3)
17:7:8 (1)	RIISH
276 (SLV) (1)	–, –	17:15:18	HISIH	RRIRS	*ermB*, *folP* insertion, *tetM* (1)
6741 (SLV) (1)	–, –	17:15:22	ISSSS	SSRRS	*folA* I100L + *folP* insertion, *tetM* (1)
319 (SLV) (1)	–, –	10:0:13	IISSS	RRRRS	*ermB*, *folA* I100L + *folP* insertion, *tetM* (1)
3936 (SLV) (1)	–, –	17:15:22	ISSSS	SSRRS	*folA* I100L + *folP* insertion, *tetM* (1)
10160 (DLV) (1)	–, –	17:15:22	ISSSS	SS**R**RS	*folP* insertion, *tetM* (1)
1339	5.3	0	1339 (3)	+, + (2) –, + (1)	27:30:8	RRIIH (2) HIISR (1)	RSRSS	*mef*, *folA* I100L + *folP* insertion (3)
2268 (SLV) (3)	+, +	27:36:8	HHRRH (2)	RSRSS (2)	*mef*, *folA* I100L + *folP* insertion (2)
HHHRH (1)	RSISS (1)	*mef*, *folP* insertion
2270 (SLV) (1)	+, +	27:30:8	RRRIH	RSRSS	*mef*, *folA* I100L + *folP* insertion (1)
8207 (SLV) (1)	+, +	27:11:8	HHRRH	RSRSS	*mef*, *folA* I100L + *folP* insertion (1)
10178 (SLV) (1)	+, +	27:30:8	RHRIH	RSRSS	*mef*, *folA* I100L + *folP* insertion (1)
10159 (DLV) (1)	+, +	27:30:8	HRRIH	RSRSS	*mef*, *folA* I100L + *folP* insertion (1)
156	4.2	0	156 (5)	+, –	29:12:26 (4)	HRIHH	RSRSS	*mef*, *folA* I100L + *folP* insertion (5)
41:12:36 (1)	HHRIH
4026 (SLV) (2)	+, –	28:14:36	HHRIH	RSRSS	*mef*, *folA* I100L + *folP* insertion
HHRRH	RSISS	*mef*, *folP* insertion
166 (SLV) (1)	+, –	15:12:7	HRRRH	RRRRS	*ermB*, *folA* I100L + *folP* insertion, *tetM* (1)
10225 (TLV) (1)	+, –	65:38:36	HHHRH	RSRSS	*mef*, folA I100L, *folP* insertion (1)
338	3.2	2.9	8197 (DLV) (2)	–, –	17:1:22	ISSSS	SSISS	*folP* insertion (2)
338 (1)	–, –	0:1:1	ISSSS	SSSSS	Negative (1)
10150 (DLV) (1)	–, –	17:1:22	ISSIS	SSISS	*folP* insertion (1)
558^g^	1.1	0	558 (2)	+, –	4:7:7 (2009)	IRISH	RSSSS	*mef* (1)
4:7:16 (2006)	RRIIH	SSSSS	Negative (1)
66	0.5	0	66 (1)	–, –	0:0:0	SSSSS	SSSSS	Negative (1)
81	0.5	0	2346 (SLV) (1)	–, –	15:12:18	HRRRH	RRRRR	*ermB*, *folA* I100L + *folP* insertion, *tetM*, *cat* (1)
138	0.5	0	639 (DLV) (1)	–, –	2:0:0	SSSSS	SSSSS	Negative (1)
292	0.5	0	292 (1)	–, –	30:0:0	SSSSS	SSSSS	Negative (1)
2062	0	2.9	2062 (1)	–, –	16:13:19	ISSSS	SSRSS	*folA* I100L + *folP* insertion (1)
63	0.5	0	63	–, –	Not done	ISSSS	RRSRS	Not determined (1)
1374			1374 (1)	+, –	23:75:126	SSSSS	RRIRS	*ermB*, *tetM*, *folP* insertion
19F, 3:3:2	320	33.3	40.0	271 (SLV)	+, +	13:14:20 (1)	HHRIH	RRRRS	*mef, ermB*, *folA* I100L + *folP* insertion, *tetM* (3)
13:16:47 (1)	RIIIH
13:14:115 (1)	HHRHH
251	0	40.0	251 (1)	+, –	2:0:0	SSSSS	SSSSS	Negative (1)
654 (SLV) (1)	+, –	2:0:0	SSSSS	SSSSS	Negative (1)
177	1 (33.3)	0	179 (SLV) (1)	+, –	34:32:43	RISIH	RRSRS	*ermB*, *tetM* (1)
346	0	20.0	1203 (SLV) (1)	–, –	20:18:15	SSSSS	SSSSS	Negative (1)
230	33.3	0	Not done (1)	–, –	Not done	SSSSS	SSSSS	Not done (1)
21, 3:3:5 (3:2:5)	432	33.3	71.4	432 (6)	–, –	0:0:2	SSSSS	SSSSS	Negative (6)
193	66.7	28.6	193 (2)	–, –	2:0:2	SSSSS	SSSSS	Negative (2)
3689 (SLV)	–, –	2:0:2	SSSSS	SSSSS	Negative (1)
22F, 23:21:19	433	100	97.6	433 (47)	–, –	1:2:2 (42) 1:2:27 (2) 1:2:59 (3)	SSSSS	SSSSS	Negative (44)
RSSSS	*mef* (3)
7314 (SLV) (3)	–, –	1:2:2	SSSSS	RSSSS	*mef* (3)
9456 (SLV) (1)	–, –	1:0:2	SSSSS	SSSSS	Negative (1)
10166 (SLV) (1)	–, –	1:2:2	SSSSS	SSSSS	Negative (1)
10164 (SLV) (1)	–, –	1:2:2	SSSSS	RSSSS	*mef* (1)
10165 (SLV) (1)	–, –	1:2:2	SSSSS	SSSSS	Negative (1)
10167 (SLV) (1)	–, –	1:2:2	SSSSS	RSSSS	*mef* (1)
10163 (DLV) (3)	–, –	1:2:2	SSSSS	RSSSS	*mef* (2)
SSSSS	Negative (1)
10226 (DLV) (1)	–, –	1:2:2	SSSSS	RSSSS	*mef* (1)
698	0	2.4	698 (1)	–, –	0:2:0	SSSSS	SSSSS	Negative (1)
23A, 1:4:4	338	100	75.0	338 (7)	–, –	0:1:1 (5) 19:1:24 (1) 1:1:48 (1)	ISSSS (6) IISSS (1)	SSISS (3)	*folA* I100L (2) *folP* insertion (1)
SSSSS (2)	Negative (2)
RSSSS (1)	*mef* (1)
RRRSS (1)	*ermB*, *folA* I100L + *folP* insertion (1)
42	0	25.0	10147 (SLV) (1)	–, –	2:0:6	SSSSS	SSSSS	Negative (1)
1839 (DLV) (1)	–, –	12:0:0	SSSSS	SSSSS	Negative (1)
23B, 7:7:7 (7:6:7)	338	42.9	38.5	1373 (DLV) (6)	–, –	0:1:1 (5) 21:1:1 (1)	ISSSS (5) SSSSS (1)	SSISS	*folP* insertion (3)
SSSSS	Negative (3)
4534 (TLV) (1)	–, –	7:21:1 (1)	ISSSS	RSISS	*mef*, *folP* insertion (1)
10149 (DLV) (1)	–, –	7:1:1 (1)	ISSSS	SSRSS	*folA* I100L + *folP* insertion (1)
42	42.9	46.1	439 (SLV) (6)	–, –	0:0:0 (4) 0:0:11 (1) 0:24:29 (1) 0:0:119 (1)	SSSSS	SSSSS	Negative (6)
RSSSS	*mef* (1)
33 (DLV) (1)	–, –	12:0:2	SSSSS	SSSSS	Negative (1)
1847 (DLV) (1)	–, –	0:0:0	SSSSS	SSSSS	Negative (1)
156	0	7.7	162 (SLV) (1)	+, –	0:0:0	SSSSS	SSSSS	Negative (1)
63	14.3	0	63 (1)	–, –	24:27:28	IISSS	RRSRS	*ermB*, *tetM*(1)
433	0	7.7	4334 (SLV) (1)	–, –	0:2:0	SSSSS	SSSSS	Negative (1)
23F, 1:0:0	292	100	0	2520 (TLV) (1)	–, –	12:68:104	ISSSS	SSRRS	*folA* I100L + *folP* insertion, *tetM* (1)
28A, 0:0:1	494	0	1 (100)	494 (1)	–, –	2:2:2	SSSSS	SSSSS	Negative (1)
31, 0:2:1	568	0	3 (100)	568 (2)	–, –	12:0:15	SSSSS	SSSSS	Negative (2)
33F, 25:14:27 (25:14:25)	100	92.0	100	100 (14)	–, –	2:0:6 (12) 2:20:6 (1) 64:0:6 (1)	SSSSS	SSISS	*folP* insertion (6)
RSISS	*mef*, *folP* insertion (6)
SSRSS	*folA* I100L + *folP* insertion (1)
RSSSS	*mef* (1)
2705 (DLV) (34)	–, –	2:0:6 (34)	SSSSS	RSISS (30)	*mef*, *folP* insertion (30)
RSRSS (4)	*mef*, *folA* I100L + *folP* insertion (3) *mef*, *folP* insertion (1)
SSISS (1)	*folP* insertion (1)
9029 (SLV) (2)	–, –	2:0:6 (1)	SSSSS	RSISS	*mef*, *folP* insertion (1)
2:9:6 (1)	SSSSS	SSSSS	Negative (1)
717 (TLV) (2)	–, –	2:0:6 (1) 2:72:6 (1)	SSSSS	RRSRS	*ermB*, *tetM* (2)
7507 (SLV) (1)	–, –	2:0:6 (1)	SSSSS	RSISS	*mef*, *folP* insertion (1)
10145 (DLV) (1)	–, –	2:0:6 (1)	SSSSS	SSISS	*folP* insertion (1)
10168 (DLV) (1)	–, –	4:23:7	IISSR	RSISS	*mef*, *fol* insertion (1)
10142 (TLV) (1)	–, –	2:0:6 (1)	SSSSS	RSISS	*mef*, *folP* insertion (1)
10169 (DLV) (1)		2:0:6	SSSSS	RSRSS	*mef*, *folA* I100L + *folP* insertion (1)
10146 (TLV) (1)	–, –	2:0:6	SSSSS	RSISS	*mef*, *folP* insertion (1)
60	4.0	0	60 (1)	–, –	2:4:2	SSSSS	SSSSS	Negative (1)
1012	4.0	0	1012 (1)	–, –	2:0:2	SSSSS	SSSSS	Negative (1)
34, 1:1:5	547	100	83.3	547 (5)	–, –	0:0:2	SSSSS	SSSSS	Negative (5)
10157 (SLV) (1)	–, –	0:0:2	SSSSS	SSSSS	Negative (1)
1884	0	16.6	1884 (1)	–, –	0:0:0	SSSSS	SSSSS	Negative (1)
35B, 8:13:15 (8:13:13)	558	62.5	92.3	558 (17)	+, –	4:7:7 (16) 4:7:112 (1) 4:19:7 (1)	RHIIH (6) RHRIH (5) RRIIH (4) RRRIH (3)	RSSSS (17)	*mef* (17)
RSISS (1)	*mef*, *folA* I100L (1)
10222 (SLV) (1)	+, –	4:7:7	RRRIH	RSSSS	*mef* (1)
10175 (SLV) (1)	+, –	4:14:7	RHRIR	RSSSS	*mef* (1)
473	25.0	0	3822 (SLV) (1)	–, –	23:4:21	SSSSS	SSSSS	Negative (1)
156	12.5	3.9	10174 (SLV) (1)	+, –	4:7:18	RIIIH	RSRSS	*mef*, *folA* I100L + *folP* insertion (1)
156 (1)	+, –	4:12:7	RHIIH	RSRSS	*mef*, *folA* I100L + *folP* insertion (1)
452	0	3.9	10158 (SLV) (1)	–, –	0:0:0	SSSSS	SSSSS	Negative (1)
35F, 2:1:4	498	100	100	498 (3)	–, –	0:3:3	SSSSS	SSSSS	Negative (3)
1635 (DLV) (2)	–, –	0:3:3	SSSSS	SSSSS	Negative (2)
38, 13:10:12(13:10:11)	393	100	100	393 (27)	–, –	2:4:0 (19) 2:4:4 (4) 2:4:41 (1) 0:0:2 (1)	SSSSS (27)	SSSSS	Negative (26)
SSISS	*folP* insertion (1)
1140 (DLV) (1)	–, –	2:4:0	SSSSSS	SSSSS	Negative (1)
NT^i^, 2:2:0	448	0	1	448 (1)	–, –	Not done	SSSSS	SSSSS	Not done (1)
	1292	1	0	Not done (1)	–, –	Not done	SSSSS	SSSSS	Not done (1)
	1480	1	0	1480 (1)	–, –	3:5:5	SSSSS	SSSSS	Negative (1)
	199	0	1	199 (1)	–, –	2:0:2	SSSSS	SSSSS	Negative (1)

CC, clonal complex; DLV, double-locus variant; H, high-level resistance; I, intermediate resistance; PBP, penicillin-binding protein; R, resistance; S, susceptibility; SLV, single-locus variant; ST, sequence type; TLV, triple-locus variant.

^i^Features of a serotype 19A/ST558 strain recovered during 2006 [19] are also shown.

a Whole genome sequence (WGS)-based data are in columns 1, 5, 6, 7, and 10. There was a perfect correlation between Quellung-based and WGS-based serotype determination (column 1), except for three of the four non-typeable isolates at the bottom, for which the indicated serotype-specific signatures were found from genomic sequences (see footnote i). The numbers for which serotypes and the other indicated parameters were determined with WGS as well as with Quellung are indicated in the last column shown. To simplify the table, minimal data are added for isolates characterized solely through conventional methods and PCR/electrospray ionization mass spectrometry. Such data are only included when the CC within the serotype is not represented by WGS-based data. For this reason, 13 of the 722 isolates are described as ‘not done’ for multiple WGS-based parameters. For these isolates, serotypes, CCs, partial or complete multilocus sequence types, resistance features and pilus detection were obtained solely with conventional (non-WGS-based) methods.

b Numbers of isolates within each serotype characterized are provided for each year, and abridged numbers are provided in parentheses when not all of the isolates of the indicated serotype were available. The ratios of isolates within indicated serotypes during the 3 years are only approximately proportional to the overall invasive pneumococcal disease incidence fluctuation of the serotype.

c Reference CC is chosen on the basis of the overall representation within multiple serotypes or the predominance within a specific serotype. Each CC has at least three non-identical alleles with other reference STs shown in the table. Individual STs within a CC must share at least four alleles with the reference ST.

d All STs designated 10142 or higher were initially found during this study.

e Indicated when the ST is related to the reference CC as an SLV, DLV, or TLV.

f The order of β-lactam R phenotypes is as follows: 1, penicillin; 2, amoxycillin; 3, meropenem; 4, cefotaxime/ceftriaxone (whichever is the highest); and 5, cefuroxime. The phenotype abbreviations and corresponding MIC values are provided in Materials and methods.

g The order of non-β-lactam R phenotypes is as follows: erythromycin, clindamycin, co-trimoxazole, tetracycline, and chloramphenicol. MIC ranges (mg/L) are described in Materials and methods.

h For serotype 19A/CC199, the various R phenotypes and causal determinant signatures shown in the last two columns are correlated by the use of identical symbols (!, +, *, #, or @). Phenotypes that are discordant with the determinant listing in column 10 are indicated in bold.

i The WGS-based serotype assignments for these Quellung-non-typeable isolates are as follows for the following genotypes: ST448, non-serotypeable; CC1292, serotype 6C; ST199, serotype 15B; ST1480, serotype 8.

## Appendix A Supplementary data

Table S1 Sequence coordinates used in the deduction of serotypes and pilus subunits from WGS analysis.

Table S2 Sequence coordinates and rationale for WGS-based antimicrobial resistance detection.

Table S3 PBP1a transpeptidase domain sequences compiled from approximately 1500 ABCs isolates collected during 1998–2013.

Table S4 PBP2b transpeptidase domain sequences compiled from approximately 1500 ABCs isolates collected during 1998–2013.

Table S5 PBP2x transpeptidase domain sequences compiled from approximately 1500 ABCs isolates collected during 1998–2013

Table S6 Identifiers for accession of fastq files provided by Sanger for this work at the European Nucleotide Archive (http://www.ebi.ac.uk/ena) and for fastq files provided by the CDC Biotechnology Core Facility at http://www.ncbi.nlm.nih.gov/genbank/genomessubmit

Doc. S1 Bioinformatic methods.

## References

[1] Whitney C.G., Farley M.M., Hadler J., Harrison L.H., Bennett N.M., Lynfield R. (2003). Decline in invasive pneumococcal disease after the introduction of protein–polysaccharide conjugate vaccine. N Engl J Med.

[2] Moore M.R., Link-Gelles R., Schaffner W., Lynfield R., Lexau C., Bennett N.M. (2015). Effect of use of 13-valent pneumococcal conjugate vaccine in children on invasive pneumococcal disease in children and adults in the USA: analysis of multisite, population-based surveillance. Lancet Infect Dis.

[3] Pilishvili T., Lexau C., Farley M.M., Hadler J., Harrison L.H., Bennett N.M. (2010). Sustained reductions in invasive pneumococcal disease in the era of conjugate vaccine. J Infect Dis.

[4] Wyres K.L., Lambertsen L.M., Croucher N.J., McGee L., von Gottberg A., Liñares J. (2013). Pneumococcal capsular switching: a historical perspective. J Infect Dis.

[5] Pai R., Moore M., Pilishvili T., Gertz R.E., Whitney C.G., Beall B. (2005). Post vaccine genetic structure of *Streptococcus pneumoniae* serotype 19A from children in the United States. J Infect Dis.

[6] Brueggemann A.B., Pai R., Crook D.W., Beall B. (2007). Vaccine escape recombinants emerge after pneumococcal vaccination in the United States. PLoS Pathog.

[7] Carvalho Mda G., Pimenta F.C., Gertz R.E., Joshi H.H., Trujillo A.A., Keys L.E. (2009). PCR-based quantitation and clonal diversity of the current prevalent invasive serogroup 6 pneumococcal serotype, 6C, in the United States in 1999 and 2006 to 2007. J Clin Microbiol.

[8] Beall B.W., Gertz R.E., Hulkower R.L., Whitney C.G., Moore M.R., Brueggemann A.B. (2011). Shifting genetic structure of invasive serotype 19A pneumococci in the United States. J Infect Dis.

[9] Massire C., Gertz R.E., Svoboda P., Levert K., Reed M.S., Pohl J. (2012). Concurrent serotyping and genotyping of pneumococci by use of PCR and electrospray ionization mass spectrometry. J Clin Microbiol.

[10] Gertz R.E., Li Z., Pimenta F.C., Jackson D., Juni B.A., Lynfield R. (2010). Increased penicillin-nonsusceptibility of nonvaccine serotype (other than 19A and 6A) invasive pneumococci in post 7 valent conjugate vaccine era. J Infect Dis.

[11] Zähner D., Gudlavalleti A., Stephens D.S. (2010). Increase in pilus islet 2-encoded pili among *Streptococcus pneumoniae* isolates, Atlanta, Georgia, USA. Emerg Infect Dis.

[12] Clinical and Laboratory Standards Institute (CLSI) (2013). Performance standards for antimicrobial susceptibility testing; Twenty-third informational supplement. CLSI document M100-S22.CLSI, Wayne.

[13] Cooper D., Yu X., Sidhu M., Nahm M.H., Fernsten P., Jansen K.U. (2011). The 13-valent pneumococcal conjugate vaccine (PCV13) elicits cross-functional opsonophagocytic killing responses in humans to *Streptococcus pneumoniae* serotypes 6C and 7A. Vaccine.

[14] Gertz R.E., McEllistrem M.C., Boxrud D.J., Li Z., Sakota V., Thompson T.A. (2003). Clonal distribution of invasive pneumococcal isolates from children and selected adults in the United States prior to 7-valent conjugate vaccine introduction. J Clin Microbiol.

[15] Contreras-Martel C., Dahout-Gonzalez C., Martins Ados S., Kotnik M., Dessen A. (2009). PBP active site flexibility as the key mechanism for beta-lactam resistance in pneumococci. J Mol Biol.

[16] van Selm S., van Cann L.M., Kolkman M.A., van der Zeijst B.A., van Putten J.P. (2003). Genetic basis for the structural difference between *Streptococcus pneumoniae* serotype 15B and 15C capsular polysaccharides. Infect Immun.

[17] Rajam G., Carlone G.M., Romero-Steiner S. (2007). Functional antibodies to the O-acetylated pneumococcal serotype 15B capsular polysaccharide have low cross-reactivities with serotype 15C. Clin Vaccine Immunol.

[18] Wyres K.L., Lambertsen L.M., Croucher N.J., McGee L., von Gottberg A., Liñares J. (2012). The multidrug-resistant PMEN1 pneumococcus is a paradigm for genetic success. Genome Biol.

[19] Beall B., McEllistrem M.C., Gertz R.E., Boxrud D.J., Besser J.M., Harrison L.H. (2002). Emergence of a novel penicillin-nonsusceptible, invasive serotype 35B clone of *Streptococcus pneumoniae* within the United States. J Infect Dis.

[20] Pai R., Gertz R.E., Whitney C.G., Beall B. (2005). Clonal association between *Streptococcus pneumoniae* serotype 23A, circulating within the United States, and an internationally dispersed clone of serotype 23F. J Clin Microbiol.

[21] Park I.H., Geno K.A., Sherwood L.K., Nahm M.H., Beall B. (2014). Population-based analysis of invasive nontypeable pneumococci reveals that most have defective capsule synthesis genes. PLoS One.

[22] Valentino M.D., McGuire A.M., Rosch J.W., Bispo P.J., Burnham C., Sanfilippo C.M. (2014). Unencapsulated *Streptococcus pneumoniae* from conjunctivitis encode variant traits and belong to a distinct phylogenetic cluster. Nat Commun.

[23] Golubchik T., Brueggemann A.B., Street T., Gertz R.E., Spencer C.C., Ho T. (2012). Pneumococcal genome sequencing tracks a vaccine escape variant formed through a multi-fragment recombination event. Nat Genet.

[24] Moore M.R., Gertz R.E., Woodbury R.L., Barkocy-Gallagher G.A., Schaffner W., Lexau C. (2008). Population snapshot of emergent *Streptococcus pneumoniae* serotype 19A in the United States, 2005. J Infect Dis.

[25] Beall B., McEllistrem M.C., Gertz R.E., Wedel S., Boxrud D.J., Gonzalez A.L. (2006). Pre- and postvaccination clonal compositions of invasive pneumococcal serotypes for isolates collected in the United States in 1999, 2001, and 2002. J Clin Microbiol.

[26] Contreras-Martel C., Job V., Di Guilmi A.M., Vernet T., Dideberg O., Dessen A. (2006, 27). Crystal structure of penicillin-binding protein 1a (PBP1a) reveals a mutational hotspot implicated in beta-lactam resistance in *Streptococcus pneumoniae*. J Mol Biol.

[27] Gordon E., Mouz N., Duée E., Dideberg O. (2000). The crystal structure of the penicillin-binding protein 2x from *Streptococcus pneumoniae* and its acyl-enzyme form: Implication in drug resistance. J Mol Biol.

[28] Park I.H., Moore M.R., Treanor J.J., Pelton S.I., Pilishvili T., Beall B. (2008). Differential effects of pneumococcal vaccines against serotypes 6A and 6C. J Infect Dis.

[29] Lee G.M., Kleinman K., Pelton S.I., Hanage W., Huang S.S., Lakoma M. (2014). Impact of 13-valent pneumococcal conjugate vaccination on *Streptococcus* pneumoniae carriage in young children in Massachusetts. J Pediatr Infect Dis Soc.

[30] Coffey T.J., Dowson C.G., Daniels M., Zhou J., Martin C., Spratt B.G. (1991). Horizontal transfer of multiple penicillin-binding protein genes, and capsular biosynthetic genes, in natural populations of *Streptococcus pneumoniae*. Mol Microbiol.

