# Supplementary material for: Strain features and distributions in pneumococci from children with invasive disease before and after 13-valent conjugate vaccine implementation in the USA
Source: Clin Microbiol Infect. 2016 Jan;22(1):60.e9–60.e29. doi: 10.1016/j.cmi.2015.08.027 (PMC4721534; doi:10.1016/j.cmi.2015.08.027)
Supplement: Table S1 — Sequence coordinates used in the deduction of serotypes and pilus subunits from WGS analysis. [file mmc1.docx]

sTable 1.

| Serotype^A^ | required 1 (no. bp)^B^ | GenBank:  Coordinates 1 | required 2 ^B^ (no. bp) | GenBank  Coordinates 2 |
| --- | --- | --- | --- | --- |
| 1 | *wzy1*-1 (100) | CR931632:9704-9803 |  |  |
| 2 | *wzy2*-1 (100) | CR931633:9869-9968 |  |  |
| 3 | *wchE3*-1 (50) | CR931634:7502-7151 |  |  |
| 4 | *wzy4*-1 (100) | CR931635:9537-9636 |  |  |
| 5 | *wzy5*-1 (100) | CR931637:6103-6202 |  |  |
| 6A | any of these 4: *wciP*6AC-1 (41)*  *wciP6AC*-2 (41)*  *wciP6AC*-3 (41)*  *wciP6AC*-4 (41)* | CR931638:8871-8911  KF651412:399-439  AB795221:3579-3619  KF597302:2866-2906 | *wciN*6AB-1 (101) | CR931638:6465-6565 |
| 6C | any of these 4: *wciP6AC-1* (41)*  *wciP6AC-2* (41)*  *wciP6AC-3* (41)*  *wciP6AC-4* (41)* | CR931638:8871-8911  KF651412:399-439  AB795221:3579-3619  KF597302:2866-2906 | *wciN*6CD-2 (100) | AB795236:997-1096 |
| 6B | *wciP*6BD-1(41)* | CR931639:8869-8909 | *wciN*6AB-1new (101) | CR931638:6465-6565 |
| 6D | *wciP*6BD-1 (41)* | CR931639:8869-8909 | *wciN*6CD -2 (100) | AB795236:997-1096 |
| 7C | *wzy*7C-1 (99) | CR931642:11978-12076 | *wchF*7C-1 (77) | CR931642:7020-7096 |
| 7B | *wzy*7C-1 (99) | CR931642:11978-12072 |  |  |
| 7A | *wzy*7F-1 (100) | CR931643:14465-14564 | *wcwD*7F (inactive) | CR931643:9565-10527 |
| 7F | *wzy*7F-1 (100) | CR931643:14465-14564 | active *wcwD*7F (963)* | CR931643:9565-10527  wcw7D A25V? |
| 8 | *wzy8-*1 (100) | CR931644:10954-11053 |  |  |
| 9L | *wzy*9N-1 (100) | CR931647:10746-10845 | *wcjA*9L-1 (40)* | CR931648:7948-7987 |
| **9N** | *wzy*9N-1 (100) | CR931647:10746-10845 | *wcjA*9N-1 (40)* |  |
| 9A | *wzy*9V-1 (100) | CR931648:10746-10845 | *wcjE*-9V (inactive) | CR931648:18127-19161 |
| 9V | *wzy*9V-1 (100) | CR931648:10746-10845 | active *wcjE*-9V (1035)* | CR931648:18127-19161 |
| 10A | *wcrG*10A-1 (80) | CR931649:12487-12566 |  |  |
| 10F | *gtf*10F-1 (99) | CR931652:8891-8989 |  |  |
| 11A/11D | *wzy*11A-1 (83) | CR931656:11308-11390 |  |  |
| 11B/11C | *wzy*11B-1 (100) | CR931655:12534-12633 |  |  |
| 12A/12B/46 | *wciI*12B-1 (129) | CR931659:5209-5337 |  |  |
| 12F/44 | *wciI*12F-1 (129) | CR931660:5210-5338 |  |  |
| 13 | *wzy13*-1 (100) | CR931661:13470-13569 |  |  |
| 14 | *wzy14*-1 (98) | CR931662:7745-7842 |  |  |
| 15A | *wzy15A*-1 (79) | CR931663:7536-7614 |  |  |
| 15F | *wzy15A*-1 (79) | CR931663:7536-7614 | *wciZ*15F-1 (50)* | CR931666:13804-13853 |
| 15B | *wzy*15B-1 (100) | CR931664:7740-7839 | wciZ15B-1 (50)* | CR931664:13419-13468 |
| 15C | *wzy*15B-1 (100) | CR931664:7740-7839 | *wciZ*15B-1 (altered) | CR931664:13419-13468 |
| 16F | *wzy16F*-1 (100) | CR931668:11270-11369 |  |  |
| 17F | *gtf*17F-1 (100) | CR931670:10620-10719 |  |  |
| 18A | *wzy*18A-1 (397)* | CR931671:12779-13175 |  |  |
| 18C | *wzy*18C-1 (397)* | CR931673:12679-13075 | wciX18C (1002)* | CR931673:13416-14417 |
| 18B | wzy18C-1 (397)* | CR931673:12679-13075 | wciX18C (1002) inactive |  |
| 18F | *wzy*18F-1 (397)* | CR931674:13720-14116 |  |  |
| 19A | One of: *wzy*19A-1 (132)*  *wzy*19Avar-1 (104)* | AF094575:9659-9790  JX112901:171-274 |  |  |
| 19F | One of: *wzy19F*-1 (104)*  *wzy19F*-2 (104)*  *wzy19Fvar*-1 (131)*  *wzy19Fvar* -2 (103)* | CR931678:11050-11153  JF911531:7575-7678  KC690152:522-651  J829071:522-651 |  |  |
| 20 | *wzy20*-1 (100) | CR931679:7392-7491 |  |  |
| 21 | *wzy21*-1 (100) | CR931680:12051-12150 |  |  |
| 22A | *wzy*22F-1 (100) | CR931682:12961-13060 | *wcwA*22A (100) | CR931682:8690-8789 |
| 22F | *wzy*22F-1 (100) | CR931682:12961-13060 | *wcwA*22F (100) | HE651300:39-138 |
| 23A | *wzy*23A-1 (100) | CR931683:8218-8317 |  |  |
| 23B | *wzy*23B-1 (93) | CR931684: 9696-9788 |  |  |
| 23F | *wzy*23F-1 (100) | CR931685:9197-9296 |  |  |
| 24F/24A/24B | *wzy*24F-1 (100) | CR931688:12320-12415 |  |  |
| 25F/25A | *wcyE*25F-1 (100) | CR931690 :16530-16629 |  |  |
| 28A | wzy28A-1 (100) | CR931692:10662-10761 |  |  |
| 31 | *wzy31*-1 (100) | CR931695:9426-9525 |  |  |
| 33A | *wzy33F*-1 (100) | CR931702:11392-11491 | wcjE33A-1* | CR931698:16779-16838 |
| 33F | *wzy33F*-1 (100) | CR931702:11392-11491 |  |  |
| 37 | *tts-1* (80) | AJ131985:2211-2290 |  |  |
| 34 | *wzy*34-1 (100) | CR931703:7750-7849 |  |  |
| 35A | *wzy*35A-1 (35) | CR931704:7400-7434 |  |  |
| 35B | *wzy*35B-1 (100) | CR931705:8123-8222 |  |  |
| 35C/42 | *wzy*35C-1 (35) | CR931706:7738-7772 |  |  |
| 35F | *wcrO*35F-1 (51) | CR931707:8254-8304 |  |  |
| 47F | *whaI*47F-1 (51) | CR931721:7965-8015 |  |  |
| 38 | *wcyV*38-1 (100) | CR931710:18264-18363 |  |  |
| PI-1 | *rrgA-1* (100) | CP000921:463577-463676 |  |  |
| PI-2 | *pitB-1* (100) | CP000921:1003530-1003568 |  |  |

^A^ Shaded serotype sets 6A/6C, 6B/6D, 7C/7B, 7A/7F, 9L/9N, 9A/9V, 15A/15F, 15B/15C, and 33A/33F/37 are not resolved by the sequence query (column 1 and 2) but require a second sequence query (column 3 and 4). Six sets of 2-3 serotypes shown as non-shaded entries in column 1 are co-identified by this scheme. The sequences *rrgA*-1 and *pitB*-1 are also included here and are used for detection of the two different pilus subunits associated with PI-1 and PI-2, respectively.

^B^Entries with asterisk indicate complete identity to the indicated sequences (described in columns 2-5) required. For all other entries, > 95% sequence identity to the indicated sequence required. For many serotypes only one identifying sequence was utilized (column 2 provides gene name, column 3 provides GenBank accession coordinates). Certain other serotypes employed a second target for resolving highly related serotypes (columns 4 and 5). Highly related pairs of serotypes that are resolved from each other are shaded together.
