# Supplementary material for: Strain features and distributions in pneumococci from children with invasive disease before and after 13-valent conjugate vaccine implementation in the USA
Source: Clin Microbiol Infect. 2016 Jan;22(1):60.e9–60.e29. doi: 10.1016/j.cmi.2015.08.027 (PMC4721534; doi:10.1016/j.cmi.2015.08.027)
Supplement: Table S2 — Sequence coordinates and rationale for WGS-based antimicrobial resistance detection. [file mmc2.docx]

sTable 2. WGS-based antimicrobial resistance detection platform base coordinates and rationale.

| query (No. bp) | resistance | Accession, sequence coordinates | rationale |
| --- | --- | --- | --- |
| *pbp1a* (831)  *pbp2b* (834)  *pbp2x* (1077) | various classes of beta lactam antibiotics | 1a: AE007317, 333083 -333913 (complement)  2b: AE007317, 1494292-1495124 (complement)  2x: AE007317, 302945-304019 | specific combinations of the 3 PBP transpeptidase domains predict  different minimum inhibitory concentrations to specific beta lactams |
| *ermB* (100)  *ermTR* (68) | macrolides, lincosamides  streptogramins | HG799494,44520-44619  CP002121,856516-856583 | ≥ 95% sequence identity predicts presence predicts resistance |
| 23SWT1-1 (34)^A^  23SWT2-1 (34)^A^ | macrolides, lincosamides  streptogramins | AE007317,19182-19215  AE007317,19741-19770   - these coordinates depict only 1 of 4 copies of 23S rRNA gene | Specific base substitutions (rare) confer resistance. Analyze and  document phenotypes for all encountered substitutions.  A2060C, A2061G, C2613G predict erythromycin + clindamycin resistance |
| *rplD-1* (36) ^A^  *rplD*-2 (30) ^A^  *rplV****-1*** (27) ^A^  *rplV****-2*** (30) ^A^ | macrolides, streptogramins | *rplD-1*: AE007317,196913-196948  *rplD*-2: AE007317,196787-196839  *rplV-1*: AE007317,199197-199223  *rplV-2* : AE007317,199143-199172 | Specific translational changes (rare) predict resistance. Analyze and  document phenotypes for all encountered variants. |
| *ermB* leader (69)^B^ | telithromycin | HG799494,43866-43926 | Alterations in *ermB* leader sequence can cause high level  MLS resistance, and rare variants confer telithromycin-resistance |
| *mefA* (67) | macrolides | CP000921,2802680-1802746 | ≥ 95% sequence identity predicts resistance |
| *folA* (60) | trimethoprim resistance, intermediate cotrimoxazole-resistance | AE007317,1413068-1413127 | I100L substitutions (common) and D92R (not yet seen in ABCs isolates) confer trimethoprim resistance; I100L substitution alone confers intermediate cotrimoxazole-resistance. Usually seen in combination with *folP* I100L with full cotrimoxaxole resistance (> 4 µg/ml). |
| FOLP-1(57) | sulfamethoxazole resistance, intermediate cotrimoxazole resistance | AE007317,268022-268300 | various insertions of 1-2 codons between bases 168 and 201 of *folP* confer sulfamethoxazole resistance and intermediate cotrimoxazole-resistance |
| *folA-1* + *folP-1*^c^ | cotrimoxaxole  (trimethoprim+  sulfamethoxaxole) resistance |  | combination of two above features in *folA* and *folP* predicts full resistance to cotrimoxazole. |
| ***gyrA*** (78) | fluoroquinolones | AE007317,1097644-1097721 | S81Y or S81F predicts resistance^d^ |
| *parC* (210) | fluoroquinolones | AE007317,752481-752690 | each of S79F, S79Y, D83N, D83Y, N91D, N91N predict resistance^D^ |
| *gyrA-1*+*parC-1* | fluoroquinolones |  | combinations of the above dictate different resistance levels |
| TETM-1(100)  TETO-1(100) | tetracycline resistance | HG799494,42545-42644  FM178797,1754-1853 (complement) | ≥ 95% sequence identity predicts presence predicts resistance |
| CAT-1 (100) | chloramphenicol resistance | HG799499,56681-56780 | ≥ 95% sequence identity predicts presence predicts resistance |
| RPOB-1 (99) ^E^ | rifampin resistance | AE007317,1868571-1868473 | Seven known substitutions predict resistance; any translational change requires phenotype analysis |
| *vanA-1* (120)  *vanB-1* (120)  *vanC-1* (120)  *vanD-1* (120)  *vanE-1* (117)  *vanG-1* (120) | vancomycin resistance | KM235680,495-614  KC489787,5466-5585  JX435778,73-192  EU999036,4326-4442  J872411,40045-40155  KF704242,43286-43346 | ≥ 85% sequence identity to any of these peptidoglycan precursor altering determinants  potentially predictive of vancomycin-resistance which has not been documented  in *S. pneumoniae*. |

**^A^** The contributions of several specific amino acid and rRNA substitutions within translational apparatus known, however new associations still being compiled

^B^Although certain alterations are known that predict constitutive lincosamide and high level macrolide resistance (and in certain cases telithromycin-resistance), the mechanism is still under investigation (see Hawkins PA, Chochua S, Jackson D, Beall B, McGee L. Mobile Elements and Chromosomal Changes Associated with MLS Resistance Phenotypes of Invasive Pneumococci Recovered in the United States. Microb Drug Resist. 2015 In Press). Less than 95% identity dictates performance of specific MIC testing for telithromycin.

^C^ Cornick JE, Harris SR, Parry CM et al. Genomic identification of a novel co-trimoxazole resistance genotype and its prevalence amongst Streptococcus pneumoniae in Malawi. J Antimicrob Chemother. 2014; 69 :368-74

^D^ Brueggemann AB, Coffman SL, Rhomberg P, et al. Fluoroquinolone resistance in Streptococcus pneumoniae in United States since 1994-1995. Antimicrob Agents Chemother. 2002; 46: 680-8.

^E^ Ferrándiz MJ, Ardanuy C, Liñares J, Balsalobre L, García MT, de la Campa AG. New species genetic approach to identify strains of mitis group streptococci that are donors of rifampin resistance to Streptococcus pneumoniae. Antimicrob Agents Chemother. 2011; 55: 368-72.
