# Supplementary material for: Strain features and distributions in pneumococci from children with invasive disease before and after 13-valent conjugate vaccine implementation in the USA
Source: Clin Microbiol Infect. 2016 Jan;22(1):60.e9–60.e29. doi: 10.1016/j.cmi.2015.08.027 (PMC4721534; doi:10.1016/j.cmi.2015.08.027)
Supplement: Table S3 — PBP1a transpeptidase domain sequences compiled from approximately 1500 ABCs isolates collected during 1998–2013. [file mmc3.docx]

sTable 3. PBP1a Transpeptidase domain sequences compiled from approximately 1500 ABCs isolates collected during 1998-2013.

>0

TMKPITDYAPALEYGVYDSTATIVHDEPYNYPGTNTPVYNWDRGYFGNITLQYALQQSRNVPAVETLNKVGLNRAKTFLNGLGIDYPSIHYSNAISSNTTESDKKYGASSEKMAAAYAAFANGGTYYKPMYIHKVVFSDGSEKEFSNVGTRAMKETTAYMMTDMMKTVLTYGTGRNAYLAWLPQAGKTGTSNYTDEEIENHIKTSQFVAPDELFAGYTRKYSMAVWTGYSNRLTPLVGNGLTVAAKVYRSMMTYLSEGSNPEDWNIPEGLYRNGEFV

>1

TMKPITDYAPALEYGIYDSTATIVHDEPYNYPGTNTPVYNWDRGYFGNITLQYALQQSRNVPAVETLNKVGLNRAKTFLNGLGIDYPSIHYSNAISSNTTESDKKYGASSEKMAAAYAAFANGGTYYKPMYIHKVVFSDGSEKEFSNVGTRAMKETTAYMMTDMMKTVLSYGTGRNAYLAWLPQAGKTGTSNYTDEEIENHIKTSQFVAPDELFAGYTRKYSMAVWTGYSNRLTPLVGNGLTVAAKVYRSMMTYLSEGSNPEDWNIPEGLYRNGEFV

>2

TMKPITDYAPALEYGVYDSTATIVHDEPYNYPGTNTPVYNWDRGYFGNITLQYALQQSRNVPAVETLNKVGLNRAKTFLNGLGIDYPSIHYSNAISSNTTESDKKYGASSEKMAAAYAAFANGGTYYKPMYIHKVVFSDGSEKEFSNVGTRAMKETTAYMMTDMMKTVLSYGTGRNAYLAWLPQAGKTGTSNYTDEEIENHIKTSQFVAPDELFAGYTRKYSMAVWTGYSNRLTPLVGNGLTVAAKVYRSMMTYLSEGSNPEDWNIPEGLYRNGEFV

>3

TMKPITDYAPALEYGVYDSTAAIVHDEPYNYPGTNTPVYNWDRGYFGNITLQYALQQSRNVPAVETLNKVGLNRAKTFLNGLGIDYPSIHYSNAISSNTTESDKKYGASSEKMAAAYAAFANGGTYYKPMYIHKVVFSDGSEKEFSNVGTRAMKETTAYMMTDMMKTVLTYGTGRNAYLAWLPQAGKTGTSNYTDEEIENHIKTSQFVAPDELFAGYTRKYSMAVWTGYSNRLTPLVGNGLTVAAKVYRSMMTYLSEGSNPEDWNIPEGLYRNGEFV

>4

SMKPITDYAPALEYGVYDSTATMVNDIPYNYPGTSTPVYNWDRAYFGNISLQYALQQSRNVPAVETLNKVGLDRAKTFLNGLGIDYPSIHYSNAISSNTTESSKQYGASSEKMAAAYAAFANGGIYHKPMYINKVVFSDGSEKEFSDAGTRAMKETTAYMMTEMMKTVLTYGTGRGAYLPWLPQAGKTGTSNYTDDEIEKYIKNTGYVAPDEMFVGYTRKYSMAVWTGYSNRLTPIIGDGFLVAAKVYRSMISYLSEDDHPGDWTMPEGVYRSGEFV

>5

TMKPITDYAPALEYGVYDSTATIVHDEPYNYPGTNTPVYNWDRGYFGNITLQYALQQSRNVPAVETLNKVRLNRAKTFLNGLGIDYPSIHYSNAISSNTTESDKKYGASSEKMAAAYAAFANGGTYYKPMYIHKVVFSDGSEKEFSNVGTRAMKETTAYMMTDMMKTVLSYGTGRNAYLAWLPQAGKTGTSNYTDEEIENHIKTSQFVAPDELFAGYTRKYSMAVWTGYSNRLTPLVGNGLTVAAKVYRSMMTYLSEGSNPEDWNIPEGLYRNGEFV

>6

TMKPITDYAPALEYGVYDSTATIVHDEPYNYPGTDTPVYNWDRGYFGNITLQYALQQSRNVPAVETLNKVGLNRAKTFLNGLGIDYPSLHYSNAISSNTTESDKKYGASSEKMAAAYAAFANGGTYYKPMYIHKVVFSDGSEKEFSNVGTRAMKETTAYMMTDMMKTVLVYGIGRGAYLPWLPQAGKTGTSNYTDEEIEKYIKNTGYVAPDELFAGYTRKYSMAVWTGYSNRLTPLVGDGLTVAAKVYRSMMTYLSEGSNPEDWNIPEGLYRNGEFV

>7

TMKPITDYAPAIEYGVYDSTATMVNDIPYNYPGTSTPVYNWDRAYFGNITLQYALQQSRNVTAVETLNKVGLDRAKTFLNGLGIDYPSIHYANAISSNTTESNKHYGASSEKMAAAYAAFANGGIYHKPMYINKIVFSDGSEKEFSDAGTRAMKETTAYMMTEMMKTVLAYGTGRGAYLPWLPQAGKTGTSNYTDDEIEKHIKNTGYVAPDEMFVGYTRKYSMAVWTGYSNRLTPIVGDGFLVAAKVYRSMISYLSEDDQPGDWTMPDGLFRNGEFV

>8

TMKPITDYAPALEYGVYDSTATIVHDEPYNYPGTDTPVYNWDRGYFGNITLQYALQQSRNVPAVETLNKVGLNRAKTFLNGLGIDYPSLHYSNAISSNTTESDKKYGASSEKMAAAYAAFANGGTYYKPMYIHKVVFSDGSEKEFSNVGTRAMKETTAYMMTDMMKTVLVYGIGRGAYLPWLPQAGKTGTSNYTDEEIEKYIKNTGYVAPDEMFVGYTRKYAMAVWTGYSNRLTPLVGDGLTVAAKVYRSMMTYLSEGSNPEDWNIPEGLYRNGEFV

>9

TMKPITDYAPALEYGVYDSTATIVHDEPYNYPGTNTPVYNWDRGYFGNITLQYALQQSRNVPAVETLNKVGLNRAKTFLNGLGIDYPSIHYSNAISSNTTESDKKYGASSEKMAAAYAAFANGGTYYKPMYIHKVVFSDGSEKEFSNVGTRVMKETTAYMMTDMMKTVLTYGTGRNAYLAWLPQAGKTGTSNYTDEEIENHIKTSQFVAPDELFAGYTRKYSMAVWTGYSNRLTPLVGNGLTVAAKVYRSMMTYLSEGSNPEDWNIPEGLYRNGEFV

>10

TMKSITDYAPALEYGVYDSTATIVHDVPYNYPGTDTPVYNWDKGYFGNITIQYALQQSRNVTAVETLNKVGLDRAKTFLNGLGIDYPSIHYANAISSNTTESNKQYGASSEKMAAAYAAFANGGIYHKPMYINKVVFSDGSEKEFSDAGTRAMKETTAYMMTEMMKTVLAYGTGRGAYLPWLAQAGKTGTSNYTDDEIEKHIKNTGYVAPDETFVGYTRKYSMAVWTGYSNRLTPIVGDGFLVAAKVYRSMITYLSEDTHPEDWTMPDGLFRNGEFV

>11

TMKPITDYAPALEYGVYDSTATIVHDEPYNYPGTNTPVYNWDRGYFGNITLQYALQQSRNVPAVETLNKVGLNRAKTFLNGLGIDYPSIHYSNAISSNTTESDKKYGASSEKMAAAYAAFANGGTYYKPMYIHKVVFSDGSEKEFSNVGTRAMKETTAYMMTDMMKTVLTYGTGRNAYLAWLPQAGKTGTSNYTDEEIENHIKTSQFVAPDELFAGYTRKYSMAVWTGYSNRLTPLVGNGLTVAAKVYRSMMTYLSEGRNPEDWNIPEGLYRNGEFV

>12

TMKPITDYAPALEYGVYDSTATIVHDEPYNYPGTNTPVYNWDRGYFGNITLQYALQQSRNVPAVETLNKVGLNRAKTFLNGLGIDYPSIHYSNAISSNTTESDKKYGASSEKMAAAYAAFANGGTYYKPMYIHKVVFSDGSEKEFSNVGTRAMKETTAYMMTEMMKTVLSYGTGRNAYLAWLPQAGKTGTSNYTDEEIENHIKTSQFVAPDELFAGYTRKYSMAVWTGYSNRLTPLVGNGLTVAAKVYRSMMTYLSEGSNPEDWNIPEGLYRNGEFV

>13

SMKPITDYAPALEYGVYDSTASIVHDVPYNYPGTDTPLYNWDHVYFGNITIQYALQQSRNVTAVETLNKVGLDRAKTFLNGLGIDYPSMHYANAISSNTTESNKKYGASSEKMAAAYAAFANGGIYHKPMYINKIVFSDGSEKEFSDAGTRAMKETTAYMMTEMMKTVLTYGTGRGAYLPWLPQAGKTGTSNYTDEEIEKYIKNTGYVAPDEMFVGYTRKYSMAVWTGYSNRLTPIVGDGFLVAAKVYRSMMTYLSEGSNPEDWNIPEGLYRNGEFV

>14

TMKPITDYAPALEYGVYDSTATIVHDEPYNYPGTNTPVYNWDRGYFGNITLQYALQQSRNVPAVETLNKVGLNRAKTFLNGLGIDYPSIHYSNAISSNTTESDKKYGASSEKMAAAYAAFANGGTYYKPMYIHKVVFSDGSEKEFSNVGTRAMKETTAYMMTDMMKTVLSYGTGRNAYLAWLPQAGKTGTSNYTDEEIENHIKTSQFVAPDELFAGYTRKYSMAVWTGYSNRLTPLVGNGLTVAAKVYRSMMTYLFEGSNPEDWNIPEGLYRNGEFV

>15

AMKPITDYAPAIEYGVYDSTATMVNDIPYNYPGTSTPVYNWDRAYFGNITLQYALQQSRNVTAVETLNKVGLDRAKTFLNGLGIDYPSMHYANAISSNTTESNKQYGASSEKMAAAYAAFANGGTYYKPMYIHKVVFSDGSKKEFSNVGTRAMKETTAYMMTDMMKTVLTYGTGRGAYLPWLPQAGKTGTSNYTDEEVENHIKNTGYVAPDEMFVGYTRKYSMAVWTGYSNRLTPIVGDGFLVAAKVYRSMITYLSEDTHPEDWTMPDGLFRNGEFV

>16

TMKPITDYAPALEYGVYDSTATIVHDEPYNYPGTDIPVYNWDRGYFGNITLQYALQQSRNVPAVETLNKVGLNRAKTFLNGLGIDYPSIHYSNAISSNTTESDKKYGASSEKMATAYAAFANGGTYYKPMYIHKVVFSDGSEKEFSNVGTRAMKETTAYMMTEMMKTVLTYGIGRGAYLPWLPQAGKTGTSNYTDEEIEKYIKNTGYVAPDEMFVGYTRKYAMAVWTGYSNRLTPIMGDGLTVAAKVYRAMITYLSEGSNPEDWNIPEGLYRNGEFV

>17

AMKPITDYAPALEYDIYDSTASIVHDVPYNYPGTDTPLYNWDKVYFGNITIQYALQQSRNVTAVETLNKVGLDRAKTFLNGLGIDYPSMHYANAISSNTTESNKKYGASSEKMAAAYAAFANGGIYHKPMYINKIVFSDGSSKEYADPGTRAMKETTAYMMTEMMKTVLAYGTGRGAYLPWLPQAGKTGTSNYTDDEIENYIKNTGYVAPDEMFVGYTRKYSMAVWTGYSNRLTPIVGDGFYVAAKVYRSMMTYLSEDNNPGDWTMPEGLYRSGEFV

>18

SMKPITDYAPALEYGVYDSTASIVHDVPYNYPGTDTPLYNWDHVYFGNITIQYALQQSRNVTAVETLNKVGLDRAKTFLNGLGIDYPSMHYANAISSNTTESNKKYGASSEKMAAAYAAFANGGIYHKPMYINKVVFSDGSEKEFSDVGTRAMKETTAYMMTEMMKTVLAYGTGRGAYLPWLAQAGKTGTSNYTDDEIEKHIKNTGYVAPDEMFVGYTRKYSMAVWTGYSNRLTPIVGDGFLVAAKVYRSMITYLSEDTHPEDWTMPDGLFRNGEFV

>19

TMKPITDYAPALEYGVYDSTATIVHDEPYNYPGTDTPVYNWDRGYFGNITLQYALQQSRNVPAVETLNKVGLNRAKTFLNGLGIDYPSLHYSNAISSNTTESDKKYGASSEKMAAAYAAFANGGTYYKPMYIHKVVFSDGSEKEFSNVGTRAMKETTAYMMTDMMKTVLVYGIGRGAYLPWLPQAGKTGTSNYTDEEIEKYIKNTGYVAPDEMFVGYTRKYAMAVWTGYSNRLTPLVGDGLTVAAKVYRSMMTYLSEGSNPEDWNMPEGLYRNGEFV

>20

TMKPITDYAPALEYGVYDSTATIVHDEPYNYPGTDIPVYNWDRGYFGNITLQYALQQSRNVPAVETLNKVGLNRAKTFLNGLGIDYPSLHYSNAISSNTTESNKQYGASSEKMAVAYAAFANGGIYHKPMYINKVVFSDGSEKEFSDAGTRAMKETTAYMMTEMMKTVLVYGIGRGAYLPWLPQAGKTGTSNYTDEEIEKYIKNTGYVAPDEMFVGYTRKYAMAVWTGYSNRLTPLVGDGLTVAAKVYRSMMTYLSEGSNPEDWNMPEGLYRNGEFV

>21

TMKPITDYAPALEYGVYDSTATIVHDEPYNYPGTNTPVYNWDRGYFDNITLQYALQQSRNVPAVETLNKVGLNRAKTFLNGLGIDYPSIHYSNAISSNTTESDKKYGASSEKMAAAYAAFANGGTYYKPMYIHKVVFSDGSEKEFSNVGTRAMKETTAYMMTDMMKTVLTYGTGRNAYLAWLPQAGKTGTSNYTDEEIENHIKTSQFVAPDELFAGYTRKYSMAVWTGYSNRLTPLVGNGLTVAAKVYRSMMTYLSEGSNPEDWNIPEGLYRNGEFV

>22

TMKPITDYAPALEYGVYDSTATIVHDEPYNYPGTNTPVYNWDRGYFGNITLQYALQQSRNVPAVETLNKVGLIRAKTFLNGLGIDYPSIHYSNAISSNTTESDKKYGASSEKMAAAYAAFANGGTYYKPMYIHKVVFSDGSEKEFSNVGTRAMKETTAYMMTDMMKTVLSYGTGRNAYLAWLPQAGKTGTSNYTDEEIENHIKTSQFVAPDELFAGYTRKYSMAVWTGYSNRLTPLVGNGLTVAAKVYRSMMTYLSEGSNPEDWNIPEGLYRNGEFV

>23

TMKPITDYAPALEYGIYDSTATIVHDEPYNYPGTNTPVYNWDRGYFGNITLQYALQQSRNVPAVETLNKVGLNRAKTFLNGLGIDYPSIHYSNAISSNTTESDKKYGASSEKMAAAYAAFANGGTYYKPMYIHKVVFSDGSEKEFSNVGTRAMKETTAYMMTDMMKTVLTYGTGRNAYLAWLPQAGKTGTSNYTDEEIENHIKTSQFVAPDELFAGYTRKYSMAVWTGYSNRLTPLVGNGLTVAAKVYRSMMTYLSEGSNPEDWNIPEGLYRNGEFV

>24

TMKPITDYAPAIEYGVYDSTATMVNDIPYNYPGTSTPVYNWDRAYFGNITLQYALQQSRNVPAVETLNKVGLDRAKNFLNGLGIDYPDMHYSNAISSNTTESNKQYGASSEKMAAAFAAFANGGIYHKPMYINKIVFSDGSEKEFSDAGTRAMKETTAYMMTEMMKTVLSYGTGRNAYLAWLPQAGKTGTSNYTDEEIENHIKTSQFVAPDELFAGYTRKYSMAVWTGYSNRLTPLVGNGLTVAAKVYRSMMTYLSEGSNPEDWNIPEGLYRNGEFV

>25

AMKPITDYAPAIEYGVYDSTATMVNDIPYNYPGTSTPVYNWDRAYFGNITLQYALQQSRNVTAVETLNKVGLDRAKTFLNGLGIDYPSMHYANAISSNTTESNKQYGASSEKMAAAYAAFANGGIYHKPMYINKVVFSDGSEKEFSDVGTRAMKETTAYMMTEMMKTVLAYGTGRGAYLPWLAQAGKTGTSNYTDDEIEKHIKNTGYVAPDEMFVGYTRKYSMAVWTGYSNRLTPIVGDGFLVAAKVYRSMITYLSEDTHPEDWTMPDGLFRNGEFV

>26

TMKPITDYAPALEYGVYDSTATIVHDEPYNYPGTNTPVYNWDRGYFGNITLQYALQQSRNVPAVETLNKVGLNRAKTFLNGLGIDYPSIHYSNAISSNTTESDKKYGASSEKMATAYAAFANGGIYHKPMYINKVVFSDGSEKEFSDAGTRAMKETTAYMMTEMMKTVLAYGIGRGAYLPWLPQAGKTGTSNYTDDEIEKYIKNTGYVAPDEMFVGYTRKYSMAVWTGYTNRLTPIMGDGLTVAAKVYRSMMTYLSEGSNPEDWNIPEGLYRNGEFV

>27

SMKPITDYAPALEYGVYDSTASIVHDVPYNYPGTDTPLYNWDHVYFGNITIQYALQQSRNVTAVETLNKVGLDRAKTFLNGLGIDYPSMHYANAISSNTTESNKKYGASSEKMAAAYAAFANGGIYHKPMYINKIVFSDGSEKEFSDAGTRAMKETTAYMMTEMMKTVLTYGTGRGAYLPWLPQAGKTGTSNYTDEEIEKYIKNTGYVAPDEMFVGYTRKYAMAVWTGYSNRLTPIIGDGFLVAGKVYRSMITYLSEDDQPGDWTMPDGLYRNGEFV

>28

SMKPITDYAPALEYGVYDSTATMVNDIPYNYPGTSTPVYNWDRAYFGNITLQYALQQSRNVTAVETLNKVGLDRAKTFLNGLGIDYPSMHYANAISSNTTESDKKYGASSEKMAAAYAAFANGGIYHKPMYINKIVFSDGSEKEFSDAGTRAMKETTAYMMTEMMKTVLVYGIGRGAYLPWLPQAGKTGTSNYTDEEIEKYVKNTGYVAPDESFVGYTRKYAMAVWTGYSNRLTPLVGDGLTVAAKVYRSMMTYLSEGSNPEDWNIPEGLYRNGEFV

>29

SMKPITDYAPALEYGVYDSTASIVHDVPYNYPGTDTPLYNWDHGYFGNITIQYALQQSRNVTAVETLNKVGLDRAKTFLNGLGIDYPSMHYANAISSNTTESNKQYGASSEKMAAAYAAFANGGIYHKPMYINKIVFSDGSEKEFSDAGTRAMKETTAYMMTEMMKTVLVYGIGRGAYLPWLPQAGKTGTSNYTDEEIEKYVKNTGYVAPDESFVGYTRKYAMAVWTGYSNRFTPIVGDGFLVAAKVYRSMMTYLSEGSNPEDWNIPEGLYRNGEFV

>30

TMKPITDYAPALEYGVYDSTATIVHDEPYNYPGTNTPIYNWDRGYFGNITLQYALQQSRNVPAVETLNKVGLNRAKTFLNGLGIDYPSIHYSNAISSNTTESDKKYGASSEKMAAAYAAFANGGTYYKPMYIHKVVFSDGSEKEFSNVGTRAMKETTAYMMTDMMKTVLTYGTGRNAYLAWLPQAGKTGTSNYTDEEIENHIKTSQFVAPDELFAGYTRKYSMAVWTGYSNRLTPLVGNGLTVAAKVYRSMMTYLSEGSNPEDWNIPEGLYRNGEFV

>31

AMKPITDYAPAIEYGVYDSTATMVNDIPYNYPGTSTPVYNWDRAYFGNITLQYALQQSRNVTAVETLNKVGLDRAKTFLNGLGIDYPSMHYANAISSNTTESNKQYGASSEKMAAAYAAFANGGTYYKPMYIHKVVFSDGSKKEFSNVGTRAMKETTAYMMTDMMKTVLTYGTGRGAYLPWLPQAGKTGTSNYTDEEVENHIKNTGYVAPDEMFVGYTRKYSMAVWTGYSNRLTPIVGDGFLVAAKVYRSMMTYLSEGSNPEDWNIPEGLYRNGEFV

>32

TMKPITDYAPALEYGVYDSTATIVHDEPYNYPGTNTPVYNWDRGYFGNITLQYALQQSRNVPAVETLNKVGLNRAKTFLNGLGIDYPSIHYSNAISSNTTESDKKYGASSEKMAAAYAAFANGGTYYKPMYIHKVVFSDGSEKEFSNVGTRAMKETTAYMMTDMMKTVLTYGTGRNSYLAWLPQAGKTGTSNYTDEEIENHIKTSQFVAPDELFAGYTRKYSMAVWTGYSNRLTPLVGNGLTVAAKVYRSMMTYLSEGSNPEDWNIPEGLYRNGEFV

>33

TMKPITDYAPALEYGVYDSTATIVHDEPYNYPGTNTPVYNWDRGYFGNITLQYALQQSRNVPAVETLNKVGLNRAKTFLNGLGIDYPSLHYSNAISSNTTESDKKYGASSEKMAAAYAAFANGGTYYKPMYIHKVVFSDGSEKEFSNVGTRAMKETTAYMMTDMMKTVLVYGIGRGAYLPWLPQAGKTGTSNYTDEEIEKYIKNTGYVAPDELFAGYTRKYSMAVWTGYSNRLTPLVGDGLTVAAKVYRSMMTYLSEGSNPEDWNIPEGLYRNGEFV

>34

AMKPITDYAPAIEYGVYDSTATMVNDIPYNYPGTSTPVYNWDRAYFGNITLQYALQQSRNVTAVETLNKVGLDRAKTFLNGLGIDYPSMHYANAISSNTTESNKQYGASSEKMAAAYAAFANGGIYHKPMYINKVVFSDGSKKEFSDVGTRAMKETTAYMMTEMMKTVLAYGTGRGAYLPWLAQAGKTGTSNYTDDEIEKHIKNTGYVAPDEMFVGYTRKYSMAVWTGYSNRLTPIVGDGFLVAAKVYRSMITYLSEDTHPEDWTMPDGLFRNGEFV

>35

AMKPITDYAPAIEYGVYDSTATMVNDIPYNYPGTSTPVYNWDRAYFGNITLQYALQQSRNVTAVETLNKVGLDRAKTFLNGLGIDYPSMHYANAISSNTTESNKQYGASSEKMAAAYAAFANGGIYHKPMYINKVVFSDGSEKEFSDVGTRAMKETTAYMMTEMMKTVLAYGTGRGAYLPWLAQAGKTGTSNYTDDEIEKHIKNTGYVAPDEMFVGYTRKYSMAVWTGYSNRLTPIVGDGFLVAAKVYRSMMTYLSEGSNPEDWNIPEGLYRNGEFV

>36

TMKPITDYAPALEYGVYDSTATIVHDEPYNYPGTNTPVYNWDRGYFGNITLQYALQQSRNVPAVETLNKVGLNRAKTFLNGLGIDYPSIHYSNAISSNTTESDKKYGASSEKMAAAYAAFANGGTYYKPMYIHKVVFSDGSEKEFSNVGTRAMKETTAYMMTDMMKTVLVYGIGRGAYLPWLPQAGKTGTSNYTDEEIEKYIKNTGYVAPDEMFVGYTRKYAMAVWTGYSNRLTPLVGDGLTVAAKVYRSMMTYLSEGSNPEDWNIPEGLYRNGEFV

>37

TMKPITDYAPALEYGVYDSTATIVHDEPYNYPGTNTPVYNWDRGYFGNITLQYALQQSRNVPAVETLNKVGLNRAKTFLNGLGIDYPSIHYSNAISSNTTESDKKYGASSEKMAAAYAAFANGGTYYKPMYIHKVVFSDGSEKEFSNVGTRAMKETTAYMMTDMMKTVLSYGTGRNAYLAWLPQAGKTGTSNYTDEEIENHIKTSQFVAPDELFAGYTRKYSMAVWTGYSNRLTPLVGNGLTVAAKVYRSMMTYLSEGRNPEDWNIPEGLYRNGEFV

>38

SMKPITDYAPALEYGVYDSTATIVHDVPYNYPGTDTPLYNWDHGYFGNITIQYALQQSRNVTAVETLNKVGLDRAKTFLNGLGIDYPSMHYANAISSNTTESNKQYGASSEKMAAAYAAFANGGIYHKPMYINKVVFSDGSEKEFSDAGTRAMKETTAYMMTEMMKTVLVYGIGRGAYLPWLPQAGKTGTSNYTDEEIEKYVKNTGYVAPDESFVGYTRKYSMAVWTGYSNRLTPVIGSGFTVASAVYRSMMTYLSEDDHPGDWTMPEGLYRSGEYV

>39

AMKPITDYAPAIEYGVYDSTATMVNDIPYNYPGTSTPVYNWDRAYFGNITLQYALQQSRNVTAVETLNKVGLDRAKTFLNGLGIDYPSMHYANAISSNTTESNKQYGASSEKMAAAYAAFANGGIYHKPMYINKVVFSDGSKKEFSDVGTRAMKETTAYMMTEMMKTVLAYGTGRGAYLPWLAQAGKTGTSNYTDDEIEKHIKNTGYVAPDEMFVGYTRKYSMAVWTGYSNRLTPIVGDGFLVAAKVYRSMMTYLSEGSNPEDWNIPEGLYRNGEFV

>40

TMKPITDYAPALEYGVYDSTATIVHDEPYNYPGTDTPVYNWDRGYFGNITIQYALQQSRNVTAVETLNKVGLDRAKTFLNGLGIDYPSMHYANAISSNTTESNKQYGASSEKMAVAYAAFANGGIYHKPMYINKIVFSDGSEKEFSDAGTRAMKETTAYMMTEMMKTVLVYGTGRGAYLPWLPQAGKTGTSNYTDEEIEKYIKNTGYVAPDEMFVGYTRKYAMAVWTGYSNRLTPLVGNGLTVAAKVYRSMMTYLSEGSNPEDWNIPEGLYRNGEFV

>41

SMKPITDYAPALEYGVYDSTASIVHDVPYNYPGTDTPLYNWDHVYFGNITIQYALQQSRNVTAVETLNKVGLDRAKTFLNGLGIDYPSMHYANAISSNTTESNKQYGASSEKMAAAYAAFANGGIYHKPMYINKVVFSDGSEKEFSDVGTRAMKETTAYMMTEMMKTVLAYGTGRGAYLPWLAQAGKTGTSNYTDDEIEKHIKNTGYVAPDEMFVGYTRKYSMAVWTGYSNRLTPIVGDGFLVAAKVYRSMITYLSEDTHPEDWTMPDGLFRNGEFV

>42

SMKPITDYAPALEYGVYDSTASIVHDVPYNYPGTDTPLYNWDHVYFGNITIQYALQQSRNVTAVETLNKVGLNRAKTFLNGLGIDYPSIHYSNAISSNTTESDKKYGASSEKMAAAYAAFANGGTYYKPMYIHKVVFSDGSEKEFSNVGTRAMKETTAYMMTEMMKTVLAYGTGRGAYLPWLAQAGKTGTSNYTDDEIEKHIKNTGYVAPDEMFVGYTRKYSMAVWTGYSNRLTPIVGDGFLVAAKVYRSMITYLSEDTHPEDWTMPDGLFRNGEFV

>43

TMKPITDYAPAIEYGVYDSTATMVNDIPYNYPGTSTPVYNWDRAYFGNITLQYALQQSRNVPAVETLNKVGLDRAKNFLNGLGIDYPSIHYSNAISSNTTESSKQYGASSEKMATAYAAFANGGIYHKPMYINKVVFSDGSEKEFSDAGTRAMKETTAYMMTEMMKTVLVYGIGRGAYLPWLPQAGKTGTSNYTDEEIEKYIKNTGYVAPDEMFVGYTRKYSMAVWTGYTNRLTPIVGDGLTVAAKVYRSMMTYLSEGSNPEDWNIPEGLYRNGEFV

>44

TMKPITDYAPALEYGVYDSTATIVHDEPYNYPGTNTPVYNWDRGYFGNITLQYALQQSRNVPAVETLNKVGLNRAKTFLNGLGIDYPSIHYSNAISSNTTESDKKYGASSEKMAAAYAAFANGGTYYKPMYIHKVVFSDRSEKEFSNVGTRAMKETTAYMMTDMMKTVLTYGTGRNAYLAWLPQAGKTGTSNYTDEEIENHIKTSQFVAPDELFAGYTRKYSMAVWTGYSNRLTPLVGNGLTVAAKVYRSMMTYLSEGSNPEDWNIPEGLYRNGEFV

>45

AMKPITDYAPAIEYGVYDSTATMVNDIPYNYPGTSTPVYNWDRAYFGNITLQYALQQSRNVTAVETLNKVGLDRAKTFLNGLGIDYPSMHYANAISSNTTESNKQYGASSEKMAAAYAAFANGGIYHKPMYINKVVFSDGSKKEFSDVGTRAMKETTAYMMTEMMKTVLAYGTGRGAYLPWLAQAGKTGTSNYTDDEIEKHIKNTGYVAPDEMFVGYTRKYSMAVWTGYSNRLTPIVGDGFLVAAKVYRSMITYLSEGSNPEDWNIPEGLYRNGEFV

>46

TMKPITDYAPALEYGIYDSTATIVHDEPYNYPGTNTPVYNWDRGYFGNITLQYALQQSRNVPAVETLNKVGLNRAKTFLNGLGIDYPSLHYSNAISSNTTESDKKYGASSEKMAAAYAAFANGGTYYKPMYIHKVVFSDGSEKEFSNVGTRAMKETTAYMMTDMMKTVLVYGIGRGAYLPWLPQAGKTGTSNYTDEEIEKYIKNTGYVAPDEMFVGYTRKYAMAVWTGYSNRLTPLVGDGLTVAAKVYRSMMTYLSEGSNPEDWNMPEGLYRNGEFV

>47

SMKPITDYAPALEYGVYDSTASIVHDVPYNYPGTDTPVYNWDHGYFGNITIQYALQQSRNVTAVETLNKVGLDRAKTFLNGLGIDYPSMYYANAISSNTTESNKQYGASSEKMAVAYAAFANGGIYHKPMYINKIVFSDGSEKEFSDAGTRAMKETTAYMMTEMMKTVLVYGIGRGAYLPWLPQAGKTGTSNYTDEEIEKYVKNTGYVAPDESFVGYTPKYSMAVWTGYSNRFTPIVGDGFLVAAKVYRSMISYLSEDEQPEDWTMPDGLFRNGEFI

>48

SMKPITDYAPALEYGVYDSTASIVHDVPYNYPGTDTPLYNWDHVYFGNITIQYALQQSRNVTAVETLNKVGLDRAKTFLNGLGIDYPSMHYANAISSNTTESNKKYGASSEKMAAAYAAFANGGIYHKPMYINKIVFSDGSEKEFSDAGTRAMKETTAYMMTEMMKTVLTYGTGRGAYLPWLPQAGKTGTSNYTDEEIEKYIKNTGYVAPDEMFVGYTRKYSMAVWTGYSNRLTPIIGDGFLVAGRVYRSMMTYLSEGSNPEDWNIPEGLYRNGEFV

>49

TMKPITDYAPALEYGVYDSTATIVHDEPYNYPGTNTPVYNWDRGYFGNITLQYALQQSRNVPAVETLNKVGLNRAKTFLNGLGIDYPSIHYSNAISSNTTESDKKYGASSEKMAAAYAAFANGGTYYKPMYIHKVVFSDGSEKEFSNVGTRAMKETTAYMMTDMMKTVLAYGTGRNAYLAWLPQAGKTGTSNYTDEEIENHIKTSQFVAPDELFAGYTRKYSMAVWTGYSNRLTPLVGNGLTVAAKVYRSMMTYLSKGSNPEDWNIPEGLYRNGEFV

>50

TMKPITDYAPALEYGVYDSTATIVHDEPYNYPGTNTPVYNWDRGYFGNITLQYALQQSRNVPAVETLNKVGLNRAKTFLNGLGIDYPSIHYSNAISSNTTESDKKYGASSEKMAAAYAAFANGGTYYKPMYIHKVVFSDGSEKQFSNVGTRAMKETTAYMMTDMMKTVLSYGTGRNAYLAWLPQAGKTGTSNYTDEEIENHIKTSQFVAPDELFAGYTRKYSMAVWTGYSNRLTPLVGNGLTVAAKVYRSMMTYLSEGSNPEDWNIPEGLYRNGEFV

>51

TMKPITDYAPALEYGVYDSTATIVHDEPYNYPGTDTPVYNWDRGYFGNITLQYALQQSRNVPAVETLNKVGLNRAKTFLNGLGIDYPSLHYSNAISSNTTESDKKYGASSEKMAAAYAAFANGGTYYKPMYIHKVVFSDGGEKEFSNVGTRAMKETTAYMMTDMMKTVLVYGIGRGAYLPWLPQAGKTGTSNYTDEEIEKYIKNTGYVAPDEMFVGYTRKYAMAVWTGYSNRLTPLVGDGLTVAAKVYRSMMTYLSEGSNPEDWNIPEGLYRNGEFV

>52

AMKPITDYAPAIEYGVYDSTATMVNDIPYNYPGTSTPVYNWDRAYFGNITLQYALQQSRNVTAVETLNKVGLDRAKTFLNGLGIDYPSMYYANAISSNTTESNKQYGASSEKMAVAYAAFANGGIYHKPMYINKIVFSDGSEKEFSDAGTRAMKETTAYMMTEMMKTVLVYGIGRGAYLPWLPQAGKTGTSNYTDEEIEKYVKNTGYVAPDESFVGYTRKYAMAVWTGYSNRLTPLVGNGLTVAAKVYRSMMTYLSEGSNPEDWNIPEGLYRNGEFV

>53

SMKPITDYAPAIEYGVYDSTATMVNDIPYNYPGTSTPVYNWDRAYFGNITLQYALQQSRNVTAVETLNKVGLDRAKTFLNGLGIDYPSMYYANAISSNTTESNKQYGASSEKMAVAYAAFANGGIYHKPMYINKIVFSDGSEKEFSDAGTRAMKETTAYMMTEMMKTVLVYGTGRGAYLPWLPQAGKTGTSNYTDEEIEKYIKNTGYVAPDEMFVGYTRKYAMAVWTGYSNRLTPIVGDDFLVAAKVYRSMISYLSEDDHPGDWTMPEGLYRSGEFV

>54

SMKPITDYAPAIEYGVYDSTATMVNDIPYNYPGTSTPVYNWDRAYFGNITLQYALQQSRNVTAVETLNKVGLDRAKTFLNGLGIDYPSMYYANAISSNTTESNKQYGASSEKMAVAYAAFANGGIYHKPMYINKIVFSDGSEKEFSDAGTRAMKETTAYMMTEMMKTVLVYGTGRGAYLPWLPQAGKTGTSNYTDEEIEKYIKNTGYVAPDEMFVGYTRKYSMAVWTGYSNRLTPIVGDVFLVAAKVYRSMITYLSEDDQPGDWTMPDGLYRNGEFV

>55

TMKPITDYAPALEYGVYDSTATIVHDEPYNYPGTDIPVYNWDRGYFGNITLQYALQQSRNVPAVETLNKVGLNRAKTFLNGLGIDYPSIHYSNAISSNTTESNKKYGASSEKMAAAYAAFANGGTYYKPMYIHKVVFSDGSEKEFSNVGTRAMKETTAYMMTDMMKTVLTYGTGRNAYLAWLPQAGKTGTSNYTDEEIENHIKTSQFVAPDELFAGYTRKYSMAVWTGYSNRLTPLVGNGLTVAAKVYRSMMTYLSEGSNPEDWNIPEGIYRNGQFV

>56

TMKPITDYAPALEYGVYDSTATIVHDEPYNYPGTNTPVYNWDRGYFGNITLQYALQQSRNVPAVETLNKVGLNRAKTFLNGLGIDYPSIHYSNAISSNTTESNKQYGASSEKMAAAYAAFANGGIYHKPMYINKVVFSDGSEKEFSDVGTRAMKETTAYMMTEMMKTVLAYGTGRGAYLPWLAQAGKTGTSNYTDDEIEKHIKNTGYVAPDEMFVGYTRKYSMAVWTGYSNRLTPIVGDGFLVAAKVYRSMMTYLSEGSNPEDWNIPEGLYRNGEFV

>57

AMKPITDYAPAIEYGVYDSTATMVNDIPYNYPGTSTPVYNWDRAYFGNITLQYALQQSRNVTAVETLNKVGLDRAKTFLNGLGIDYPSMYYANAISSNTTESNKQYGASSEKMAVAYAAFANGGIYHKPMYINKIVFSDGSEKEFSDAGTRAMKETTAYMMTEMMKTVLTYGTGRGAYLPWLPQAGKTGTSNYTDEEIEKYIKNTGYVAPDEMFVGYTRKYSMAVWTGYSNRLTPIVGDGFLVAAKVYRSMMTYLSEGSNPEDWNIPEGLYRNGEFV

>58

AMKPITDYAPAIEYGVYDSTATMVNDIPYNYPGTSTPVYNWDRAYFGNITLQYALQQSRNVTAVETLNKVGLDRAKTFLNGLGIDYPSIHYSNAISSNTTESSKQYGASSEKMAAAYAAFANGGIYHKPMYINKIVFSDGSEKEFSDAGTRAMKETTAYMMTEMMKTVLTYGTGRGAYLPWLSQAGKTGTSNYTDDEIEKYIKNTGYVAPDELFAGYTRKYSMAVWTGYSNRLTPLVGNGLTVAAKVYRSMMTYLSEGSNPEDWNIPEGLYRNGEFV

>59

TMKPITDYAPALEYGVYDSTATIVHDEPYNYPGTDTPVYNWDRGYFGNITLQYALQQSRNVPAVETLNKVGLNRAKTFLNGLGIDYPSLHYSNAISSNTTESDKKYGASSEKMAAAYAAFANGGTYYKPMYIHKVVFSDGSEKEFSNVGTRAMKETTAYMMTDMMKTVLVYGIGRGAYLPWLPQAGKTGTSNYTDEEIEKYIKNTGYVAPDEMFVGYTRKYSMAVWTGYSNRLTPLVGNGLTVAAKVYRSMMTYLSEGSNPEDWNIPEGLYRNGEFV

>60

SMKPITDYAPALEYGVYDSTASIVHDVPYNYPGTDTPLYNWDHVYFGNITIQYALQQSRNVTAVETLNKVGLDRAKTFLNGLGIDYPSMHYANAISSNTTESNKKYGASSEKMAAAYAAFANGGIYHKPMYINKIVFSDGSEKEFSDAGTRAMKETTAYMMTEMMKTVLTYGTGRGAYLPWLPQAGKTGTSNYTDEEIEKYIKNTGYVAPDEMFVGYTRKYSMAVWTGYSNRLTPIIGDGFLVAGRVYRSMMTYLSEDNHPGDWTMPEGLYRSGEFV

>61

TMKPITDYAPALEYGVYDSTATIVHDEPYNYPGTNIPVYNWDRGYFGNITLQYALQQSRNVPAVETLNKVGLNRAKTFLNGLGIDYPSIHYSNAISSNTTESDKKYGASSEKMAAAYAAFANGGTYYKPMYIHKVVFSDGSEKEFSNVGTRAMKETTAYMMTDMMKTVLTYGTGRNAYLAWLPQAGKTGTSNYTDEEIENHIKTSQFVAPDELFAGYTRKYSMAVWTGYSNRLTPLVGNGLTVAAKVYRSMMTYLSEGSNPEDWNIPEGLYRNGEFV

>62

TMKPITDYAPALEYGVYDSTATIVHDEPYNYPGTNTPVYNWDRGYFGNITLQYALQQSRNVPAVETLNKVGLNRAKTFLNGLGIDYPSIHYSNAISSNTTESDKKYGASSEKMAAAYAAFANGGTYYKPMYIHKVVFSDGSEKEFSNVGTRAMKETTAYMMTDMMKTVLTYGTGRNAYLAWLPQAGKTGTSNYTDEEIENHIKTSQFVAPDELFAGYTRKYSMAVWTGYSNRLTPLVGNGLTVAAKVYRSMMTYLSEGSNPEDWNMPEGLYRNGEFV

>63

TMKPITDYAPALEYGVYDSTAAIVHDEPYNYPGTNTPVYNWDRGYFGNITLQYALQQSRNVPAVETLNKVGLNRAKTFLNGLGIDYPSIHYSNAISSNTTESDKKYGASSEKMAAAYAAFANGGTYYKPMYIHKVVFSDGSEKEFSNVGTRAMKETTAYMMTDMMKTVLTYGTGRNAYLAWLPQAGKTGTSNYTDEEIENHIKTSQFVAPDELFAGYTRKYSMAVWTGYSNRLTPLVGNGLTVAAKVYRSMMTYLSEGSNPEDWNIPEELYRNGEFV

>64

TMKPITDYAPALEYGVYDSTATIVHDEPYNYPGTNTPVYNWDRGYFGNITLQYALQQSRNVPAVETLNKVGLNRAKTFLNGLGIDYPSIHYSNAISSNTTESDKKYGASSEKMAAAYAAFANGGTYYKPMYIHKVVFSDGSEKEFSNVGTRAMKETTAYMMTDMMKTVLSYGTGRNAYLAWLPQAGKTGTSNYTDEEIENHIKTSQFVAPDELFAGYTRKYSMAVWTGYSNRLTPLVGNDLTVAAKVYRSMMTYLSEGSNPEDWNIPEGLYRNGEFV
