# Supplementary material for: Strain features and distributions in pneumococci from children with invasive disease before and after 13-valent conjugate vaccine implementation in the USA
Source: Clin Microbiol Infect. 2016 Jan;22(1):60.e9–60.e29. doi: 10.1016/j.cmi.2015.08.027 (PMC4721534; doi:10.1016/j.cmi.2015.08.027)
Supplement: Table S4 — PBP2b transpeptidase domain sequences compiled from approximately 1500 ABCs isolates collected during 1998–2013. [file mmc4.docx]

sTable 4. PBP2b Transpeptidase domain sequences compiled from approximately 1500 ABCs isolates collected during 1998-2013.

>0

TNVFVPGSVVKAATISSGWENGVLSGNQTLTDQSIVFQGSAPINSWYTQAYGSFPITAVQALEYSSNTYMVQTALGLMGQTYQPNMFVGTSNLESAMEKLRSTFGEYGLGTATGIDLPDESTGFVPKEYSFANYITNAFGQFDNYTPMQLAQYVATIANNGVRVAPRIVEGIYGNNDKGGLGDLIQQLQPTEMNKVNISDSDMSILHQGFYQVAHGTSGLTTGRAFSNGALVSISGKTGTAESYVADGQQATNTNAVAYAPSDNPQIAVAVVFPHNTN

>1

TNVFVPGSVVKAATISSGWENGVLSGNQTLTDQPIVFQGSAPINSWYTQAYGSFPITAVEALEYSSNAYMVQTALGIMGQTYQPNMFVLTNNLESAMGKLRSTFAEYGLGASTGIDLPDESTGFIPKEYNFANYITNAFGQFDNYTPMQLAQYVGTIANNGVRIAPHIVEGIYGNNEQGGLGNLIQSVESKEMNKINISESDVSILQQGFYQVSHGGSALTTGRAFSNGASVSISGKTGTAESYVEGGQEANNTNAVAYAPSDNPQIAVAVVFPHNTN

>2

TNVFVPGSVVKAATISSGWENGVLSGNQTLTDQSIVFQGSAPINSWYTQAYGSFPITAVQALEYSSNTYMVQTALGLMGQTYQPNMFVGTSNLESAMEKLRSTFGEYGLGTATGIDLPDESTGFVPKEYSFANYITNAFGQFDNYTPMQLAQYVATIANDGVRVAPRIVEGIYGNNDKGGLGDLIQQLQPTEMNKVNISDSDMSILHQGFYQVAHGTSELTTGRAFSNGALVSISGKTGTAESYVADGQQATNTNAVAYAPSDNPQIAVAVVFPHNTN

>3

TNVFVPGSVVKAATISSGWENGVLSGNQTLTDQSIVFQGSAPINSWYTQAYGSFPITAVQALEYSSNTYMVQTALGLMGQTYQPNMFVGTSNLESAMEKLRSTFGEYGLGTATGIDLPDESTGFVPKEYSFANYITNAFGQFDNYTPMQLAQYVATIANNGVRVAPRIVEGIYGNNDKGGLGDLIQQLQPTEMNKVNISDSDMSILHQGFYQVAHGTSGLTTGRAFSNGALVSISGKTGTAESYVADGQQATNTNAVAYAPYDNPQIAVAVVFPHNTN

>4

TNVFVPGSVVKAATISSGWENGVLSGNQTLTDQSIVFQGSAPINSWYTQAYGSFPITAVQALEYSSNTYMVQTALGLMGQTYQPNMFVGTSNLESAMEKLRSTFGEYGLGTATGIDLPDESTGFVPKEYSFANYITNAFGQFDNYTPMQLAQYVATIANNGVRVAPRIVEGIYGNNDKGGLGDLIQQLQPTEMNKVNISDSDMSILHQGFYQVAHGTSELTTGRAFSNGALVSISGKTGTAESYVADGQQATNTNAVAYAPSDNPQIAVAVVFPHNTN

>5

TNVFAPGSVVKAATISSGWENGVLSGNQTLTDQSIVFQGSAPINSWYTQAYGSFPITAVQALEYSSNTYMVQTALGLMGQTYQPNMFVGTSNLEFAMEKLRSTFGEYGLGTATGIDLPDESTGFVPKEYSFANYITNAFGQFDNYTPMQLAQYVATIANDGVRVAPRIVEGIYGNNDKGGLGDLIQQLQPTEMNKVNISDSDMSILHQGFYQVAHGTSELTTGRAFSNGALVSISGKTGTAESYVADGQQATNTNAVAYAPSDNPQIAVAVVFPHNTN

>6

TNVFVPGSVVKAATISSGWENGVLSGNQTLTDQSIVFQGSAPINSWYTQAYGSFPITAVQALEYSSNTYMVQTALGLMGQTYQPNMFVGTSNLESAMEKLRSTFGEYGLGTATGIDLPDESTGFVPKEYSFANYITNAFGQFDNYTPMQLAQYVATIANNGVRVAPRIVEGIYGNNDKGGLGDLIQQLQPTEINKVNISDSDMSILHQGFYQVAHGTSGLTTGRAFSNGALVSISGKTGTAESYVADGQQATNTNAVAYAPSDNPQIAVAVVFPHNTN

>7

TNVFVPGSVVKAATISSGWENGVLSGNQTLTDQPIVFQGSAPIYSWYKLAYGSFPITAVEALEYSSNAYMVQTALGIMGQTYQPNMFVGTSNLETAMGKLRATFGEYGLGAATGIDLPDESTGFVPKEYSFANYITNAFGQFDNYTPMQLAQYVATIANDGVRVAPRIVEGIYGNNDKGGLGDLIQQLQPTEMNKVNISDSDMSILHQGFYQVAHGTSGLTTGRAFSNGALVSISGKTGTAESYVAGGQEANNTNAVAYAPSDNPQIAVAVVFPHNTN

>8

TNVFVPGSVVKAATISSGWENGVLSGNQTLTDQSIVFQGSAPIYSWYKLAYGSFPITAVEALEYSSNAYMVQTALGIMGQTYQPNMFVGTSNLETAMGKLRATFGEYGLGAATGIDLPDESTGFVPKEYSFANYITNAFGQFDNYTPMQLAQYVATIANDGVRVAPRIVEGIYGNNDKGGLGELIQAIDTKEINKVNISESDMAILHQGFYQVSHGTSPLTTGRAFSDGATVSISGKTGTAESYVEGGQEANNTNAVAYAPSDNPQIAVAVVFPHNTN

>9

TNVFVPGSVVKAATISSGWENGVLSGNQTLTDQPIVFQGSAPINSWYTQAYYGSFPITAVEALEYSSNAYMVQTALGIMGQTYQPNMFVLTNNLESAMGKLRSTFAEYGLGASTGIDLPDESTGFIPKEYNFANYITNAFGQFDNYTPMQLAQYVATIANNGVRVAPRIVEGIYGNNDKGGLGNLIQQLQPTEMNKVNISDSDMSILHQGFYQVAHGTSGLTTGRAFSNGALVSISGKTGTAESYVADGQQATNTNAVAYAPSDNPQIAVAVVFPHNTN

>10

TNVFVPGSVVKAATISSGWENGVLSGNQTLTDQSIVFQGSAPINSWYTQAYGSFPITAVQALEYSSNTYMVQTALGLMGQTYQPNMFVGTSNLESAMEKLRSTFGEYGLGTETGIDLPDESTGFVPKEYSFANYITNAFGQFDNYTPMQLAQYVATIANNGVRVAPRIVEGIYGNNDKGGLGDLIQQLQPTEMNKVNISDSDMSILHQGFYQVAHGTSGLTTGRAFSNGALVSISGKTGTAESYVADGQQATNTNAVAYAPSDNPQIAVAVVFPHNTN

>11

TNVFVPGSVVKAATISSGWENGVLSGNQTLTDQPIVFQGSAPIYSWYKLAYGSFPITAVEALEYSSNAYMVQTALGIMGQTYQPNMFVGTSNLETAMGKLRATFGEYGLGAATGIDLPDESTGFVPKEYSFANYITNAFGQFDNYTPMQLAQYVATIANDGVRVAPRIVEGIYGNNDKGGLGELIQAIDTKEINKVNISESDMAILHQGFYQVSHGTSPLTTGRAFSDGATVSISGKTGTGESYVAGGQEANNTNAVAYAPTENPQIAVAVVFPHNTN

>12

TNVFVPGSVVKAATISSGWENGVLSGNQTLTDQPIVFQGSAPIYSWYKLAYGSFPITAVEALEYSSNAYMVQTALGIMGQTYQPNMFVGTSNLETAMGKLRATFGEYGLGAATGIDLPDESTGFVPKEYSFANYITNSFGQFDNYTPMQLAQYVATIANNGVRVAPRIVEGIYGNNDKGGLGDLIQQLQPTEMNKVNISDSDMSILHQGFYQVAHGTSGLTTGRAFSNGALVSISGKTGTAESYVADGQQATNTNAVAYAPSDNPQIAVAVVFPHNTN

>13

TNVFVPGSVVKAATISSGWENGVLSGNQTLTDQPIVFQGSAPINSWYTQAYGSFPITAVEALEYSSNAYMVQTALGIMGQTYQPNMFVLTNNLESAMGKLRSTFAEYGLGASTGIDLPDESTGFIPKEYNFANYITNAFGQFDNYTPMQLAQYVATIANDGVRVAPRIVEGIYGNNDKGGLGDLIQQLQPTEMNKVNISDSDMSILHQGFYQVAHGTSGLTTGRAFSNGALVSISGKTGTAESYVADGQQATNTNAVAYAPSDNPQIAVAVVFPHNTN

>14

TNVFVPGSVVKAATISSGWENGVLSGNQTLTDQPIVFQGSAPIYSWYKLAYGSFPITAVEALEYSSNAYVVQTALGIMGQTYQPNMFVGTSNLESAMGKLRSTFGEYGLGSATGIDLPDESTGLVPKEYNFANFITNAFGQFDNYTPMQLAQYVATIANNGVRLAPHIVEGIYDNNDKGGLGELIQAIDTKEINKVNISESDMAILHQGFYQVSHGTSPLTTGRAFSDGATVSISGKTGTGESYVAGGQEANNTNAVAYAPTENPQIAVAVVFPHNTN

>15

TNVFVPGSVVKAATISSGWENGVLSGNQTLTDQSIVFQGSAPINSWYTAFSRPMPITAVQALEYSSNAYMVQTALGLMGQTYQPNMFVGTSNLESAMGKLRSTFGEYGLGSATGIDLPDESTGFIPKEYSFANYITNAFGQFDNYTPMQLAQYVATIANDGVRVAPRIVEGIYGNNDKGGLGDLIQQLQPTEMNKVNISDSDMSVLHQGFYQVAHGTSGLTTGRAFSNGALVSISGKTGTAESYVADGQQATNTNAVAYAPSDNPQIAVAVVFPHNTN

>16

TNVFVPGSVVKAATISSGWENGVLSGNQTLTDQPIVFQGSAPIYSWYKLAYGSFPITAVEALEYSSNAYMVQTALGIMGQTYQPNMFVGTSNLETAMGKLRATFGEYGLGAATGIDLPDESTGFVPKEYSFANYITNAFGQFDNYTPMQLAQYVATIANDGVRVAPRIVEGIYGNNDKGGLGDLIQQLQPTEMNKVNISDSDMSILHQGFYQVAHGTSGLTTGRAFSNGALVSISGKTGTAESYVADGQQATNTNAVAYAPSDNPQIAVAVVFPHNTN

>17

TNVFVPGSVVKAATISSGWENGVLSGNQTLTDQPIVFQGSAPIYSWYKLAYGSFPITAVEALEYSSNAYMVQTALGIMGQTYQPNMFVGTSNLETAMGKLRATFGEYGLGAATGIDLPDESTGFVPKDYSFANYITNAFGQFDNYTPMQLAQYVATIANDGVRVAPRIVEGIYGNNDKGGLGDLIQQLQPTEMNKVNISDSDMSILHQGFYQVAHGTSGLTTGRAFSNGALVSISGKTGTAESYVAGGQEANNTNAVAYAPSDNPQIAVAVVFPHNTN

>18

TNVFVPGSVVKAATISSGWENGVLSGNQTLTDQPIVFQGSAPINSWYTQAYDSFPITAVEALEYSSNAYMVQTALGIMGQTYQPNMFVLTNNLESAMGKLRSTFAEYGLGASTGIDLPDESTGFIPKEYNFANYITNAFGQFDNYTPMQLAQYVGTIANNGVRIAPHIVEGIYGNNEQGGLGNLIQSVETKEMNKINISESDVSILQQGFYQVSHGGSALTTGRAFSNGAAVSISGKTGTAESYVNGGQEANNTNAVAYAPSDNPQIAVAVVFPHNTN

>19

TNVFVPGSVVKAATISSGWENGVLSGNQTLTDQPIVFQGSAPIYSWYKLAYGSFPITAVEALEYSSNAYMVQTALGIMGQTYQPNMFVGTSNLETAMGKLRATFDEYGLGAATGIDLPDESTGFVPKEYSFANYITNAFGQFDNYTPMQLAQYVATIANDGVRVAPRIVEGIYGNNDKGGLGDLIQQLQPTEMNKVNISDSDMSILHQGFYQVAHGTSGLTTGRAFSNGALVSISGKTGTAESYVAGGQEANNTNAVAYAPSDNPQIAVAVVFPHNTN

>20

TNVFVPGSVVKAATISSGWENGVLSGNQTLTDQSIVFQGSAPINSWYTQAYGSFPITAVQALEYSSNTYMVQTALGLMGQTYQPNMFVGTSNLESAMEKLRSTFGEYGLGTATGIDLPDESTGFVPKEYSFANYITNAFGQFDNYTPMQLAQYVATIANNGVRVAPRIVEGIYGNNDKGGLGDLIQQLQPTEMNKVNISDSDMSILHQGFYQIAHGTSGLTTGRAFSNGALVSISGKTGTAESYVADGQQATNTNAVAYAPSDNPQIAVAVVFPHNTN

>21

TNVFVPGSVVKAATISSGWENGVLSGNQTLTDQPIVFQGSAPINSWYTQAYGSFPITAVEALEYSSNAYMVQTALGIMGQTYQPNMFVLTNNLESAMGKLRSTFAEYGLGASTGIDLPDESTGFIPKEYNFANYITNAFGQFDNYTPMQLAQYVGTIANNGVRIAPHIVEGIYGNNEQGGLGNLIQSVESKEMNKINISESDVSILQQGFYQVSHGGSALTTGRAFSNGALVSISGKTGTAESYVADGQQATNTNAVAYAPSDNPQIAVAVVFPHNTN

>22

TNVFVPGSVVKAATISSGWENGVLSGNQTLTDQSIVFQGSAPINSWYTQAYGSFPITAVQALEYSSNAYMVQTALGLMGQTYQPNMFVGTSNLESAMGKLRSTFGEYGLGSATGIDLPDESTGFVLKDYSFANYITNAFGQFDNYTPMQLAQYVATIANDGVRVAPRIVEGIYGNNDKGGLGDLIQQLQPTEMNKVNISDSDMSILHQGFYQVAHGTSGLTTGRAFSNGAAVSISGKTGTAESYVEGGQEANNTNAVAYAPSDNPQIAVAVVFPHNTN

>23

TNVFVPGSVVKAATISSGWENGVLSGNQTLTDQSIVFQGSAPIYSWYKLAYGSFPITAVEALEYSSNAYMVQTALGIMGQTYQPNMFVGTSNLESAMEKLRSTFGEYGLGTATGIDLPDESTGFVPKEYSFANYITNAFGQFDNYTPMQLAQYVATIANNGVRVAPRIVEGIYGNNDKGGLGDLIQQLQPTEMNKVNISDSDMSILHQGFYQVAHGTSGLTTGRAFSNGALVSISGKTGTAESYVADGQQATNTNAVAYAPSDNPQIAVAVVFPHNTN

>24

TNVFVPGSVVKAATISSGWENGVLSGNQTLTDQPIVFQGSAPIYSWYKLAYGSFPITAVEALEYSSNAYMVQTALGIMGQTYQPNMFVGTSNLETAMGKLRATFGEYGLGTATGIDLPDESTGFVPKEYSFANYITNAFGQFDNYTPMQLAQYVATIANNGVRVAPRIVEGIYGNNDKGGLGDLIQQLQPTEMNKVNISDSDMSILHQGFYQVAHGTSGLTTGRAFSNGALVSISGKTGTAESYVADGQQATNTNAVAYAPSDNPQIAVAVVFPHNTN

>25

TNVFVPGSVVKAATISSGWENGVLSGNQTLTDQPIVFQGSAPIYSWYKLAYGSFPITAVEALEYSSNAYMVQTALGIMGQTYQPNMFVGTSNLESAMGKLRSTFGEYGLGSATGIDLPDESTGFVPKDYSFANYITNTFGQFDNYTPMQLAQYVATIANDGVRVAPRIVEGIYDNNDKGGLGELIQAIDTKEINKVNISESDMAILHQGFYQVSHGTSGLTTGRAFSNGALVSISGKTGTAESYVADGQQATNTNAVAYAPSDNPQIAVAVVFPHNTN

>26

TNVFVPGSVVKAATISSGWENGVLSGNQTLTDRSIVFQGSAPINSWYTQAYGSFPITAVQALEYSSNTYMVQTALGLMGQTYQPNMFVGTSNLESAMEKLRSTFGEYGLGTATGIDLPDESTGFVPKEYSFANYITNAFGQFDNYTPMQLAQYVATIANNGVRVAPRIVEGIYGNNDKGGLGDLIQQLQPTEMNKVNISDSDMSILHQGFYQVAHGTSGLTTGRAFSNGALVSISGKTGTAESYVADGQQATNTNAVAYAPSDNPQIAVAVVFPHNTN

>27

TNVFVPGSVVKAATISSGWENGVLSGNQTLTDQSIVFQGSAPINSWYTQAYGSFPITAVQALEYSSNAYMVQTALGLMGQTYQPNMFVGTSNLESAMGKLRSTFGEYGLGSATGIDLPDESTGFVPKDYSFANYITNAFGQFDNYTPMQLAQYVATIANDGVRVAPRIVEGIYGNNDKGGLGDLIQQLQPTEMNKVNISDSDMSILHQGFYQVAHGTSGLTTGRAFSNGAAVSISGKTGTAESYVEGGQEANNTNAVAYAPSDNPQIAVAVVFPHNTN

>28

TNVFVPGSVVKAATISSGWENGVLSGNQTLTDQPIVFQGSAPIYSWYKLAYGSFPITAVEALEYSSNAYVVQTALGIMGQTYQPNMFVGTNNLESAMGKLRSTFGEYGLGSATGIDLPDESTGLVPKEYNFANFITNAFGQFDNYTPMQLAQYVATIANNGVRLAPHIVEGIYDNNDKGGLGELIQAIDTKEINKVNISESDMAILHQGFYQVSHGTSPLTTGRAFSDGATVSISGKTGTGESYVAGGQEANNTNAVAYAPTENPQIAVAVVFPHNTN

>29

TNVFVPGSVVKAATISSGWENGVLSGNQTLTDQSIVFQGSAPINSWYTQAYGSFPITAVQALEYSSNAYMVQTALGLMGQTYQPNMFVGTSKLESAMGKLRSTFGEYGLGSATGIDLPDESTGFVPKDYSFANYITNAFGQFDNYTPMQLAQYVATIANDGVRVAPRIVEGIYGNNDKGGLGDLIQQLQPTEMNKVNISDSDMSILHQGFYQVAHGTSGLTTGRAFSNGALVSISGKTGTAESYVADGQQATNTNAVAYAPSDNPQIAVAVVFPHNTN

>30

TNVFVPGSVVKAATISSGWENGVLSGNQTLTDQPIVFQGSAPIYSWYKLAYGSFPITAVEALEYSSNAYVVQTALGIMGQTYQPNMFVGTSNLESAMGKLRSTFGEYGLGSATGIDLPDESTGLVPKEYNFANFITNAFGQFDNYTPMQLAQYVATIANNGVRVAPRIVEGIYGNNDKGGLGDLIQQLQPTEMNKVNISDSDMSILHQGFYQVAHGTSGLTTGRAFSNGALVSISGKTGTAESYVADGQQATNTNAVAYAPSDNPQIAVAVVFPHNTN

>31

TNVFVPGSVVKAATISSGWENGVLSGNQTLTDQPIVFQGSAPIYSWYKLAYGSFPITAVEALEYSSNAYMVQTALGIMGQTYQPNMFVGTSNLETAMGKLRATFGEYGLGAATGIDLPDESTGFVPKEYSFANYITNAFGQFDNYTPMQLAQYVATIANNGVRVAPRIVEGIYGNNDKGGLGDLIQQLQPTEMNKVNISDSDMSILHQGFYQVAHGTSGLTTGRAFSNGALVSISGKTGTAESYVADGQQATNTNAVAYAPSDNPQIAVAVVFPHNTN

>32

TNVFVPGSVVKAATISSGWENGVLSGNQTLTDQPIVFQGSAPIYSWYKLAYGSFPITAVEALEYSSNAYMVQTALGIMGQTYQPNMFVGTSNLETAMGKLRATFGEYGLGAATGIDLPDESTGFVPKEYSFANYITNSFGQFDNYTPMQLAQYVATIANDGVRVAPRIVEGIYGNNDKGGLGDLIQQLQPTEMNKVNISDSDMSILHQGFYQVAHGTSGLTTGRAFSNGAAVSISGKTGTAESYVAGGQEANNTNAVAYAPSDNPQIAVAVVFPHNTN

>33

TNVFVPGSVVKAATISSGWENGVLSGNQTLTDQPIVFQGSAPIYSWYKLAYGSFPITAVEALEYSSNAYMVQTALGIMGQTYQPNMFVGTSNLETAMGKLRATFGEYGLGAATGIDLPDESTGFIPKEYSFANYITNAFGQFDNYTPMQLAQYVATIANDGVRVAPRIVEGIYGNNDKGGLGDLIQQLQPTEMNKVNISDSDMSILHQGFYQVAHGTSGLTTGRAFSNGALVSISGKTGTAESYVAGGQEANNTNAVAYAPSDNPQIAVAVVFPHNTN

>34

TNVFVPGSVVKAATISSGWENGVLSGNQTLTDQSIVFQGSAPINSWYTQAYGSFPITAVQALEYSSNAYMVQTALGLMGQTYQPNMFVGTSNLESAMGKLRSTFGEYGLGAATGIDLPDESTGFVPKDYNFANFITNAFGQFDNYTPMQLAQYVATIANDGVRVAPRIVEGIYGNNDKGGLGDLIQQLQPTEMNKVNISDSDMSVLHQGFYQVAHGTSGLTTGRAFSNGALVSISGKTGTAESYVADGQEATNTNAVAYAPSDNPQIAVAVVFPHNTN

>35

TNVFVPGSVVKAATISSGWENGVLSGNQTLTDQPIVFQGSAPINSWYTQAYGSFPITAVQALEYSSNAYMVQTALGLMGQTYQPNMFVGTSNLESAMGKLRSTFGEYGLGSATGIDLPDESTGFIPKDYSFANYITNAFGQFDNYTPMQLAQYVATIANDGVRVAPRIVEGIYGNNDKGGLGDLIQQLQPTEMNKVNISDSDMSVLHQGFYQVAHGTSGLTTGRAFSNGASVSISGKTGTAESYVEGGQEANNTNAVAYAPSDNPQIAVAVVFPHNTN

>36

TNVFVPGSVVKAATISSGWENGVLSGNQTLTDQPIVFQGSAPIYSWYKLAYGSFPITAVEALEYSSNAYMVQTALGIMGQTYQPNMFVGTSNLETAMGKLRATFGEYGLGAATGIDLPDESTGFVPKDYSFANYITNAFGQFDNYTPMQLAQYVATIANDGVRVAPRIVEGIYGNNDKGGLGDLIQQLQPTEMNKVNISDSDMSILHQGFYQVSHGTSPLTTGRAFSDGATVSISGKTGTGESYVAGGQEANNTNAVAYAPTENPQIAVAVVFPHNTN

>37

TNVFVPGSVVKAATISSGWENGVLSGNQTLTDQPIVFQGSAPIYSWYKLAYGSFPITAVEALEYSSNAYMVQTALGIMGQTYQPNMFVGTSNLETAMGKLRATFGEYGLGAATGIDLPDESTGFVPKEYSFANYITNAFGQFDNYTPMQLAQYVATIANNGVRLAPHIVEGIYDNNDKGGLGELIQAIDTKEINKVNISESDMAILHQGFYQVSHGTSPLTTGRAFSDGATVSISGKTGTGESYVAGGQEANNTNAVAYAPTENPQIAVAVVFPHNTN

>38

TNVFVPGSVVKAATISSGWENGVLSGNQTLTDQPIVFQGSAPIYSWYKLAYGSFPITAVEALEYSSNAYMVQTALGIMGQTYQPNMFVGTSNLETAMGKLRATFGEYGLGAATGIDLPDESTGFVPKDYSFANYITNAFGQFDNYTPMQLAQYVATIANNGVRLAPHIVEGIYDNNDKGGLGELIQAIDTKEINKVNISESDMAILHQGFYQVSHGTSPLTTGRAFSDGATVSISGKTGTGESYVAGGQEANNTNAVAYAPTENPQIAVAVVFPHNTN

>39

TNVFVPGSVVKAATISSGWENGVLSGNQTLTDQPIVFQGSAPIYSWYKLAYGSFPITAVEALEYSSNAYMVQTALGIMGQTYQPNMFVGTSNLETAMGKLRATFGEYGLGAATGIDLPDESTGFVPKEYSFANYITNAFGQFDNYTPMQLAQYVATIANDGVRVAPRIVEGIYGNNDKGGLGDLIQQLQPTEMNKVNISDSDMSVLHQGFYQVAHGTSGLTTGRAFSNGALVSISGKTGTAESYVADGQQATNTNAVAYAPSDNPQIAVAVVFPHNTN

>40

TNVFVPGSVVKAVTISSGWENGVLSGNQTLTDQSIVFQGSAPINSWYTQAYGSFPITAVQALEYSSNTYMVQTALGLMGQTYQPNMFVGTSNLESAMEKLRSTFGEYGLGTATGIDLPDESTGFVPKEYSFANYITNAFGQFDNYTPMQLAQYVATIANNGVRVAPRIVEGIYGNNDKGGLGDLIQQLQPTEMNKVNISDSDMSILHQGFYQVAHGTSGLTTGRAFSNGALVSISGKTGTAESYVADGQQATNTNAVAYAPSDNPQIAVAVVFPHNTN

>41

TNVFVPGSVVKAATISSGWENGVLSGNQTLTDQPIVFQGSAPIYSWYKLAYGSFPITAVEALEYSSNAYMVQTALGIMGQTYQPNMFVGTSNLETAMGKLRATFGEYGLGAATGIDLPDESTGFVPKEYSFANYITNAFGQFDNYTPMQLAQYVATIANDGVRVAPRIVEGIYGNNDKGGLGELIQAIDTKEINKVNISESDMAILHQGFYQVSHGTSGLTTGRAFSNGAAVSISGKTGTAESYVAGGQEANNTNAVAYAPSDNPQIAVAVVFPHNTN

>42

TNVFVPGSVVKAATISSGWENGVLSGNQTLTDQPIVFQGSAPIYSWYKLAYGSFPITAVEALEYSSNAYMVQTALGIMGQTYQPNMFVGTSNLETAMGKLRATFGEYGLGAATGIDLPDESTGFVPKDYSFANYITNAFGQFDNYTPMQLAQYVATIANDGVRVAPRIVEGIYGNNDKGGLGDLIQQLQPTEMNKVNISDSDMSVLHQGFYQVAHGTSGLTTGRAFSNGALVSISGKTGTAESYVADGQQATNTNAVAYAPSDNPQIAVAVVFPHNTN

>43

TNVFVPGSVVKAATISSGWENGVLSGNQTLTDQPIVFQGSAPIYSWYKLAYGSFPITAVEALEYSSNAYMVQTALGIMGQTYQPNMFVGTSNLETAMGKLRATFGEYGLGAATGIDLPDESTGFVPKDYSFANYITNAFGQFDNYTPMQLAQYVATIANNGVRVAPRIVEGIYGNNDKGGLGDLIQQLQPTEMNKVNISDSDMSILHQGFYQVAHGTSGLTTGRAFSNGALVSISGKTGTAESYVADGQQATNTNAVAYAPSDNPQIAVAVVFPHNTN

>44

TNVFVPGSVVKAATISSGWENGVLSGNQTLTDQSIVFQGSAPINSWYTQAYGSFPITAVQALEYSSNTYMVQTALGLMGQTYQPNMFVGTSNLESAMEKLRSTFGEYGLGTATGIDLPDESTGFVPKEYSFANYITNAFGQFDNYTPMQLAQYVATIANNGVRVAPRIVEGIYGNNDKGGLGNLIQQLQPTEMNKVNISDSDMSILHQGFYQVAHGTSGLTTGRAFSNGALVSISGKTGTAESYVADGQQATNTNAVAYAPSDNPQIAVAVVFPHNTN

>45

TNVFVPGSVVKAATISSGWENGVLSGNQTLTDQSIVFQGSAPINSWYTQAYGSFPITAVQALEYSSNTYMVQTALGLMGQTYQPNMFVGTSNLESAMEKLRSTFGEYGLGTATGIDLPDESTGFVPKEYSFANYITNAFGQFDNYTPMQLAQYVATIANNGVRVAPRIVEGIYGNNDKGGLGDLIQQLQPTEMNKVNISDSDMSILHQGFYQVAHGTSGLTTGRAFSNGALVSISGKTGTAESYVADGQQATNTNAVTYAPSDNPQIAVAVVFPHNTN

>46

TNVFVPGSVVKAATISSGWENGVLSGNQTLTDQPIVFQGSAPIYSWYKLAYGSFPITAVEALEYSSNAYMVQTALGIMGQTYQPNMFVGTSNLETAMGKLRATFGEYGLGAATGIDLPDESTGFVPKEYSFANYITNAFGQFDNYTPMQLAQYVATIANDGVRVAPRIVEGIYGNNDKGGLGDLIQQLQPTEMNKVNISDSDMSVLHQGFYQVAHGTSGLTTGRAFSNGALVSISGKTGTAESYVADGQEATNTNAVAYAPSDNPQIAVAVVFPHNTN

>47

TNVFVPGSVVKAATISSGWENGVLSGNQTLTDQPIVFQGSAPIYSWYKLAYGSFPITAVEALEYSSNAYVVQTALGIMGQTYQPNMFVGTSNLESAMGKLRSTFGEYGLGSATGIDLPDESTGLVPKEYNFANFITNAFGQFDNYTPMQLAQYVATIANNGVRVAPRIVEGIYGNNDKGGLGDLIQQLQPTEMNKVNISDSDMSILHQGFYQVAHGTSALTTGRAFSNGAAVSISGKTGTAESYVAGGQKADNTNAVAYAPSDNPQIAVAVVFPHNTN

>48

TNVFVPGSVVKAATISSGWENGVLSGNQTLTDQSIVFQGSAPINSWYTPQAYGSFPITAVQALEYSSNSYMVQTALGLMGQTYQPNMFVGTSKLESAMGKLRSTFGEYGLGSATGIDLPDESTGFVPKDYSFANYITNAFGQFDNYTPMQLAQYVATIANDGVRVAPRIVEGIYGNNDKGGLGDLIQQLQPTEMNKVNISDSDMSVLHQGFYQVAHGTSGLTTGRAFSNGALVSISGKTGTAESYVADGQQATNTNAVAYAPSDNPQIAVAVVFPHNTN

>49

TNVFVPGSVVKAATISSGWENGVLSGNQTLTDQPIVFQGSAPIYSWYKLAYGSFPITAVEALEYSSNAYMVQTALGIMGQTYQPNMFVGTSNLETAMGKLRATFGEYGLGAATGIDLPDESTGFVPKEYSFANYITNAFGQFDNYTPMQLAQYVATIANDGVRVAPRIVEGIYGNNDKGGLGDLIQQLQPTEMNKVNISDSDMSILHQGFYQVSHGTSPLTTGRAFSDGATVSISGKTGTGESYVAGGQEANNTNAVAYAPTENPQIAVAVVFPHNTN

>50

TNVFVPGSVVKAATISSGWENGVLSGNQTLTDQSIVFQGSAPINSWYTQAYDSFPITAVQALEYSSNTYMVQTALGLMGQTYQPNMFVGTSNLESAMEKLRSTFGEYGLGTATGIDLPDESTGFVPKEYSFANYITNAFGQFDNYTPMQLAQYVATIANNGVRVAPRIVEGIYGNNDKGGLGDLIQQLQPTEMNKVNISDSDMSILHQGFYQVAHGTSELTTGRAFSNGALVSISGKTGTAESYVADGQQATNTNAVAYAPSDNPQIAVAVVFPHNTN

>51

TNVFVPGSVVKAATISSGWENGVLSGNQTLTDQPIVFQGSAPIYSWYKLAYGSFPITAVEALEYSSNAYVVQTALGIMGQTYQPNMFVGTSNLETAMGKLRATFGEYGLGAATGIDLPDESTGLVPKEYNFANFITNAFGQFDNYTPMQLAQYVATIANNGVRLAPHIVEGIYDNNDKGGLGELIQAIDTKEINKVNISESDMAILHQGFYQVSHGTSPLTTGRAFSDGATVSISGKTGTGESYVAGGQEANNTNAVAYAPTENPQIAVAVVFPHNTN

>52

TNVFVPGSVVKAATISSGWENGVLSGNQTLTDQSIVFQGSAPINSWYTAFSVPMPITAVQALEYSSNAYMVQTALGLMGQTYQPNMFVGTSNLESAMGKLRSTFGEYGLGAATGIDLPDESTGFVPKEYSFANYITNAFGQFDNYTPMQLAQYVATIANNGVRVAPRIVEGIYGNNDKGGLGDLIQQLQPTEMNKVNISDSDMSILHQGFYQVAHGTSGLTTGRAFSNGALVSISGKTGTAESYVADGQQATNTNAVAYAPSDNPQIAVAVVFPHNTN

>53

TNVFVPGSVVKAATISSGWENGVLSGNQTLTDQSIVFQGSAPINSWYTAFSVPMPITAVQALEYSSNAYMVQTALGLMGQTYQPNMFVGTSNLESAMGKLRSTFGEYGLGSATGIDLPDESTGFIPKEYSFANYITNAFGQFDNYTPMQLAQYVATIANDGVRVAPRIVEGIYGNNDKGGLGDLIQQLQPTEMNKVNISDSDMSILHQGFYQVAHGTSGLTTGRAFSNGALVSISGKTGTAESYVAGGQEANNTNAVAYAPSDNPQIAVAVVFPHNTN

>54

TNVFVPGSVVKAATISSGWENGVLSGNQTLTDQPIVFQGSAPIYSWYKLAYGSFPITAVEALEYSSNAYVVQTALGIMGQTYQPNMFVGTSNLESAMGKLRSTFGEYGLGSATGIDLPDESTGLVPKEYNFANFITNAFGQFDNYTPMQLAQYVATIANNGVRLAPHIVEGIYDNNDKGGLGELIQAIDTKEINKVNISESDMAILHQGFYQVSHGTSPFTTGRAFSDGATVSISGKTGTGESYVAGGQEANNTNAVAYAPTENPQIAVAVVFPHNTN

>55

TNVFVPGSVVKAATISSGWENGVLSGNQTLTDQSIVFQGSAPINSWYTQAYGSFPITAVQALEYSSNTYMVQTALGLMEQTYQPNMFVGTSNLESAMEKLRSTFGEYGLGTATGIDLPDESTGFVPKEYSFANYITNAFGQFDNYTPMQLAQYVATIANNGVRVAPRIVEGIYGNNDKGGLGDLIQQLQPTEMNKVNISDSDMSILHQGFYQVAHGTSGLTTGRAFSNGALVSISGKTGTAESYVADGQQATNTNAVAYAPSDNPQIAVAVVFPHNTN

>56

TNVFVPGSVVKAATISSGWENGVLSGNQTLTDQPIVFQGSAPIYSWYKLAYGSFPITAVEALEYSSNAYMVQTALGIMGQTYQPNMFVGTSNLESAMGKLRSTFGEYGLGSATGIDLPDESTGFVPKDYSFANYITNAFGQFDNYTPMQLAQYVATIANDGVRVAPRIVEGIYGNNDKGGLGELIQAIDTKEINKVNISESDMAILHQGFYQVAHGTSGLTTGRAFSNGALVSISGKTGTAESYVAGGQEANNTNAVAYAPSDNPQIAVAVVFPHNTN

>57

TNVFVPGSVVKAATISSGWENGVLSGNQTLTDQPIVFQGSAPINSWYTQAYDSFPITAVEALEYSSNAYMVQTALGLMGQTYQPNMFVGTSNLESAMGKLRSTFGEYGLGSATGIDLPDESTGFVPKEYSFANYITNAFGQFDNYTPMQLAQYVATIANDGVRVAPRIVEGIYGNNDKGGLGDLIQQLQPTEMNKVNISDSDMSILHQGFYQVAHGTSGLTTGRAFSNGAAVSISGKTGTAESYVAGGQEANNTNAVAYAPSDNPQIAVAVVFPHNTN

>58

TNVFVPGSVVKAATISSGWENGVLSGNQTLTDQSIVFQGSAPINSWYTQAYGSFPITAVQALEYSSNTYMVQTALGLMGQTYQPNMFVGTSNLESAMEKLRSTFGEYGLGTATGIDLPDESTGFVPKEYSFANYITNAFGQFDNYTPMQLAQYVATIANNGVRVAPRIVEGIYGNNDKGGLGDLIQQLQPTEMNKVNISDSDMSILHQGFYQVAHGTSGLTTGRAFSNGALVSISGKTGTGESYVAGGQEANNTNAVAYAPTENPQIAVAVVFPHNTN

>59

TNVFVPGSVVKAATISSGWENGVLSGNQTLTDRSIVFQGSAPINSWYTQAYGSFPITAVQALEYSSNTYMVQTALGLMGQTYQPNMFVGTSNLESAMEKLRSTFGEYGLGTATGIDLPDESTGFVPKEYSFANYITNAFGQFDNYTPMQLAQYVATIANNGVRVAPRIVEGIYGNNDKGGLGDLIQQLQPTEMNKVNISDSDMSILHQGFYQVAHGTSGLTTGRAFSNGALVSISGKTGTAESYVADGQQATNTNAVAYAPSDNPQIAVAVLFPHNTN

>60

TNVFVPGSVVKAATISSGWENGVLSGNQTLTDQSIVFQGSAPINSWYTQAYGSFPITAVQALEYSSNAYMVQTALGLMGQTYQPNMFVGTSNLESAMGKLRSTFGEYGLGSATGIDLPDESTGFVPKEYSFANYITNAFGQFDNYTPMQLAQYVATIANDGVRVAPRIVEGIYGNNDKGGLGDLIQQLQPTEMNKVNISDSDMSILHQGFYQVAHGTSGLTTGRAFSNGALVSISGKTGTAESYVADGQQATNTNAVAYAPSDNPQIAVAVVFPHNTN

>61

TNVFVPGSVVKAATISSGWENGVLSGNQTLKDQSIVFQGSAPINSWYPAFSEPMPITAVQALEYSSNAYMVQTALGLMGQTYQPNMFVGTSNLESAMGKLRSTFGEYGLGSATGIDLPDESTGFVPKDYSFANYITNAFGQFDNYTPMQLAQYVATIANDGVRVAPRIVEGIYGNNDKGGLGDLIQQLQPTEMNKVNISDSDMSILHQGFYQVAHGTSGLTTGRAFSNGAAVSISGKTGTAESYVAGGQEANNTNAVAYAPSDNPQIAVAVVFPHNTN

>62

TNVFVPGSVVKAATISSGWENGVLSGNQTLTDQPIVFQGSAPIYSWYKLAYGSFPITAVEALEYSSNAYMVQTALGIMGQTYQPNMFVGTSNLETAMGKLRATFGEYGLGAATGIDLPDESTGFVPKDYSFANYITNAFGQFDNYTPMQLAQYVATIANDGVRVAPRIVEGIYGNNDKGGLGDLIQQLQPTEMNKVNISDSDMSILHQGFYQVAHGTSGLTTGRAFSNGALVSISGKTGTAESYVADGQQATNTNAVAYAPSDNPQIAVAVVFPHNTN

>63

TNVFVPGSVVKAATISSGWENGVLSGNQTLTDQPIVFQGSAPIYSWYKLAYGSFPITAVEALEYSSNAYMVQTALGIMGQTYQPNMFVGTSNLETAMGKLRATFGEYGLGAATGIDLPDESTGFVPKEYSFANYITNAFGQFDNYTPMQLAQYVATIANDGVRVAPRIVEGIYGNNDKGGLGDLIQQLQPTEMNKVNISDSDMSILHQGFYQVAHGTSALTTGRAFSNGAAVSISGKTGTAESYVAGGQEANNTNAVAYAPSDNPQIAVAVVFPHNTN

>64

TNVFVPGSVVKAATISSGWENGVLSGNQTLTDQPIVFQGSAPIYSWYKLAYGSFPITAVEALEYSSNAYMVQTALGIMGQTYQPNMFVGTSNLESAMEKLRSTFGEYGLGTATGIDLPDESTGFVPKEYSFANYITNAFGQFDNYTPMQLAQYVATIANNGVRVAPRIVEGIYGNNDKGGLGDLIQQLQPTEMNKVNISDSDMSILHQGFYQVAHGTSELTTGRAFSNGALVSISGKTGTAESYVADGQQATNTNAVAYAPSDNPQIAVAVVFPHNTN

>65

TNVFVPGSVVKAATISSGWENGVLSGNQTLTDQPIVFQGSAPINSWYTQAYDSFPITAVEALEYSSNAYMVQTALGLMGQTYQPNMFVGTSNLESAMGKLRSTFGEYGLGSATGIDLPDESTGFVPKEYSFANYITNAFGQFDNYTPMQLAQYVATIANDGVRVAPRIVEGIYGNNDKGGLGDLIQQLQPTEMNKVNISDSDMSILHQGFYQVAHGTSGLTTGRAFSNGALVSISGKTGTAESYVAGGQEANNTNAVAYAPSDNPQIAVAVVFPHNTN

>66

TNVFVPGSVVKAATISSGWENGVLSGNQTLTDQPIVFQGSAPIYSWYKLAYGSFPITAVEALEYSSNAYMVQTALGIMGQTYQPNMFVGTSNLETAMGKLRATFGEYGLGAATGIDLPDESTGFVPKEYSFANYITNAFGQFDNYTPMQLAQYVATIANDGVRVAPRIVEGIYGNNDKGGLGDLIQQLQPTEINKVNISDSDMSILHQGFYQVAHGTSGLTTGRAFSNGALVSISGKTGTAESYVADGQQATNTNAVAYAPSDNPQIAVAVVFPHNTN

>67

TNVFVPGSVVKAATISSGWENGVLSGNQTLTDQSIVFQGSAPINSWYTQAYDSFPITAVEALEYSSNAYMVQTALGLMGQTYQPNMFVGTSNLESAMGKLRSTFGEYGLGSATGIDLPDESTGFVPKEYSFANYITNAFGQFDNYTPMQLAQYVATIANDGVRVAPRIVEGIYGNNDKGGLGDLIQQLQPTEMNKVNISDSDMSILHQGFYQVAHGTSGLTTGRAFSNGALVSISGKTGTAESYVAGGQEANNTNAVAYAPSDNPQIAVAVVFPHNTN

>68

TNVFVPGSVVKAATISSGWENGVLSGNQTLTDQSIVFQGSAPINSWYTQAYDSFPITAVEALEYSSNAYMVQTALGIMGQTYQPNMFVLTNNLESAMGKLRSTFAEYGLGASTGIDLPNESTGFIPKEYNFANYITNAFGQFDNYTPMQLAQYVGTIANNGVRIAPHIVEGIYGNNEQGGLGNLIQSVETKEMNKINISESDVSILQQGFYQVSHGGSALTTGRAFSNGAAVSISGKTGTAESYVEGGQKANNTNAVAYAPSDNPQIAVAVVFPHNTN

>69

TNVFVPGSVVKAATISSGWENGVLSGNQTLTDQPIVFQGSAPIYSWYKLAYGSFPITAVEALEYSSNAYMVQTALGIMGQTYQPNMFVGTSNLETAMGKLRATFGEYGLGAATGIDLPDESTGFVPKEYSFANYITNSFGQFDNYTPMQLAQYVATIANDGVRVAPRIVEGIYGNNDKGGLGDLIQQLQPTEMNKVNISDSDMSILHQGFYQVAHGTSGLTTGRAFSNGALVSISGKTGTAESYVADGQQATNTNAVAYAPSDNPQIAVAVVFPHNTN

>70

TNVFVPGSVVKAATISSGWENGVLSGNQTLTDQSIVFQGSAPINSWYTQAYGSFPIAAVQALEYSSNTYMVQTALGLMGQTYQPNMFVGTSNLESAMEKLRSTFGEYGLGTATGIDLPDESTGFVPKEYSFANYITNAFGQFDNYTPMQLAQYVATIANNGVRVAPRIVEGIYGNNDKGGLGDLIQQLQPTEMNKVNISDSDMSILHQGFYQVAHGTSGLTTGRAFSNGALVSISGKTGTAESYVADGQQATNTNAVAYAPSDNPQIAVAVVFPHNTN

>71

TNVFVPGSVVKTATISSGWENGVLSGNQTLTDQSIVFQGSAPINSWYTQAYGSFPITAVQALEYSSNTYMVQTALGLMGQTYQPNMFVGTSNLESAMEKLRSTFGEYGLGTATGIDLPDESTGFVPKEYSFANYITNAFGQFDNYTPMQLAQYVATIANNGVRVAPRIVEGIYGNNDKGGLGDLIQQLQPTEMNKVNISDSDMSILHQGFYQVAHGTSGLTTGRAFSNGALVSISGKTGTAESYVADGQQATNTNAVAYAPSDNPQIAVAVVFPHNTN

>72

TNVFVPGSVVKAATISSGWENGVLSGNQTLTDQSIVFQGSAPINSWYTQAYGSFPITAVQALEYSSNTYMVQTALGLMGQTYQPNMFVGTSNLESAMEKLRSTFGEYGLGTATGIDLPDESTGFVPKEYSFANYITNVFGQFDNYTPMQLAQYVATIANNGVRVAPRIVEGIYGNNDKGGLGDLIQQLQPTEMNKVNISDSDMSILHQGFYQVAHGTSGLTTGRAFSNGALVSISGKTGTAESYVADGQQATNTNAVAYAPSDNPQIAVAVVFPHNTN

>73

TNVFVPGSVVKAATISSGWENGVLSGNQTLTDQPIVFQGSAPIYSWYKLAYGSFPITAVEALEYSSNAYMVQTALGIMGQTYQPNMFVGTSNLETAMGKLRATFGEYGLGAATGIDLPDESTGFVPKEYSFANYITNAFGQFDNYTPMQLAQYVATIANDGVRVAPRIVEGIYGNNDKGGLGDLIQQLQPTEMNKVNISDSDMSILHQGFYQVAHGTSGLTTGRAFSNGAAVSISGKTGTAESYVEGGQEANNTNAVAYAPSDNPQIAVAVVFPHNTN
