# Supplementary material for: Strain features and distributions in pneumococci from children with invasive disease before and after 13-valent conjugate vaccine implementation in the USA
Source: Clin Microbiol Infect. 2016 Jan;22(1):60.e9–60.e29. doi: 10.1016/j.cmi.2015.08.027 (PMC4721534; doi:10.1016/j.cmi.2015.08.027)
Supplement: Table S5 — PBP2x transpeptidase domain sequences compiled from approximately 1500 ABCs isolates collected during 1998–2013 [file mmc5.docx]

sTable 5. PBP2x transpeptidase domain sequences compiled from approximately 1500 ABCs isolates collected during 1998-2013.

>0

GTDGIITYEKDRLGNIVPGTEQVSQRTMDGKDVYTTISSPLQSFMETQMDAFQEKVKGKYMTATLVSAKTGEILATTQRPTFDADTKEGITEDFVWRDILYQSNYEPGSTMKVMMLAAAIDNNTFPGGEVFNSSELKIADATIRDWDVNEGLTGGRMMTFSQGFAHSSNVGMTLLEQKMGDATWLDYLNRFKFGVPTRFGLTDEYAGQLPADNIVNIAQSSFGQGISVTQTQMIRAFTAIANDGVMLEPKFISAIYDPNDQTARKSQKEIVGNPVSKDAASLTRTNMVLVGTDPVYGTMYNHSTGKPTVTVPGQNVALKSGTAQIADEKNGGYLVGVTDYIFSAVSMSPAENPDFILYV

>1

GKDGIITYEKDRLGNIVPGTEQVSQQTVDGKDVYTTISSTLQSFMETQMNAFQEKVKGKYMTATLVSAKTGEILATTQRPTFDADTKEGLTKDFVWRDILYQSNYEPGSTMKVMTLAAAIDNNTFPGGEVFNSSELKVADATIRDWDVNEGLTGGGMMTFSQGFAHSSNVGMTLLEQKMGDATWLDYLNRFKFGIPTRFGLTDEYAGQLPADNIVNIAQSSFGQGISVTQTQMIRAFTAIANDGVMLEPKFITALYDPNNQSVRKSQKEIVGNPVSKDAASQTRTHMVLVGTDPRYGTMYNHSTGKATVNVPGQNVALKSGTAEIADEKNGGYLVGSTNNIFSVVAMNPAENPDFILYV

>2

GTDGIITYEKDRLGNIVPGTEQVSQRTMDGKDVYTTISSPLQSFMETQMDAFQEKVKGKYMTATLVSAKTGEILATTQRPTFDADTKEGITEDFVWRDILYQSNYEPGSTMKVMMLAAAIDNNTFPGGEVFNSSELKIADATIRDWDVNEGLTGGRMMTFSQGFAHSSNVGMTLLEQKMGDATWLDYLNRFKFGVPTRFGLTDEYAGQLPADNIVNIAQSSFGQGISVTQTQMIRAFTAIANDGVMLEPKFISAIYDPNDQTARKSQKEIVGNPVSKDAASLTRTNMVLVGTDPVYGTMYNHSTGKPTVTVPGQNVALKSGTAQIADEKNGGYLVGLTDYIFSAVSMSPAENPDFILYV

>3

GTDGIITYEKDRLGNIVPGTEQVSQRTMDGKDVYTTISSPLQSFMETQMDAFQEKVKGKYMTATLVSAKTGEILATTQRPTFDADTKEGITEDFVWRDILYQSNYEPGSTMKVMMLAAAIDNNTFPGGEVFNSSELKIADATIRDWDVNEGLTGGRTMTFSQGFAHSSNVGMTLLEQKMGDATWLDYLNRFKFGVPTRFGLTDEYAGQLPADNIVNIAQSSFGQGISVTQTQMIRAFTAIANDGVMLEPKFISAIYDPNDQTARKSQKEIVGNPVSKDAASLTRTNMVLVGTDPVYGTMYNHSTGKPTVTVPGQNVALKSGTAQIADEKNGGYLVGLTDYIFSAVSMSPAENPDFILYV

>4

GTDGIITYEKDRLGNIVPGTEQVSQRTMDGKDVYTTISSPLQSFMETQMDAFQEKVKGKYMTATLVSVKTGEILATTQRPTFDADTKEGITEDFVWRDILYQSNYEPGSTMKVMMLAAAIDNNTFPGGEVFNSSELKIADATIRDWDVNEGLTGGRMMTFSQGFAHSSNVGMTLLEQKMGDATWLDYLNRFKFGVPTRFGLTDEYAGQLPADNIVNIAQSSFGQGISVTQTQMIRAFTAIANDGVMLEPKFISAIYDPNDQTARKSQKEIVGNPVSKDAASLTRTNMVLVGTDPVYGTMYNHSTGKPTVTVPGQNVALKSGTAQIADEKNGGYLVGVTDYIFSAVSMSPAENPDFILYV

>5

GTDGIITYEKDRLGNIVPGTEQVSQRTMDGKDVYTTISSPLQSFMETQMDSFQEKVKGKYMTATLVSAKTGEILATTQRPTFDADTKEGITEDFVWRDILYQSNYEPGSTMKVMMLAAAIDNNTFPGGEVFNSSELKIADATIRDWDVNEGLTGGRMMTFSQGFAHSSNVGMTLLEQKMGDATWLDYLNRFKFGVPTRFGLTDEYAGQLPADNIVNIAQSSFGQGISVTQTQMIRAFTAIANDGVMLEPKFISAVYDPNDQTARKSQKEIVGNPVSKDAASLTRTNMVLVGTDPVYGTMYNHSTGKPTVTVPGQNVALKSGTAQIADEKNGGYLVGLTDYIFSAVSMSPAENPDFILYV

>6

GTDGIITYEKDRLGNIVPGTEQVSQRTMDGKDVYTTISSPLQSFMETQMDAFQEKVKGKYMTATLVSAKTGEILATTQRPTFDADTKEGITEDFVWRDILYQSNYEPGSTMKVMMLAAAIDNNTFPGGEVFNSSELKIADATIRDWDVNEGLTGGRTMTFSQGFAHSSNVGMTLLEQKMGDATWLDYLNRFKFGVPTRFGLTDEYAGQLPADNIVNIAQSSFGQGISVTQTQMIRAFTAIANDGVMLEPKFISAIYDPNDQTARKSQKEIVGNPVSKDAASLTRTNMVLVGTDPVYGTMYNHSTGKPTVTVPGQNVALKSGTAQIADEKNGGYLVGVTDYIFSAVSMSPAENPDFILYV

>7

GTDGIITYEKDRVGNIVPGTELVSQQTVDGKDVYTTLSSPLQSFMETQMDAFLEKVKGKYMTATLVSAKTGEILATTQRPTFNADTKEGITEDFVWRDILYQSNYEPGSAMKVMTLASSIDNNTFPSGEYFNSSEFKIADATTRDWDVNEGLTTGGMMTFLQGFAHSSNVGMSLLEQKMGDATWLDYLKRFKFGVPTRFGLTDEYAGQLPADNIVSIAQSSFGQGISVTQTQMLRAFTAIANDGVMLEPKFISAIYDTNNQSVRKSQKEIVGNPVSKEAASTTRNHMILVGTDPLYGTMYNHYTGKPIITVPGQNVAVKSGTAQIADEKNGGYLVGSTNYIFSVVTMNPAENPDFILYV

>8

GKDGIITYEKDRLGNIVPGTEQVSQQTVDGKDVYTTLSSPLQSFMETQMDAFLEKVKGKYMTATLVSAKTGEILATTQRPTFNADTKEGITEDFVWRDILYQSNYEPGSAMKVMTLASSIDNNTFPSGEYFNSSEFKIADATTRDWDVNEGLTTGGMMTFLQGFAHSSNVGMSLLEQKMGDATWLDYLKRFKFGVPTRFGLTDEYAGQLPADNIVSIAQSSFGQGISVTQTQMLRAFTAIANDGVMLEPKFISAIYDTNNQSVRKSQKEIVGNPVSKEAASTTRNHMILVGTDPLYGTMYNHYTGKPIITVPGQNVAVKSGTAQIADEKNGGYLVGSTNYIFSAVTMNPAENPDFILYV

>9

GKDGIITYEKDRLGNIVPGTEQVSQQTVDGKDVYTTISSTLQSFMETQMNAFQEKVKGKYMTATLVSAKTGEILATTQRPTFDADTKEGLTKDFVWRDILYQSNYEPGSAMKVMTLAAAIDNNTFPGGEVFNSSELKVADVTIRDWDVNEGLTTGGMMTFLQGFAHSSNVGMTLLEQKMGDATWLDYLNRFKFGVPTRFGLTDEYAGQLPADNIVNIAQSSFGQGISVTQTQMIRAFTAIANDGVMLEPKFISALYDPNDQSVRKSQKEIVGNPVSKDAASLTRTHMVLVGTDPVYGTMYNHKTGKPTVTVPGQNVALKSGTAQIADEKNGGYLVGLTNYIFSAVSMNPAENPDFILYV

>10

GTDGIITYEKDRLGNIVPGTEQVSQRTMDGKDVYTTISSPLQSFMETQMDAFQEKVKGKYMTATLVSAKTGEILATTQRPTFDADTKEGITEDFVWRDILYQSNYEPGSTMKVMMLAAAIDNNTFPGGEVFNSSELKIADATIRDWDVNEGLTGGRMMTFSQGFAHSSNVGMTLLEQKMGDATWLDYLNRFKFGVPTRFGLTDEYAGQLPADNIVNIAQSSFGQGISVTQTQMIRAFTAIANDGVMLEPKFLSAIYDPNDQTARKSQKEIVGNPVSKDAASLTRTNMVLVGTDPVYGTMYNHSTGKPTVTVPGQNVALKSGTAQIADEKNGGYLVGVTDYIFSAVSMSPAENPDFILYV

>11

GTDGIITYEKDRLGNIVPGTEQISQQTVDGKDVYTTISSPLQSFMETQMDAFQEKVKGKYMTATLVSAKTGEILATTQRPTFNADTKEGITKDFVWRDILYQSNYEPGSAMKVMTLAASIDNNTFPGGEYFNSSELKIADVTIRDWDVNEGLTGGGMMTFSQGFAHSSNVGMTLLEQKMGDATWLDYLNRFKFGVPTRFGLTDEYAGQLPADNIVNIAQSSFGQGISVTQTQMIRAFTAIANDGVMLEPKFISAIYDPNDQTARKSQKEIVGNPVSKDAASLTRTNMVLVGTDPVYGTMYNHSTGKPTVTVPGQNVALKSGTAQIADEKNGGYLVGLTNYIFSAVSMSPAENPDFILYV

>12

GTDGIITYEKDRLGNIVPGTEQVSQRTMDGKDVYTTISSPLQSFMETQMDAFQEKVKGKYMTATLVSAKTGEILATTQRPTFDADTKEGITEDFVWRDILYQSNYEPGSTMKVMMLAAAIDNNTFPGGEVFNSSELKIADATIRDWDVNEGLTGGRMMTFSQGFVHSSNVGMTLLEQKMGDATWLDYLNRFKFGVPTRFGLTDEYAGQLPADNIVNIAQSSFGQGISVTQTQMIRAFTAIANDGVMLEPKFISAIYDPNDQTARKSQKEIVGNPVSKDAASLTRTNMVLVGTDPVYGTMYNHSTGKPTVTVPGQNVALKSGTAQIADEKNGGYLVGLTDYIFSAVSMSPAENPDFILYV

>13

GTDGIITYEKDRLGNIVPGTEQVSQQTVDGKDVYTTISSPLQSFMETQMDAFQEKVKGKYMTATLVSAKTGEILATTQRPTFNADTKEGITKDFVWRDILYQSNYEPGSTMKVMTLASSIDNNTFPSGEYFNSSELKIADVTIRDWDVNDGLTTGRMMTFLQGFALSSNVGMTLLEQKMGDATWLDYLNRFKFGVPTRFGLTDEYAGQLPADNIVNIAQSSFGQGISVTQTQMIRAFTAIANDGVMLEPKFISAIYDPNDQTARKSQKEIVGNPVSKDAASLTRTNMVLVGTDPVYGTMYNHSTGKPIITVPGQNVAVKSGTAQIADEKNGGYLVGSTNYIFSVVTMNPAENPDFILYV

>14

GTDGIITYEKDRLGNIVPGTEQVSQRTMDGKDVYTTISSPLQSFMETQMDAFQEKVKGKYMTATLVSAKTGEILATTQRPTFDADTKEGITEDFVWRDILYQSNYEPGSTMKVMMLAAAIDNNTFPGGEVFNSSELKIADATIRDWDVNEGLTGGRMMTFSQGFAHSSNVGMTLLEQKMGDATWLDYLNRFKFGVPTRFGLTDEYTGQLPADNIVNIAMSAFGQGISVTQTQMLRAFTAIANDGVMLEPKFISALYDPNDQSVRKSQKEVVGNPVSKEAASVTRDHMVMVGTDPTYGTMYNHSTGKATVNVPGQNVALKSGTAEIADEKNGGYLTGSTNNIFSVVSMHPAENPDFILYV

>15

GTDGIITYEKDRLGNIVPGTEQVSQRTMDGKDVYTTISSPLQSFMETQMDAFQEKVKGKYMTATLVSAKTGEILATTQRPTFDADTKEGITEDFVWRDILYQSNYEPGSTMKVIMLAAAIDNNTFPGGEVFNSSELKIADATIRDWDVNEGLTGGRMMTFSQGFAHSSNVGMTLLEQKMGDATWLDYLNRFKFGVPTRFGLTDEYAGQLPADNIVNIAQSSFGQGISVTQTQMIRAFTAIANDGVMLEPKFISAIYDPNDQTARKSQKEIVGNPVSKDAASLTRTNMVLVGTDPVYGTMYNHSTGKPTVTVPGQNVALKSGTAQIADEKNGGYLVGVTDYIFSAVSMSPAENPDFILYV

>16

GTDGIITYEKDRVGNIVPGTELVSQQTVDGKDVYTTLSSPLQSFMETQMDAFLEKVKGKYMTATLVSAKTGEILATTQRPTFNADTKEGITEDFVWRDILYQSNYEPGSAMKVMTLASSIDNNTFPSGEYFNSSEFKIADATTRDWDVNEGLTTGGMMTFLQGFAHSSNVGMSLLEQKMGDATWLDYLKRFKFGVPTRFGLTDEYAGQLPADNIVSIAQSSFGQGISVTQTQMLRAFTAIANDGVMLEPKFISAIYDTNNQSVRKSQKEIVGKPVSEDTASLTRTNMILVGTDPLYGTMYNHYTGKPIITVPGQNVAVKSGTAQIADEKNGGYLVGSTNYIFSAVTMNPAENPDFILYV

>17

GTDGIITYEKDRLGNIVPGTEQVSQRTMDGKDVYTTISSPLQSFMETQMDAFQEKVKGKYMTATLVSAKTGEILATTQRPTFDADTKEGITEDFVWRDILYQSNYEPGSTMKVMMLAAAIDNNTFPGGEVFNSSELKIADATIRDWDVNEGLTGGRMMTFSQGFAHSSNVGMTLLEQKMGDATWLDYLNRFKFGVPTRFGLTDEYAGQLPADNIVNIAQSSFGQGISVTQTQMIRAFTAIANDGVMLEPKFISAIYDPNDQTARKSQKEIVGNPVSKDAASLTRTNMVLVGTDPVYGTMYNHITGKPTVTVPGQNVALKSGTAQIADEKNGGYLVGLTDYIFSAVSMSPAENPDFILYV

>18

GTDGIITYEKDRVGNIVPGTELVSQQTVDGKDVYTTLSSPLQSFMETQMDAFLEKVKGKYMTATLVSAKTGEILATTQRPTFNADTKEGITEDFVWRDILYQSNYEPGSAMKVMTLASSIDNNTFPSGEYFNSSEFKIADATTRDWDVNDGLTTGGMMTFLQGFAHSSNVGMSLLEQKMGDATWLDYLKRFKFGVPTRFGLTDEYAGQLPADNIVSIAQSSFGQGISVTQTQMLRAFTAIANDGVMLEPKFISAIYDTNNQSVRKSQKEIVGNPVSKEAASTTRNHMILVGTDPLYGTMYNHYTGKPIITVPGQNVAVKSGTAQIADEKNGGYLVGSTNYIFSVVTMNPAENPDFILYV

>19

GTDGIITYEKDRLGNIVPGTEQVSQQTVDGKDVYTTISSPLQSFMETQMDAFQEKVKGKYMTATLVSAKTGEILATTQRPTFDADTKEGLTKDFVWRDILYQSNYEPGSTMKVMTLAAAIDNNTFPGGEVFNSSELKIADVTIRDWDVNDGLTTGRMMTFLQGFALSSNVGMTLLEQKMGDATWLDYLNRFKFGVPTRFGLTDEYAGQLPADNIVNIAQSSFGQGISVTQTQMIRAFTAIANDGVMLEPKFISAIYDPNDQTARKSQKEIVGNPVSKDAASLTRTNMILVGTDPVYGTMYNHSTGKPTVTVPGQNVALKSGTAQIADEKNGGYLVGLTNYIFSAVSMNPAENPDFILYV

>20

GTDGIITYEKDRLGNIVPGTEQVSQQTVDGKDVYTTLSSPLQSFMETQMDAFLEKVKGKYMTATLVSAKTGEILATTQRPTFNADTKEGITEDFVWRDILYQSNYEPGSAMKVMTLASSIDNNTFPSGEYFNSSEFKIADATTRDWDVNEGLTTGGMMTFLQGFAHSSNVGMSLLEQKMGDATWLDYLKRFKFGVPTRFGLTDEYAGQLPADNIVSIAQSSFGQGISVTQTQMLRAFTAIANDGVMLEPKFISAIYDTNNQSVRKSQKEIVGNPVSKEAASTTRNHMILVGTDPLYGTMYNHYTGKPIITVPGQNVAVKSGTAQIADEKNGGYLVGSTNYIFSAVTMNPAENPDFILYV

>21

GTDGIITYEKDRLGNIVPGTEQVSQRTMDGKDVYTTISSPLQSFMETQMDAFQEKVKGKYMTATLVSAKTGEILATTQRPTFDADTKEGITEDFVWRDILYQSNYEPGSTMKVMMLAAAIDNNTFPGGEVFNSSELKIADATIRDWDVNEGLTGGRMMTFSQGFAHSSNVGMTLLEQKMGDATWLDYLNRFKFGVPTRFGLTDEYAGQLPADNIVNIAQSSFGQGISVTQTQMIRAFTAIANDGVMLEPKFISAIYDPNDQTARKSQKEIVGNPVSKDAASLTRTNMVLVGTDPVYGTMYNHSTGKPTVTVPGQNVALKSGTAEIADEKNGGYLVGSTNNIFSVVAMNPAENPDFILYV

>22

GTDGIITYEKDRLGNIVPGTEQVSQRTMDGKDVYTTISSPLQSFMETQMDAFQEKVKGKYMTATLVSAKTGEILATTQRPTFDADTKEGITEDFVWRDILYQSNYEPGSTMKVMMLAAAIDNNTFPSGEYFNSSELKIADATIRDWDVNDGLTTGGMMTFLQGFAHSSNVGMSLLEQKMGDATWLDYLNRFKFGVPTRFGLTDEYAGQLPADNIVNIAMSAFGQGISVTQTQMLRAFTAIANDGVMLEPKFISALYDPNDQTARKSQKEIVGNPVSKDAASLTRTNMILVGTDPVYGTMYNHSTGKPTVTVPGQNVALKSGTAEIADEKNGGYLVGSTNNIFSVVAMNPAENPDFILYV

>23

GTDGIITYEKDRLGNIVPGTEQVSQRTMDGKDVYTTISSPLQSFMETQMDAFQEKVKGKYMTATLVSAKTGEILATTQRPTFDADTKEGITEDFVWRDILYQSNYEPGSTMKVMMLAAAIDNNTFPGGEVFNSSELKIADATIRDWDVNEGLTGGRMMTFSQGFAHSSNVGMTLLEQKMGDATWLDYLNRFKFGVPTRFGLTDEYAGQLPADNIVNIAQSSFGQGISVTQTQMIRAFTAIANDGVMLEPKFISAIYDPNNQTARKSQKEIVGNPVSKDAASLTRTNMVLVGTDPVYGTMYNHSTGKPTVTVPGQNVALKSGTAQIADEKNGGYLVGVTDYIFSAVSMSPAENPDFILYV

>24

GTDGIITYEKDRLGNIVPGTEQVSQQTVDGKDVYTTISSTLQSFMETQMNAFQEKVKGKYMTATLVSAKTGEILATTQRPTFDADTKEGLTKDFVWRDILYQSNYEPGSTMKVMTLAASIDNNTFPGGEYFNSSELKIADVTIRDWDVNDGLTTGRMMTFLQGFALSSNVGMSLLEQKMGDATWLDYLNRFKFGVPTRFGLTDEYAGQLPADNIVNIAQSSFGQGISVTQTQMLRAFTAIANDGVMLEPKFISAIYDTNNQSVRKSQKEIVGKPVSEDAASLTRTNMILVGTDPLYGTMYNHQTGKPIITVPGQNVAVKSGTAQIADEKNGGYLVGSTNYIFSVVTMNPAENPDFILYV

>25

GTDGIITYEKDRLGNIVPGTEQVSQQTVDGKDVYTTISSPLQSFMETQMDAFQEKVKGKYMTATLVSAKTGEILATTQRPTFDADTKEGITEDFVWRDILYQSNYEPGSAMKVMMLAAAIDNNTFPGGEVFNSSELKIADVTIRDWDVNEGLTGGRMMTFSQGFAHSSNVGMTLLEQKMGDATWLDYLNRFKFGVPTRFGLTDEYAGQLPADNIVNIAQSSFGQGISVTQTQMIRAFTAIANDGVMLEPKFITALYDPNNQTVRRSQKEIVGNPVSKDAAGQTRTHMVLVGTDPRYGTMYNHSTGKATVNVPGQNVALKSGTAEIADEKNGGYLVGSTNNIFSVVAMNPAENPDFILYV

>26

GTDGIITYEKDRVGNIVPGTELVSQQTVDGKDVYTTLSSPLQSFMETQMDAFLEKVKGKYMTATLVSAKTGEILATTQRPTFNADTKEGITEDFVWRDILYQSNYEPGSAFKVMMLASSIDNNTFPSGEYFNSSEFKIADATTRDWDVNAGLTTGGMMTFLQGFAHSSNVGTSLLEQKMGDATWLDYLKRFKFGVPTRFGLTDEYAGQLPADNIVSIAQSSFGQGISVTQTQMLRAFTAIANDGVMLEPKFISAIYDTNNQSVRKSQKEIVGNPVSKEAASTTRNHMILVGTDPLYGTMYNHYTGKPIITVPGQNVAVKSGTAQIADEKNGGYLVGSTNYIFSVVTMNPAENPDFILYV

>27

GTDGIITYEKDRLGNIVPGTEQVSQRTMDGKDVYTTISSPLQSFMETQMDAFQEKVKGKYMTATLVSAKTGEILATTQRPTFDADTKEGITEDFVWRDILYQSNYEPGSTMKVMMLAAAIDNNTFPGGEVFNSSELKIADATIRDWDVNEGLTGGRMMTFSQGFAHSSNVGMTLLEQKMGDATWLDYLNRFKFGVPTRFGLTDEYAGQLPADNIVNIAQSSFGQGISVTQTQMIRAFTAIANDGVMLEPKFISAIYDPNDQTARKSQKEIVGNPVSKDAASLTRTNMVLVGTDPVYGTMYNHSTGKPTVTVPGQNVALKSGTAQIADEKNGGYIVGLTDYIFSAVSMSPAENPDFILYV

>28

GTDGIITYEKDRLGNIVPGTEQVSQQTVDGKDVYTTISSPLQSFMETQMDAFQEKVKGKYMTATLVSAKTGEILATTQRPTFDADTKEGITEDFVWRDILYQSNYEPGSTMKVMMLAAAIDNNTFPGGEVFNSSELKIADATIRDWDVNEGLTGGRMMTFSQGFAHSSNVGMTLLEQKMGDATWLDYLNRFKFGVPTRFGLTDEYAGQLPADNIVNIAMSAFGQGISVTQTQMLRAFTAIANDGVMLEPKFISALYDPNDQSVRKSQKEIVGNPVSKEAASVTRDHMVMVGTDPTYGTMYNHSTGKATVNVPGQNVALKSGTAEIADEKNGGYLTGSTNNIFSVVSMHPAENPDFILYV

>29

GKDGIITYEKDRLGNIVPGTEQVSQQTVDGKDVYTTISSTLQSFMETQMNAFQEKVKGKYMTATLVSAKTGEILATTQRPTFDADTKEGLTKDFVWRDILYQSNYEPGSTMKVMTLAAAIDNNTFPGGEVFNSSELKIADVTIRDWDVNEGLTGGRMMTFSQGFALSSNVGMTLLEQKMGDATWLDYLNRFKFGVPTRFGLTDEYAGQLPADNIVNIAQSSFGQGISVTQTQMLRAFTAIANDGVMLEPKFISAIYDTNNQSVRKSQKEIVGKPVSEDAASLTRTNMILVGTDPLYGTMYNHQTGKPIITVPGQNVAVKSGTAQIADEKNGGYLVGSTNYIFSVVTMNPAENPDFILYV

>30

GTDGIITYEKDRLGNIVPGTEQVSQRTMDGKDVYTTISSPLQSFMETQMDSFQEKVKGKYMTATLVSAKTGEILATTQRPTFDADTKEGLTKDFVWRDILYQSNYEPGSTMKVMTLAAAIDNNTFPGGEVFNSSELKVADATIRDWDVNEGLTGGGMMTFSQGFAHSSNVGMTLLEQKMGDATWLDYLNRFKFGIPTRFGLTDEYAGQLPADNIVNIAQSSFGQGISVTQTQMIRAFTAIANDGVMLEPKFITALYDPNNQSVRKSQKEIVGNPVSKDAASQTRTHMVLVGTDPRYGTMYNHSTGKATVNVPGQNVALKSGTAEIADEKNGGYLVGSTNNIFSVVAMNPAENPDFILYV

>31

GTDGIITYEKDRLGNIVPGTEQVSQRTMDGKDVYTTISSPLQSFMETQMDAFQEKVKGKYMTATLVSAKTGEILATTQRPTFDADTKEGITEDFVWRDILYQSNYEPGSTMKVMMLAAAIDNNTFPGGEVFNSSELKIADATIRDWDVNEGLTGGRMMTFSQGFAHSSNVGMTLLEQKMGDATWLDYLNRFKFGVPTRFGLTDEYAGQLPADNIVNIAQSSFGQGISVTQTQMIRAFTAIANDGVMLEPKFISAIYDPNDQTARKSQKEIVGNPVSKDAASLTRTNMVLVGTDPVYGTMYNHSTGKPTVTVPGRNVALKSGTAQIADEKNGGYLVGLTDYIFSAVSMSPAENPDFILYV

>32

GTDGIITYEKDRLGNIVPGTEQVSQQTVDGKDVYTTISSPLQSFMETQMDAFQEKVKGKYMTATLVSAKTGEILATTQRPTFDADTKEGITEDFVWRDILYQSNYEPGSTMKVMMLAAAIDNNTFPGGEVFNSSELKIADATIRDWDVNEGLTGGRMMTFSQGFAHSSNVGMTLLEQKMGDATWLDYLNRFKFGVPTRFGLTDEYAGQLPADNIVNIAQSSFGQGISVTQTQMIRAFTAIVNDGVMLEPKFISAIYDPNDQTARKSQKEIVGNPVSKDAASLTRTNMILVGTDPVYGTMYNHSTGKPTVTVPGQNVALKSGTAEIADEKNGGYLVGSTNNIFSVVAMNPAENPDFILYV

>33

GKDGIITYEKDRLGNIVPGTEQVSQQTVDGKDVYTTLSSPLQSFMETQMDAFLEKVKGKYMTATLVSAKTGEILATTQRPTFNADTKEGITEDFVWRDILYQSNYEPGSAFKVMMLASSIDNNTFPSGEYFNSSEFKIADATTRDWDVNAGLTTGGMMTFLQGFAHSSNVGTSLLEQKMGDATWLDYLKRFKFGVPTRFGLTDEYAGQLPADNIVSIAQSSFGQGISVTQTQMLRAFTAIANDGVMLEPKFISAIYDTNNQSVRKSQKEIVGNPVSKEAASTTRNHMILVGTDPLYGTMYNHYTGKPIITVPGQNVAVKSGTAQIADEKNGGYLVGSTNYIFSVVTMNPAENPDFILYV

>34

GTDGIITYEKDRLGNIVPGTEQVSQRTMDGKDVYTTISSPLQSFMETQMDAFQEKVKGKYMTATLVSAKTGEILATTQRPTFDADTKEGITEDFVWRDILYQSNYEPGSTMKVMMLAAAIDNNTFPGGEVFNSSELKIADATIRDWDVNEGLTGGRMMTFSQGFAHSSNVGMTLLEQKMGDATWLDYLNRFKFGVPTRFGLTDEYAGQLPADNIVNIAQSSFGQGISVTQTQMIRAFTAIANDGVMLEPKFISAIYDPNDQTARKSQKEIVGNPVSKDAASLTRTNMVLVGTDPVYGTMYNHSTGKPTVTVPGQNVALKSGTAQIADEKNGGYLVGLTDYIFSAVSMSPTENPDFILYV

>35

GTDGIITYEKDRLGNIVPGTEQVSQQTVDGKDVYTTISSPLQSFMETQMDAFQEKVKGKYMTATLVSAKTGEILATTQRPTFDADTKEGITEDFVWRDILYQSNYEPGSTMKVMMLAAAIDNNTFPSGEYFNSSELKIADVTIRDWDVNDGLTTGRMMTFLQGFALSSNVGMSLLEQKMGDTTWLDYLNRFKFGVPTRFGLTDEYAGQLPADNIVNIAQSSFGQGISVTQTQMIRAFTAIANDGVMLEPKFISAIYDPNDQTARKSQKEIVGNPVSKDAASLTRTNMILVGTDPVYGTMYNHSTGKPTVTVPGQNVALKSGTAQIADEKNGGYLVGLTNYIFSAVSMNPAENPDFILYV

>36

GTDGIITYEKDRVGNIVPGTELVSQQTVDGKDVYTTLSSPLQSFMETQMDAFLEKVKGKYMTATLVSAKTGEILATTQRPTFNADTKEGITEDFVWRDILYQSNYEPGSAMKVMTLASSIDNNTFPSGEYFNSSEFKIADATTRDWDVNEGLTTGGMMTFLQGFAHSSNVGMSLLEQKMGDATWLDYLKRFKFGVPTRFGLTDEYAGQLPADNIVSIAQSSFGQGISVTQTQMLRAFTAIANDGVMLEPKFISAIYDTNNQSVRKSQKEIVGNPVSKEAASTTRNHMILVGTDPLYGTMYNHYTGKPIITVPGQNVAVKSGTAQIADEKNGGYLVGSTNYIFSAVTMNPAENPDFILYV

>37

GTDGIITYEKDRLGNIVPGTEQVSQQTVDGKDVYTTISSPLQSFMETQMDAFLEKVKGKYMTATLVSAKTGEILATTQRPTFNADTKEGITEDFVWRDILYQSNYEPGSAFKVMMLASSIDNNTFPSGEYFNSSEFKIADATTRDWDVNAGLTTGGMMTFLQGFAHSSNVGTSLLEQKMGDATWLDYLKRFKFGVPTRFGLTDEYAGQLPADNIVSIAQSSFGQGISVTQTQMLRAFTAIANDGVMLEPKFISAIYDTNNQSVRKSQKEIVGNPVSKEAASTTRNHMILVGTDPLYGTMYNHYTGKPIITVPGQNVAVKSGTAQIADEKNGGYLVGSTNYIFSVVTMNPAENPDFILYV

>38

GTDGIITYEKDRLGNIVPGTEQVSQRTMDGKDVYTTISSPLQSFMETQMDAFQEKVKGKYMTATLVSAKTGEILATTQRPTFDADTKEGITEDFVWRDILYQSNYEPGSTMKVMMLAAAIDNNTFPGGEVFNSSELKIADATIRDWDVNEGLTGGRMMTFSQGFAHSSNVGITLLEQKMGDATWLDYLNRFKFGVPTRFGLTDEYAGQLPADNIVNIAQSSFGQGISVTQTQMIRAFTAIANDGVMLEPKFISAIYDPNDQTARKSQKEIVGNPVSKDAASLTRTNMVLVGTDPVYGTMYNHSTGKPTVTVPGQNVALKSGTAQIADEKNGGYLVGVTDYIFSAVSMSPAENPDFILYV

>39

GTDGIITYEKDRLGNIVPGTEQVSQRTMDGKDVYTTISSPLQSFMETQMDAFQEKVKGKYMTATLVSAKTGEILATTQRPTFDADTKEGITEDFVWRDILYQSNYEPGSTMKVMMLAAAIDNNTFPGGEVFNSSELKIADATIRDWDVNEGLTGGRMMTFSQGFAHSSNVGMTLLEQKMGDATWLDYLNRFKFGVPTRFGLTDEYAGQLPADNIVNIAQSSFGQGISVTQTQMIRAFTAIANDGVMLEPKFISAIYDPNDQTARKSQKEIVGNPVSKDAASLTRTNMVLVGTDPVYGTMYNHSTGKPTVTVPGQNVALKSGTAQIADEKNGGYLVGLTDYIFSAVSMNPAENPDFILYV

>40

GKDGIITYEKDRLGNIVPGTEQVSQQTVDGKDVYTTISSTLQSFMETQMNAFQEKVKGKYMTATLVSAKTGEILATTQRPTFDADTKEGLTKDFVWRDILYQSNYEPGSTMKVMTLASSIDNNTFPSGEYFNSSELKIADVTIRDWDVNDGLTTGRMMTFLQGFALSSNVGMSLLEQKMGDTTWLDYLNRFKFGVPTRFGLTDEYAGQLPADNIVNIAQSSFGQGISVTQTQMLRAFTAIANDGVMLEPKFISAIYDTNNQSVRKSQKEIVGKPVSEDAASLTRTNMILVGTDPIYGTMYNHYTGKPIITVPGQNVAVKSGTAQIADEKNGGYLVGSTNYIFSVVTMNPAENPDFILYV

>41

GTDGIITYEKDRLGNIVPGTEQVSQRTMDGKDVYTTISSPLQSFMETQMDAFQEKVKGKYMTATLVSAKTGEILATTQRPTFDADTKEGITEDFVWRDILYQSNYEPGSTMKVMMLAAAIDNNTFPGGEVFNSSELKIADATIRDWDVNEGLTGGRMMTFSQGFAHSSNVGMTLLEQKMGDATWLDYLNRFKFGVPTRFGLTDEYAGQLPADNIVNIAQSSFGQGISVTQTQMIRAFTAIANDGVMLEPKFISAIYDPNDQTARKSQKEIVGNPVSKDAASLTRTNMVLIGTDPVYGTMYNHSTGKPTVTVPGQNVALKSGTAQIADEKNGGYLVGVTDYIFSAVSMSPAENPDFILYV

>42

GTDGIITYEKDRLGNIVPGTEQVSQRTMDGKDVYTTISSPLQSFMETQMDAFQEKVKGKDMTATLVSAKTGEILATTQRPTFDADTKEGITEDFVWRDILYQSNYEPGSTMKVMMLAAAIDNNTFPGGEVFNSSELKIADATIRDWDVNEGLTGGRMMTFSQGFAHSSNVGMTLLEQKMGDATWLDYLNRFKFGVPTRFGLTDEYAGQLPADNIVNIAQSSFGQGISVTQTQMIRAFTAIANDGVMLEPKFISAIYDPNDQTARKSQKEIVGNPVSKDAASLTRTNMVLVGTDPVYGTMYNHSTGKPTVTVPGQNVALKSGTAQIADEKNGGYLVGLTDYIFSAVSMSPAENPDFILYV

>43

GTDGIITYEKDRLGNIVPGTEQVSQQTVDGKDVYTTISSTLQSFMETQMDAFLEKVKGKYMTATLVSAKTGEILATTQRPTFNADTKEGITEDFVWRDILYQSNYEPGSAMKVMTLASSIDNNTFPSGEYFNSSEFKIADATTRDWDVNEGLTTGGMMTFLQGFAHSSNVGMSLLEQKMGDATWLDYLKRFKFGVPTRFGLTDEYAGQLPADNIVSIAQSSFGQGISVTQTQMLRAFTAIANDGVMLEPKFISAIYDTNNQSVRKSQKEIVGNPVSKEAASTTRNHMILVGTDPLYGTMYNHYTGKPIITVPGQNVAVKSGTAQIADEKNGGYLVGSTNYIFSAVTMNPAENPDFILYV

>44

GTDGIITYEKDRLGNIVPGTEQVSQQTVDGKDVYTTISSTLQSFMETQMNAFQEKVKGKYMTATLVSAKTGEILATTQRPTFDADTKEGLTKDFVWRDILYQSNYEPGSTMKVMTLAAAIDNNTFPGGEVFNSSELKIADATIRDWDVNDGLTTGGMMTFLQGFAHSSNVGMSLLEQKMGDATWLDYLNRFKFGVPTRFGLTDEYSGQLPADNIVNIAMSAFGQGISVTQTQMIRAFTAIANDGVMLEPKFISAIYDPNDQTARKSQKEIVGNPVSKDAASLTRTNMILVGTDPVYGTMYNHSTGKPTVTVPGQNVALKSGTAQIADEKNGGYLVGLTNYIFSAVSMNPAENPDFILYV

>45

GTDGIITYEKDRLGNIVPGTEQISQQTVDGKDVYTTISSPLQSFMETQMDAFQEKVKGKYMTATLVSAKTGEILATTQRPTFDADTKEGITKDFVWRDILYQSNYEPGSTMKVMMLAAAIDNNTFPGGEVFDSSELKIADATIRDWDVNEGLTTGGMMTFLQGFAYSSNVGMSLLEQKMGDATWLDYLNRFKFGVPTRFGLTDEYSGQLPADNIVNIAMSAFGQGISVTQTQMLRAFTAIANDGVMLEPKFISALYDPNDQTVRKSQKEIVGNPVSKDAASQTRTHMVLVGTDPRYGTMYNHSTGKATVNVPGQNVALKSGTAEIADEKNGGYLVGTTNYIFSAVSMNPAENPDFILYV

>46

GTDGIITYEKDRLGNIVPGTEQVSQQTVDGKDVYTTISSTLQSFMETQMNAFQEKVKGKYMTATLVSAKTGEILATTQRPTFDADTKEGLTKDFVWRDILYQSNYEPGSAMKVMTLASSIDNNTFPSGEYFNSSEFKIADATTRDWDVNEGLTTGGMMTFLQGFAHSSNVGMSLLEQKMGDATWLDYLNRFKFGVPTRFGLTDEYAGQLPADNIVSIAQSSFGQGISVTQTQMLRAFTAIANDGVMLEPKFISAIYDTNNQSVRKSQKEIVGNPVSKEAASTTRNHMILVGTDPLYGTMYNHYTGKPIITVPGQNVAVKSGTAQIADEKNGGYLVGSTNYIFSAVTMNPAENPDFILYV

>47

GKDGIITYEKDRLGNIVPGTEQVSQQTVDGKDVYTTLSSPLQSFMETQMDAFLEKVKGKYMTATLVSAKTGEILATTQRPTFNADTKEGITEDFVWRDILYQSNYEPGSAMKVMTLASSIDNNTFPSGEYFNSSEFKIADATTRDWDVNDGLTTGGMMTFLQGFAHSSNVGMSLLEQKMGDATWLDYLKRFKFGVPTRFGLTDEYAGQLPADNIVSIAQSSFGQGISVTQTQMLRAFTAIANDGVMLEPKFISAIYDTNNQSVRKSQKEIVGNPVSKEAASTTRNHMILVGTDPLYGTMYNHYTGKPIITVPGQNVAVKSGTAQIADEKNGGYLVGSTNYIFSVVTMNPAENPDFILYV

>48

GTDGIITYEKDRLGNIVPGTEQVSQRTMDGKDVYTTISSPLQSFMETQMDAFQEKVKGKYMTATLVSAKTGEILATTQRPTFDADTKEGITEDFVWRDILYQSNYEPGSTMKVMMLAAAIDNNTFPGGEVFNSSELKIADATIRDWDVNEGLTTGGMMTFLQGFAHSSNVGMSLLEQKMGDATWLDYLKRFKFGVPTRFGLTDEYAGQLPADNIVSIAQSSFGQGISVTQTQMLRAFTAIANDGVMLEPKFISAIYDTNNQSVRKSQKEIVGNPVSKEAASTTRNHMILVGTDPLYGTMYNHYTGKPIITVPGQNVAVKSGTAQIADEKNGGYLVGSTNYIFSVVTMNPAENPDFILYV

>49

GTDGIITYEKDRLGNIVPGTEQVSQRTMDGKDVYTTISSPLQSFMETQMDAFQEKVKGKYMTATLVSAKTGEILATTQRPTFDADTKEGITEDFVWRDILYQSNYEPGSTMKVMMLAAAIDNNTFPGGEVFNSSELKIADATIRDWDVNEGLTGGRMMTFSQGFAHSSNVGMTLLEQKMGDATWLDYLNRFKFGVPTRFGLTDEYAGQLPADNIVNIAQSSFGQGISVTQTQMIRAFTAIANDGVMLEPKFISAIYDPNDQTARKSQKEIVGNPVSKDAASLTRTNMVLVGTDPVYGIMYNHSTGKPTVTVPGQNVALKSGTAQIADEKNGGYLVGLTDYIFSAVSMSPAENPDFILYV

>50

GTDGIITYEKDRLGNIVPGTEQVSQQTVDGKDVYTTISSTLQSFMETQMNAFQEKVKGKYMTATLVSAKTGEILATTQRPTFDADTKEGLTKDFVWRDILYQSNYEPGSTMKVMTLAAAIDNNTFPGGEVFNSSELKIADATIRDWDVNDGLTTGGMMTFLQGFAHSSNVGMSLLEQKMGDTTWLDYLNRFKFGVPTRFGLTDEYAGQLPADNIVNIAMSAFGQGISVTQTQMIRAFTAIANDGVMLEPKFISAIYDPNDQTARKSQKEIVGNPVSKDAASLTRTNMVLVGTDPVYGTMYNHSTGKPTVTVPGQNVALKSGTAQIADEKNGGYLVGLTDYIFSAVSMSPAENPDFILYV

>51

GTDGIITYEKDRVGNIVPGTELVSQQTVDGKDVYTTLSSPLQSFMETQMDAFLEKVKGKYMTATLVSAKTGEILATTQRPTFNADTKEGITEDFVWRDILYQSNYEPGSAFKVMTLASSIDNNTFPSGEYFNSSEFKIADATTRDWDVNEGLTTGGMMTFSQGFAHSSNVGTSLLEQKMGDATWLDYLNRFKFGVPTRFGLTDEYAGQLPADNIVSIAQSSFGQGISVTQTQMLRAFTAIANDGVMLEPKFISAIYDTNNQSVRKSQKEIVGNPVSKEAASTTRNHMILVGTDPLYGTMYNHYTGKPIITVPGQNVAVKSGTAQIADEKNGGYLVGSTNYIFSVVTMNPAENPDFILYV

>52

GTDGIITYEKDRLGNIVPGTELVSQQTVDGKDVYTTLSSPLQSFMETQMDAFLEKVKGKYMTATLVSAKTGEILATTQRPTFNADTKEGITEDFVWRDILYQSNYEPGSAFKVMMLASSIDNNTFPSGEYFNSSEFKIADATTRDWDVNEGLTTGGMMTFSQGFAHSSNVGTSLLEQKMGDATWLDYLKRFKFGVPTRFGLTDEYAGQLPADNIVSIAQSSFGQGISVTQTQMLRAFTAIANDGVMLEPKFISAIYDTNNQSVRKSQKEIVGNPVSKEAASTTRNHMILVGTDPLYGTMYNHYTGKPIITVPGQNVAVKSGTAQIADEKNGGYLVGSTNYIFSVVTMNPAENPDFILYV

>53

GTDGIITYEKDRLGNIVPGTEQVSQRTMDGKDVYTTISSPLQSFMETQMDAFQEKVKGKYMTATLVSAKTGEILATTQRPTFDADTKEGITEDFVWRDILYQSNYEPGSTMKVMMLAAAIDNNTFPGGEVFNSSELKIADATIRDWDVNEGLTGGRTMTFSQGFAHSSNVGMTLLEQKMGDATWLDYLNRFKFGVPTRFGLTDEYAGQLPADNIVNIAQSSFGQGISVTQTQMIRAFTAIANDGVMLEPKFISAIYDPNDQTARKSQKEIVGNPVSKDAASLTRTNMVLVGTDPVYGTMYNHSTGKPTVTVPGQNVALKSGTAQIADEKNGGYLVGLTDYIFSVVSMSPAENPDFILYV

>54

GTDGIITYEKDRLGNIIPGTEQVSQQTVDGKDVYTTISSPLQSFMETQMDAFQEKVKGKYMTATLVSAKTGEILATTQRPTFDADTKEGITEDFVWRDILYQSNYEPGSTMKVMMLAAAIDNNTFPGVEVFNSSELKIADATIRDWDVNEGLTGGRMMTFSQGFAHSSNVGMTLLEQKMGDATWLDYLNRFKFGVPTRFGLTDEYAGQLPADNIVNIAQSSFGQGISVTQTQMLRAFTAIANDGVMLEPKFISAIYDPNYQTARKSQKEIVGNPVSKDAASLTRTNMILVGTDSVYGTMYNHSTGKPTVTVPGQNVALKSGTAEIADEKNGGYLVGSTNNIFSVVAMNPAENPDFILYV

>55

GTDGIITYEKDRLGNIVPGTELVSQQTVDGKDVYTTLSSPLQSFMETQMDAFLEKVKGKYMTATLVSAKTGEILATTQRPTFNADTKEGITEDFVWRDILYQSNYEPGSAMKVMTLASSIDNNTFPSGEYFNSSEFKIADATTRDWDVNEGLTTGGMMTFLQGFAHSSNVGMSLLEQKMGDATWLDYLKRFKFGVPTRFGLTDEYAGQLPADNIVSIAQSSFGQGISVTQTQMLRAFTAIANDGVMLEPKFISAIYDTNNQSVRKSQKEIVGNPVSKEAASTTRNHMILVGTDPLYGTMYNHYTGKPIITVPGQNVAVKSGTAQIADEKNGGYLVGSTNYIFSVVTMNPAENPDFILYV

>56

GTDGIITYEKDRLGNIVPGTEQVSQQTVDGKDVYTTISSTLQSFMETQMDAFLEKVKGKYMTATLVSAKTGEILATTQRPTFNADTKEGITEDFVWRDILYQSNYEPGSAMKVMTLASSIDNNTFPSGEYFNSSEFKIADATTRDWDVNDGLTTGGMMTFLQGFAHSSNVGMSLLEQKMGDATWLDYLKRFKFGVPTRFGLTDEYAGQLPADNIVSIAQSSFGQGISVTQTQMLRAFTAIANDGVMLEPKFISAIYDTNNQSVRKSQKEIVGNPVSKEAASTTRNHMILVGTDPLYGTMYNHYTGKPIITVPGQNVAVKSGTAQIADEKNGGYLVGSTNYIFSVVTMNPAENPDFILYV

>57

GTDGIITYEKDRLGNIVPGTEQVSQRTMDGKDVYTTISSPLQSFMETQMDAFQEKVKGKYMTATLVSAKTGEILATTQRPTFDADTKEGLTKDFVWRDILYQSNYEPGSTMKVMTLAAAIDNNTFPGGEVFNSSELKIADVTIRDWDVNDGLTTGRMMTFLQGFALSSNVGMSLLEQKMGDTTWLDYLNRFKFGVPTRFGLTDEYAGQLPADNIVNIAQSSFGQGISVTQTQMIRAFTAIANDGVMLEPKFISAIYDPNDQTARKSQKEIVGNPVSKDAASLTRTNMVLVGTDPVYGTMYNHSTGKPTVTVPGQNVALKSGTAQIADEKNGGYLVGLTNYIFSAVSMNPAENPDFILYV

>58

GTDGIITYEKDRLGNIVPGTEQVSQQTVDGKDVYTTISSPLQSFMETQMDAFQEKVKGKYMTATLVSAKTGEILATTQRPTFDADTKEGLTKDFVWRDILYQSNYEPGSTMKVMTLAAAIDNNTFPGGEVFNSSELKIADVTIRDWDVNDGLTTGRMMTFLQGFALSSNVGMSLLEQKMGDTTWLDYLNRFKFGVPTRFGLTDEYAGQLPADNIVNIAQSSFGQGISVTQTQMIRAFTAIANDGVMLEPKFISAIYDPNDQTARKSQKEIVGNPVSKDAASLTRTNMILVGTDPVYGTMYNHSTGKPTVTVPGQNVALKSGTAQIADEKNGGYLVGLTNYIFSAVSMNPAENPDFILYV

>59

GTDGIITYEKDRLGNIVPGTEQVSQRTMDGKDVYTTISSPLQSFMETQMDAFQEKVKGKYMTATLVSAKTGEILATTQRPTFDADTKEGITEDFVWRDILYQSNYEPGSTMKVMMLAAAIDNNTFPGGEVFNSSELKIADATIRDWDVNEGLTGGRMMTFSQGFAHSSNVGMTLLEQKMGDATWLDYLNRFKFGVPTRFGLTDEYAGQLPADNIVNIAQSSFGQGISVTQTQMIRAFTAIANDGVMLEPKFISAIYDPNDQTARKSQKEIVGNPVSKDAASLTRTNMVLIGTDPVYGTMYNHSTGKPTVTVPGQNVALKSGTAQIADEKNGGYLVGLTDYIFSAVSMSPAENPDFILYV

>60

GTDGIITYEKDRLGNIVPGTEQVSQQTVDGKDVYTTISSTLQSFMETQMNAFQEKVKGKYMTATLVSAKTGEILATTQRPTFDADTKEGITEDFVWRDILYQSNYEPGSPMKVMMLAAAIDNNTFPGGEVFNSSELKIADVTIRDWDVNEGLTGGRMMTFSQGFAHSSNVGMTLLEQKMGDATWLDYLNRFKFGVPTRFGLTDEYAGQLPADNIVNIAQSSFGQGISVTQTQMIRAFTAIANDGVMLEPKFISAIYDPNDQTARKSQKEIVGNPVSKDAASLTRTNMVLVGTDPVYGTMYNHSTGKPTVTVPGQNVALKSGTAQIADEKNGGYLVGVTDYIFSAVSMSPAENPDFILYV

>61

GTDGIITYEKDRLGNIVPGTEQVSQRTMDGKDVYTTISSPLQSFMETQMDAFQEKVKGKYMTATLVSAKTGEILATTQRPTFDADTKEGITEDFVWRDILYQSNYEPGSTMKVMMLAAAIDNNTFPGGEVFNSSELKIADATIRDWDVNEGLTGGRMMTFSQGFAHSSNVGMTLLEQKMGDATWLDYLNRFKFGVPTRFGLTDEYAGQLPADNIVNIAQSSFGQGISVTQTQMIRAFTAIANDGVMLEPKFISAIYDPNDQTARKSQKEIVGNPVSKDAASLTRTNMVLVGTDPVYGTMYNHSTGKPTVTVPGQNVALKSGAAQIADEKNGGYLVGVTDYIFSAVSMSPAENPDFILYV

>62

GTDGIITYEKDRVGNIVPGTELVSQQTVDGKDVYTTLSSPLQSFMETQMDAFLEKVKGKYMTATLVSAKTGEILATTQRPTFNADTKEGITEDFVWRDILYQSNYEPGSAFKVMMLASSIDNNTFPSGEYFNSSEFKIADATTRDWDVNAGLTTGGMMTFSQGFAHSSNVGTSLLEQKMGDATWLDYLKRFKFGVPTRFGLTDEYAGQLPADNIVSIAQSSFGQGISVTQTQMLRAFTAIANDGVMLEPKFISAIYDTNNQSVRKSQKEIVGNPVSKEAASTTRNHMILVGTDPLYGTMYNHYTGKPIITVPGQNVAVKSGTAQIADEKNGGYLVGSTNYIFSAVTMNPAENPDFILYV

>63

GTDGIITYEKDRLGNIVPGTEQVSQQTVDGKDVYTTISSPLQSFMETQMDAFLEKVKGKYMTATLVSAKTGEILATTQRPTFNADTKEGITEDFVWRDILYQSNYEPGSAMKVMTLASSIDNNTFPSGEYFNSSEFKIADATTRDWDVNDGLTTGGMMTFLQGFAHSSNVGMSLLEQKMGDATWLDYLKRFKFGVPTRFGLTDEYAGQLPADNIVSIAQSSFGQGISVTQTQMLRAFTAIANDGVMLEPKFISAIYDTNNQSVRKSQKEIVGNPVSKEAASTTRNHMILVGTDPLYGTMYNHYTGKPIITVPGQNVAVKSGTAQIADEKNGGYLVGSTNYIFSVVTMNPAENPDFILYV

>64

GTDGIITYEKDRLGNIVPGTEQVSQRTMDGKDVYTTISSPLQSFMETQMDAFQEKVKGKYMTATLVSAKTGEILATTQRPTFDADTKEGITEDFVWRDILYQSNYEPGSTMKVMTLAAAIDNNTFPGGEVFNSSELKIADATIRDWDVNEGLTGGRMMTFSQGFAHSSNVGMTLLEQKMGDATWLDYLNRFKFGVPTRFGLTDEYAGQLPADNIVNIAQSSFGQGISVTQTQMIRAFTAIANDGVMLEPKFISAIYDPNDQTARKSQKEIVGNPVSKDAASLTRTNMVLVGTDPVYGTMYNHSTGKPTVTVPGQNVALKSGTAQIADEKNGGYLVGLTDYIFSAVSMSPAENPDFILYV

>65

GTDGIITYEKDRLGNIVPGTEQVSQQTVDGKDVYTTISSPLQSFMETQMDAFQEKVKGKYMTATLVSAKTGEILATTQRPTFDADTKEGITEDFVWRDILYQSNYEPGSAMKVMTLASSIDNNTFPSGEYFNSSEFKIADATTRDWDVNDGLTTGGMMTFLQGFAHSSNVGMSLLEQKMGDATWLDYLKRFKFGVPTRFGLTDEYAGQLPADNIVSIAQSSFGQGISVTQTQMLRAFTAIANDGVMLEPKFISAIYDTNNQSVRKSQKEIVGNPVSKEAASTTRNHMILVGTDPLYGTMYNHYTGKPIITVPGQNVAVKSGTAQIADEKNGGYLVGSTNYIFSVVTMNPAENPDFILYV

>66

GTDGIITYEKDRLGNIVPGTEQVSQRTMDGKDVYTTISSPLQSFMETQMDAFQEKVKGKYMTATLVSAKTGEILATTQRPTFDADTKEGITEDFVWRDILYQSNYEPGSTMKVMMLAAAIDNNTFPGGEVFNSSELKIADATIRDWDVNEGLTGGRMMTFSQGFAHSSNVGMTLLEQKMGDATWLDYLNRFKFGVPTRFGLTDEYAGQLPADNIVNIAQSSFGQGISVTQTQMIRAFTAIANDGVMLEPKFISAIYDPNDQTARKSQKEIVGNPVSKDAASLTRTNMVLVGTDPVYGTMYNHSTGKPIVTVPGQNVALKSGTAQIADEKNGGYLVGVTDYIFSAVSMSPAENPDFILYV

>67

GTDGIITYEKDRLGNIVPGTEQVSQQTVDGKDVYTTISSPLQSFMETQMDAFQEKVKGKYMTATLVSAKTGEILATTQRPTFDADTKEGLTKDFVWRDILYQSNYEPGSTMKVMTLAAAIDNNTFPGGEVFNSSELKVADVTIRDWDVNEGLTGGRMMTFSQGFALSSNVGMTLLEQKMGDATWLDYLNRFKFGVPTRFGLTDEYAGQLPADNIVNIAQSSFGQGISVTQTQMIRAFTAIANDGVMLEPKFISAIYDPNDQTARKSQKEIVGNPVSKDAASLTRTNMVLVGTDPVYGTMYNHSTGKPTVTVPGQNVALKSGTAQIADEKNGGYLVGLTNYIFSAVSMNPAENPDFILYV

>68

GKDGIITYEKDRLGNIVPGTEQVSQQTVDGKDVYTTLSSPLQSFMETQMDAFLEKVKGKYMTATLVSAKTGEILATTQRPTFNADTKEGITEDFVWRDILYQSNYEPGSAMKVMTLASSIDNNTFPSGEYFNSSKFKIADATTRDWDVNDGLTTGGMMTFLQGFAHSSNVGMSLLEQKMGDATWLDYLKRFKFGVPTRFGLTDEYAGQLPADNIVSIAQSSFGQGISVTQTQMLRAFTAIANDGVMLEPKFISAIYDTNNQSVRKSQKEIVGNPVSKEAASTTRNHMILVGTDPLYGTMYNHYTGKPIITVPGQNVAVKSGTAQIADEKNGGYLVGSTNYIFSVVTMNPAENPDFILYV

>69

GTDGIITYEKDRLGNIVPGTEQVSQRTMDGKDVYTTISSPLQSFMETQMDAFQEKVKGKYMTATLVSAKTGEILATTQRPTFDADTKEGITEDFVWRDILYQSNYEPGSTMKVMMLAAAIDNNTFPGGEVFNSSELKIADATIRDWDVNEGLTGGRMMTFSQGFAHSSNVGMTLLEQKMGDATWLDYLNRFKFGVPTRFGLTDEYAGQLPADNIVNIAQSSFGQGISVTQTQMIRAFTAIANDGVMLEPKFISAIYDPNDQTARKSQKEVVGNPVSKDAASLTRTNMILVGTDPVYGTMYNHSTGKPTVNVPGQNVALKSGTAEIADEKNGGYLVGSTNNIFSVVAMNPAENPDFILYV

>70

GTDGIITYEKDRVGNIVPGTEQVSQQTVDGKDVYTTLSSPLQSFMETQMDAFLQKVKGKYMTATLVSAKTGEILATTQRPTFNADTKEGITEDFVWRDILYQSNYEPGSAFKVMMLASSIDNNTFPSGEYFNSSEFKIADATTRDWDVNAGLTTGGMMTFLQGFAHSSNVGTSLLEQKMGDATWLDYLKRFKFGVPTRFGLTDEYAGQLPADNIVSIAQSSFGQGISVTQTQMLRAFTAIANDGVMLEPKFISAIYDTNNQSVRKSQKEIVGNPVSKEAASTTRNHMILVGTDPLYGTMYNHYTGKPIITVPGQNVAVKSGTAQIADEKNGGYLVGSTNYIFSVVTMNPAENPDFILYV

>71

GTDGIITYEKDRLGNIVPGTEQVSQRTMDGKDVYTTISSPLQSFMETQMDAFQEKVKGKYMTATLVSAKTGEILATTQRPTFDADTKEGITEDFVWRDILYQSNYEPGSTMKVMMLAAAIDNNTFPGGEVFNSSELKIADATIRDWDVNEGLTGGRMMTFSQGFAHSSNVGMTLLEQKMGDATWLDYLNRFKFGVPTRFGLTDEYAGQLPADNIVNIAQSSFGQGISVTQTQMIRAFTAIANDGVMLEPKFISAIYDPNDQTARKSQKEIVGNPVSKDAASLTRTNMVLVGTDPVYGTMYNHSTGKPTVTVPGQNVALKSGTAQIADEKNGGYLVGVTDYIFSTVSMSPAENPDFILYV

>72

GTDGIITYEKDRLGNIVPGTEQVSQRTMDGKDVYTTISSPLQSFMETQMDAFQEKVKGKYMTATLVSAKTGEILATTQRPTFDADTKEGITEDFVWRDILYQSNYEPGSTMKVMMLAAAIDNNTFPGGEVFNSSELKIADATIRDWDVNEGLTGGRMMTFSQGFAHSSNVGMTLLEQKMGDATWLDYLNRFKFGVPTRFGLTDEYAGQLPADNIVNIAQSSFGQGISVTQTQMIRAFTAIANDGVMLEPKFITALYDPNDQTVRRSQKEIVGNPVSKDAASLTRTHMVLVGTDPRYGTMYNHGTGKATVNVPGQNVALKSGTAEIADEKNGGYLVGSTNNIFSVVAMNPAENPDFILYV

>73

GTDGIITYEKDRLGNIVPGTEQVSQRTMDGKDVYTTISSPLQSFMETQMDAFLEKVKGKYMTATLVSAKTGEILATTQRPTFNADTKEGITEDFVWRDILYQSNYEPGSAMKVMTLASSIDNNTFPSGEYFNSSEFKIADATTRDWDVNDGLTTGGMMTFLQGFAHSSNVGMSLLEQKMGDATWLDYLKRFKFGVPTRFGLTDEYAGQLPADNIVSIAQSSFGQGISVTQTQMLRAFTAIANDGVMLEPKFISAIYDTNNQSVRKSQKEIVGNPVSKEAASTTRNHMILVGTDPLYGTMYNHYTGKPIITVPGQNVAVKSGTAQIADEKNGGYLVGSTNYIFSVVTMNPAENPDFILYV

>74

GTDGIITYEKDRLGNIVPGTEQVSQRTMDGKDVYTTISSPLQSFMETQMDAFQEKVKRKYMTATLVSAKTGEILATTQRPTFDADTKEGITEDFVWRDILYQSNYEPGSTMKVMMLAAAIDNNTFPGGEVFNSSELKIADATIRDWDVNEGLTGGRMMTFSQGFAHSSNVGMTLLEQKMGDATWLDYLNRFKFGVPTRFGLTDEYAGQLPADNIVNIAQSSFGQGISVTQTQMIRAFTAIANDGVMLEPKFISAIYDPNDQTARKSQKEIVGNPVSKDAASLTRTNMVLVGTDPVYGTMYNHSTGKPTVTVPGQNVALKSGTAQIADEKNGGYLVGVTDYIFSAVSMSPAENPDFILYV

>75

GTDGIITYEKDRLGNIVPGTEQVSQRTMDGKDVYTTISSPLQSFMETQMDAFQEKVKGKYMTATLVSAKTGEILATTQRPTFDADTKEGITEDFVWRDILYQSNYEPGSTIKVMMLAAAIDNNTFPGGEVFNSSELKIADATIRDWDVNEGLTGGRMMTFSQGFAHSSNVGMTLLEQKMGDATWLDYLNRFKFGVPTRFGLTDEYAGQLPADNIVNIAQSSFGQGISVTQTQMIRAFTAIANDGVMLEPKFISAIYDPNDQTARKSQKEIVGNPVSKDAASLTRTNMVLVGTDPVYGTMYNHSTGKPTVTVPGQNVALKSGTAQIADEKNGGYLVGLTDYIFSAVSMSPAENPDFILYV

>76

GKDGIITYEKDRLGNIVPGTEQVSQQTVDGKDVYTTISSTLQSFMETQMDAFQEKVKGKYMTATLVSAKTGEILATTQRPTFDADTKEGLTKDFVWRDILYQSNYEPGSTMKVMTLASSIDNNTFPSGEYFNSSELKIADATIRDWDVNDGLTTGGMMTFLQGFAYSSNVGMSLLEQKMGDATWLDYLNRFKFGVPTRFGLTDEYSGQLPADNIVNIAMSAFGQGISVTQTQMLRAFTAIANDGVMLEPKFISAIYDPNDQTARKSQKEIVGNPVSKDAASLTRTNMVLVGTDPVYGTMYNHSTGKPTVTVPGQNVALKSGTAQIADEKNGGYLVGLTDYIFSAVSMSPAENPDFILYV

>77

GTDGIITYEKDRLGNIVPGTEQVFQQTVDGKDVYTTISSTLQSFMETQMNAFQEKVKGKYMTATLVSAKTGEILATTQRPTFDADTKEGLTKDFVWRDILYQSNYEPGSAMKVMTLAAAIDNNTFPGGEVFNSSELKVADVTIRDWDVNEGLTGGGMMTFSQGFAHSSNVGMTLLEQKMGDATWLDYLNRFKFGVPTRFGLTDEYAGQLPADNIVNIAQSSFGQGISVTQTQMIRAFTAIANDGVMLEPKFISAIYDPNDQTARKSQKEIVGNPVSKDAASLTRTNMVLVGTDPVYGTMYNHSTGKPTVTVPGQNVALKSGTAQIADEKNGGYLVGLTNYIFSAVSMSPAENPDFILYV

>78

GTDGIITYEKDRLGNIIPGTEQVSQQTVDGKDVYTTISSPLQSFMETQMDAFQEKVKGKYMTATLVSAKTGEILATTQRPTFDADTKEGITEDFVWRDILYQSNYEPGSAMKVMTLAASIDNNTFPGGEYFNSSELKIADVTIRDWDVNEGLTGGGMMTFSQGFAHSSNVGMTLLEQKMGDATWLDYLNRFKFGVPTRFGLTDEYAGQLPADNIVNIAQSSFGQGISVTQTQMIRAFTAIANDGVMLEPKFISAIYDPNDQTARKSQKEIVGNPVSKDAASLTRTNMVLVGTDPVYGTMYNHSTGKPTVTVPGQNVALKSGTAQIADEKNGGYLVGLTNYIFSAVSMSPAENPDFILYV

>79

GKDGIITYEKDRLGNIVPGTEQVSQQTVDGKDVYTTISSTLQSFMETQMNVFQEKVKGKYMTATLVSAKTGEILATTQRPTFDADTKEGLTKDFVWRDILYQSNYEPGSTMKVMTLAAAIDNNTFPGGEVFNSSELKVADTTIRDWDVNEGLTGGGMMTFSQGFAHSSNVGMTLLEQKMGDATWLDYLSRFKFGVPTRFGLTDEYAGQLPADNIVNIAQSSFGQGISVTQTQMIRAFTAIANDGVMLEPKFISALYDPNDQSVRKSQKEIVGNPVSKDAASLTRTNMVLVGTDPVYGTMHNHSTGKPTITVPGQNVALKSGTAQIADEKNGGYLVGTTNHIFSAVSMNPAENPDFILYV

>80

GTDGIITYEKDRLGNIVPGTELVSQQTVDGKDVYTTLSSPLQSFMETQMDAFLEKVKGKYMTATLVSAKTGEILATTQRPTFNADTKEGITEDFVWRDILYQSNYEPGSTMKVMTLASSIDNNTFPSGEYFNSSELKIADVTIRDWDVNDGLTTGRMMTFLQGFALSSNVGMSLLEQKMGDTTWLDYLNRFKFGVPTRFGLTDEYAGQLPADNIVNIAQSSFGQGISVTQTQMIRAFTAIANDGVMLEPKFISAIYDPNDQTARKSQKEIVGNPVSKDAASLTRTNMILVGTDPVYGTMYNHSTGKPTVTVPGQNVALKSGTAQIADEKNGGYLVGLTNYIFSAVSMNPAENPDFILYV

>81

GTDGIITYEKDRLGNIVPGTEQVSQRTMDGKDVYTTISSPLQSFMETQMDAFQEKVKGKYMTATLVSAKTGEILATTQRPTFDADTKEGITEDFVWRDILYQSNYEPGSTMKVMTLAAAIDNNTFPGGEVFNSSELKIADATIRDWDVNEGLTGGRMMTFSQGFAHSSNVGMTLLEQKMGDATWLDYLNRFKFGVPTRFGLTDEYAGQLPADNIVNIAQSSFGQGISVTQTQMIRAFTAIANDGVMLEPKFISAIYDPNDQTARKSQKEIVGNPVSKDAASLTRTNMVLVGTDPVYGTMYNHSTGKPTVTVPGQNVAIKSGTAQIADEKNGGYLVGLTDYIFSAVSMSPAENPDFILYV

>82

GKDGIITYEKDRLGNIVPGTEQVSQQTVDGKDVYTTISSTLQSFMETQMDAFQEKVKGKYMTATLVSAKTGEILATTQRPTFNADTKEGITEDFVWRDILYQSNYEPGSAFKVMTLASSIDNNTFPSGEYFNSSEFKIADATTRDWDVNAGLTTGGMMTFSQGFAHSSNVGTSLLEQKMGDATWLDYLKRFKFGVPTRFGLTDEYAGQLPADNIVSIAQSSFGQGISVTQTQMLRAFTAIANDGVMLEPKFISAIYDTNNQSVRKSQKEIVGNPVSKEAASTTRNHMILVGTDPLYGTMYNHYTGKPIITVPGQNVAVKSGTAQIADEKNGGYLVGSTNYIFSVVTMNPAENPDFILYV

>83

GTDGIITYEKDRLGNIVPGTEQVSQRTMDGKDVYTTISSPLQSFMEPQMDAFQEKVKGKYMTATLVSAKTGEILATTQRPTFDADTKEGITEDFVWRDILYQSNYEPGSTMKVMMLAAAIDNNTFPGGEVFNSSELKIADATIRDWDVNEGLTGGRMMTFSQGFAHSSNVGMTLLEQKMGDATWLDYLNRFKFGVPTRFGLTDEYAGQLPADNIVNIAQSSFGQGISVTQTQMIRAFTAIANDGVMLEPKFISAIYDPNDQTARKSQKEIVGNPVSKDAASLTRTNMVLVGTDPVYGTMYNHSTGKPTVTVPGQNVALKSGTAQIADEKNGGYLVGLTDYIFSAVSMSPAENPDFILYV

>84

GTDGIITYEKDRLGNIVPGTEQVSQRTMDGKDVYTTISSPLQSFMETQMDAFQEKVKGKYMTATLVSAKTGEILATTQRPTFDADTKEGITEDFVWRDILYQSNYEPGSTMKVMMLAAAIDNNTFPGGEVFNSSELKIADATIRDWDVNEGLTGGRMMTFSQGFAHSSNVGMTLLEQKMGDATWLDYLNRFKFGVPTRFGLTDEYAGQLPADNIVNIAQSSFGQGISVTQTQMIRAFTAIANDGVMLEPKFISAIYDPNDQTARKSQKEIVGNPVSKDAASLTRTNMVLVGTDPVYGSMYNHSTGKPTVTVPGQNVALKSGTAQIADEKNGGYLVGLTDYIFSAVSMSPAENPDFILYV

>85

GTDGIITYEKDRLGNIVPGTEQVSQQTVDGKDVYTTLSSPLQSFMETQMDAFLEKVKGKYMTATLVSAKTGEILATTQRPTFNADTKEGITEDFVWRDILYQSNYEPGSAMKVMTLASSIDNNTFPSGEYFNSSEFKIADATTRDWDVNEGLTTGGMMTFLQGFAHSSNVGMSLLEQKMGDATWLDYLKRFKFGVPTRFGLTDEYAGQLPADNIVSIAQSSFGQGISVTQTQMLRAFTAIANDGVMLEPKFISAIYDTNNQSVRKSQKEIVGNPVSKEAASTTRNHMILVGTDPLYGTMYNHYTGKPIITVPGQNVAVKSGTAQIADEKNGGYLVGSTNYIFSVVTMNPAENPDFILYV

>86

GTDGIITYEKDRLGNIVPGTEQVSQRTMDGKDVYTTISSPLQSFMETQMDAFQEKVKGKYMTATLVSAKTGEILATTQRPTFDADTKEGLTKDFVWRDILYQSNYEPGSTMKVMTLAAAIDNNTFPGGEVFNSSELKVADVTIRDWDVNEGLTGGRMMTFSQGFALSSNVGMTLLEQKMGDATWLDYLNRFKFGVPTRFGLTDEYAGQLPADNIVNIAQSSFGQGISVTQTQMIRAFTAIANDGVMLEPKFISAIYDPNDQTARKSQKEIVGNPVSKDAASLTRTNMILVGTDPVYGTMYNHSTGKPTVTVPGQNVALKSGTAQIADEKNGGYLVGLTNYIFSAVSMNPAENPDFILYV

>87

GKDGIITYEKDRLGNIVPGTEQVSQQTVDGKDVYTTISSTLQSFMETQMNAFQEKVKGKYMTATLVSAKTGEILATTQRPTFDADTKEGLTKDFVWRDILYQSNYEPGSPMKVMTLASSIDNNTFPGGEYFNSSELKVADVTTRDWDVNEGLTSGGMMTFSQSFAHSSNVGMTLLEQKMGDATWLDYLNRFKFGVPTRFGLTDEYAGQLPADNIVSIAQSSFGQGISVTQTQMIRAFTAIANDGVMLEPKFISAIYDPNDQTARKSQKEVVGNPVSKDAASLTRTHMVLVGTDPVYGTMYNHKTGKPTVTVPGQNVALKSGTAEIADEKNGGYLVGTTNYIFSAVSMNPAENPDFILYV

>88

GTDGIITYEKDRLGNIVPGTEQVSQRTMDGKDVYTTISSPLQSFMETQMDAFQEKVKGKYMTATLVSAKTGEILATTQRPTFDADTKEGITEDFVWRDILYQSNYEPGSTMKVMMLAAAIDNNTFPGGEVFNSSELKIADATIRDWDVNEGLTGGRTMTFSQGFAHSSNVGMTLLEQKMGDTTWLDYLNRFKFGVPTRFGLTDEYAGQLPADNIVNIAQSSFGQGISVTQTQMIRAFTAIANDGVMLEPKFISAIYDPNDQTARKSQKEIVGNPVSKDAASLTRTNMVLVGTDPVYGTMYNHSTGKPTVTVPGQNVALKSGTAQIADEKNGGYLVGLTDYIFSAVSMSPAENPDFILYV

>89

GKDGIITYEKDRLGNIVPGTEQVSQQTVDGKDVYTTISSTLQSFMEIQMNAFQEKVKGKYMTATLVSAKTGEILATTQRPTFDADTKEGLTKDFVWRDILYQSNYEPGSTMKVMTLAAAIDNNTFPGGEVFNSSELKIADVTIRDWDVNEGLTGGRMMTFSQGFALSSNVGMTLLEQKMGDATWLDYLNRFKFGVPTRFGLTDEYAGQLPADNIVNIAQSSFGQGISVTQTQMLRAFTAIANDGVMLEPKFISAIYDTNNQSVRKSQKEIVGKPVSEDAASLTRTNMILVGTDPLYGTMYNHQTGKPIITVPGQNVAVKSGTAQIADEKNGGYLVGSTNYIFSVVTMNPAENPDFILYV

>90

GKDGIITYEKDRLGNIVPGTEQVSQQTVDGKDVYTTISSTLQSFMETQMNAFQEKVKGKYMTATLVSAKTGEILATTQRPTFDADTKEGLTKDFVWRDILYQSNYEPGSPMKVMMLAAAIDNNTFPGGEVFNSSELKVADVTTRDWDVNEGLTSGGMMTFSQSFAHSSNVGMTLLEQKMGDATWLDYLNRFKFGVPTRFGLTDEYAGQLPADNIVSIAQSSFGQGISVTQTQMIRAFTAIANDGVMLEPKFISAIYDPNDQTARKSQKEVVGNPVSKDAASLTRTHMVLVGTDPVYGTMYNHKTGKPTVTVPGQNVALKSGTAEIADEKNGGYLVGSTNYIFSVVAMNPAENPDFILYV

>91

GTDGIITYEKDRLGNIVPGTEQVSQQTVDGKDVYTTISSTLQSFMETQMNAFQEKVKGKYMTATLVSAKTGEILATTQRPTFDADTKEGLTKDFVWRDILYQSNYEPGSSMKVMTLAAAIDNNTFPGGEVFNSSELKVADTTTRDWDVNEGLTGGGMMTFSQGFAHSSNVGMTLLEQKMGDATWLDYLNRFKFGVPTRFGLTDEYAGQLPADNIVNIAQSSFGQGISVTQTQMIRAFTAIANDGVMLEPKFISAIYDPNDQTARKSQKEVVGNPVSKDAASLTRTHMVLVGTDPVYGTMYNHKTGKPTVTVPGQNVALKSGTAQIADEKNGGYLVGLTNYIFSAVSMNPAENPDFILYV

>92

GTDGIITYEKDRLGNIVPGTEQVSQQTVDGKDVYTTISSTLQSFMETQMNAYQEKVKGKYMTATLVSAKTGEILATTQRPTFDADTKEGLTKDFVWRDILYQSNYEPGSPMKVMTLAAAIDNNTFPGGEVFNSSELKVADVTTRDWDVNEGLTSGGMMTFSQGFAHSSNVGMSLLEQKMGDATWLDYLNRFKFGVPTRFGLTDEYAGQLPADNIVSIAQSSFGQGISVTQTQMIRAFTAIANDGVMLEPKFISAIYDPNDQTARKSQKEVVGNPVSKDAASLTRTHMVLVGTDPVYGTMYNHKTGKPTVTVPGQNVALKSGTAQIADEKNGGYLVGVTDYIFSAVSMNPAENPDFILYV

>93

GTDGIITYEKDRLGNIVPGTEQVSQQTVDGKDVYTTISSPLQSFMETQMDAFQEKVKGKYMTATLVSAKTGEILATTQRPTFDADTKEGITEDFVWRDILYQSNYEPGSTMKVMMLAAAIDNNTFPGGEVFNSSELKIADATIRDWDVNEGLTGGRMMTFSQGFAHSSNVGMTLLEQKMGDATWLDYLNRFKFGVPTRFGLTDEYAGQLPADNIVNIAMSAFGQGISVTQTQMLRAFTAIANDGVMLEPKFISALYDPNDQTVRKSQKEIVGNPVSKDAASQTRTHMVLVGTDPRYGTMYNHSTGKATVNVPGQNVALKSGTAEIADEKNGGYLVGSTNNIFSVVAMNPAENPDFILYV

>94

GKDGIITYEKDRLGNIVPGTEQVSQQTVDGKDVYTTISSTLQSFMETQMNAFQEKVKGKYMTATLVSAKTGEILATTQRPTFDADTKEGLTKDFVWRDILYQSNYEPGSTMKVMTLAAAIDNNTFPGGEVFNSSELKVADATIRDWDVNEGLTGGRMMTFSQGFALSSNVGMTLLEQKMGDATWLDYLNRFKFGVPTRFGLTDEYAGQLPADNIVNIAQSSFGQGISVTQTQMIRAFTAIANDGVMLEPKFITALYDPNDQTVRKSQKEIVGNPVSKDAASQTRTHMVLVGTDPTYGTMHNHSTGKPTVTVPGQNVALKSGTAEIADEKNGGYLVGTTNYIFSAVSMNPAENPDFILYV

>95

GTDGIITYEKDRLGNIVPGTEQVSQRTMDGKDVYTTISSPLQSFMETQMDAFQEKVKGKYMTATLVSAKTGEILATTQRPTFDADTKEGITEDFVWRDILYQSNYEPGSTMKVMMLAAAIDNNTFPGGEVFNSSELKIADATIRDWDVNEGLTGGRMMTFSQGFAHSSNVGMTLLEQKMGDATWLDYLNRFKFGVPTRFGLTDEYAGQLPADNIVNIAQSSFGQGISVTQTQMIRAFTAIANDGVMLEPKFISAIYDPNDQTARKSQKEIVGNPVSKDAASLTRTNMVLVGTDPVYGTMYNHSTGKPTVTVPGQNVALKSGTAQIADEKNGGYLVGVTDYIFSAVSMSPTENPDFILYV

>96

GTDGIITYEKDRVGNIVPGTELVSQQTVDGKDVYTTLSSPLQSFMETQMDAFLEKVKGKYMTATLVSAKTGEILATTQRPTFNADTKEGITEDFVWRDILYQSNYEPGSAFKVMTLASSIDNNTFPSGEYFNSSEFKIADATTRDWDVNAGLTTGGMMTFSQGFAHSSNVGTSLLEQKMGDATWLDYLKRFKFGVPTRFGLTDEYAGQLPADNIVSIAQSSFGQGISVTQTQMLRAFTAIANDGVMLEPKFISAIYDTNNQSVRKSQKEIVGNPVSKEAASTTRNHMILVGTDPLYGTMYNHYTGKPIITVPGQNVAVKSGTAQIADEKNGGYLVGSTNYIFSVVTMNPAENPDFILYV

>97

GTDGIITYEKDRLGNIVPGTEQVSQRTMDGKDVYTTISSPLQSFMETQMDAFQEKVKGKYMTATLVSAKTGEILATTQRPTFDADTKEGLTKDFVWRDILYQSNYEPGSAMKVMTLASSIDNNTFPSGEYFNSSEFKIADATTRDWDVNEGLTTGGMMTFLQGFAHSSNVGMSLLEQKMGDATWLDYLNRFKFGVPTRFGLTDEYAGQLPADNIVSIAQSSFGQGISVTQTQMLRAFTAIANDGVMLEPKFISAIYDTNNQSVRKSQKEIVGNPVSKEAASTTRNHMILVGTDPLYGTMYNHYTGKPIITVPGQNVAVKSGTAQIADEKNGGYLVGSTNYIFSAVTMNPAENPDFILYV

>98

GTDGIITYEKDRLGNIVPGTEQVSQRTMDGKDVYTTISSPLQSFMETQMDAFQEKVKGKYVTATLVSAKTGEILATTQRPTFDADTKEGITEDFVWRDILYQSNYEPGSTMKVMMLAAAIDNNTFPGGEVFNSSELKIADATIRDWDVNEGLTGGRMMTFSQGFAHSSNVGMTLLEQKMGDATWLDYLNRFKFGVPTRFGLTDEYAGQLPADNIVNIAQSSFGQGISVTQTQMIRAFTAIANDGVMLEPKFISAIYDPNDQTARKSQKEIVGNPVSKDAASLTRTNMVLVGTDPVYGTMYNHSTGKPTVTVPGQNVALKSGTAQIADEKNGGYLVGVTDYIFSAVSMSPTENPDFILYV

>99

GTDGIITYEKDRLGNIVPGTEQVSQRTMDGKDVYTTISSPLQSFMETQMDAFQEKVKGKYMTATLVSAKTGEILATTQRPTFDADTKEGITEDFVWRDILYQSNYEPGSTMKVMMLAAAIDNNTFPGGEYFNSSELKIADATIRDWDVNEGLTSGGTMTFSQGFAHSSNIGMTLLEQKMGDATWLDYLNRFKFGVPTRFGLTDEYTGQLPADNIVNIAMSAFGQGISVTQTQMLRAFTAIANDGVMLEPKFISALYDPNDQSVRKSQREIVGNPVSKEAASVTRDHMVMVGTDPTYGTMYNHSTGKATVNVPGQNVALKSGTAEIADEKNGGYLVGSTNNIFSVVAMNPAENPDFILYV

>100

GTDGIITYEKDRVGNIVPGTELVSQQTVDGKDVYTTLSSPLQSFMETQMDAFLEKVKGKYMTATLVSAKTGEILATTQRPTFNADTKEGITEDFVWRDILYQSNYEPGSGMKVMTLASSIDNNTFPSGEYFNSSEFKIADATTRDWDVNEGLTTGGMMTFLQGFAHSSNVGMSLLEQKMGDATWLDYLKRFKFGVPTRFGLTDEYAGQLPADNIVSIAQSSFGQGISVTQTQMLRAFTAIANDGVMLEPKFISAIYDTNNQSVRKSQKEIVGNPVSKEAASTTRNHMILVGTDPLYGTMYNHYTGKPIITVPGQNVAVKSGTAQIADEKNGGYLVGSTNYIFSVVTMNPAENPDFILYV

>101

GTDGIITYEKDRLGNIVPGTEQVSQRTMDGKDVYTTISSPLQSFMETQMDAFQEKVKGKYMTATLVSAKTGEILATTQRPTFDADTKEGITEDFVWRDILYQSNYEPGSTMKVMMLAAAIDNNTFPGGEVFNSSELKIADATIRDWDVNEGLTGGRMMTFSQGFAHSSNVGMTLLEQKMGDATWLDYLNRFKFGVPTRFGLTDEYAGQLPADNIVNIAQSSFGQGISVTQTQMIRAFTAIANDGVMLEPKFISAIYDPNDQTARKSQKEIVGNPVSKDATSLTRTNMVLVGTDPVYGTMYNHSTGKPTVTVPGQNVALKSGTAQIADEKNGGYLVGLTDYIFSAVSMSPTENPDFILYV

>102

GTDGIITYEKDRLGNIVPGTEQVSQQTVDGKDVYTTISSPLQSFMETQMDAFQEKVKGKYMTATLVSAKTGEILATTQRPTFDADTKEGITEDFVWRDILYQSNYEPGSTMKVMMLAAAIDNNTFPGGEVFNSSELKIADATIRDWDVNEGLTGGRMMTFLQGFAHSSNVGMSLLEQKMGDATWLDYLNRFKFGVPTRFGLTDEYAGQLPADNIVNIAMSAFGQGISVTQTQMLRAFTAIANDGVMLEPKFITALYDPNNQSVRKSQKEIVGNPVSKDAASQTRTHMVLVGTDPRYGTMYNHSTGKATVNVPGQNVALKSGTAEIADEKNGGYLVGSTNNIFSVVAMNPAENPDFILYV

>103

GKDGIITYEKDRLGNIVPGTEQVSQQTVDGKDVYTTISSTLQSFMETQMDAFQEKVKGKYMTATLVSAKTGEILATTQRPTFDADTKEGLTKDFVWRDILYQSNYEPGSTMKVMTLASSIDNNTFPSGEYFNSSELKIADATIRDWDVNDGLTTGGMMTFLQGFAYSSNVGMSLLEQKMGDATWLDYLNRFKFGVPTRFGLTDEYSGQLPADNIVNIAMSAFGQGISVTQTQMLRAFTAIANDGVMLEPKFISALYDPNDQTVRKSQKEIVGNPVSKDAASQTRTHMVLVGTDPRYGTMYNHSTGKPTVTVPGQNVALKSGTAQIADEKNGGYLVGVTDYIFSAVSMSPAENPDFILYV

>104

GTDGIITYEKDRLGNIVPGTEQVSQRTMDGKDVYTTISSPLQSFMETQMDAFQEKVKGKYMTATLVSAKTGEILATTQRPTFDADTKEGITEDFVWRDILYQSNYEPGSTMKVMMLAAAIDNNTFPGGEVFNSSELKIADATIRDWDVNEGLTGGRMMTFSQGFAHSSNVGMTLLEQKMGDATWLDYLNRFKFGVPTRFGLADEYTGQLPADNIVNIAMSSFGQGISVTQTQMLRAFTAIANDGVMLEPKFISALYDPNDQSVRKSQKEIVGNPVSKAAASSTRDHMVMVGTDPVYGTMYNHSTGKPNVNVPGQNVALKSGTAEIADEKNGGYLTGETNNIFSVVSMHPAENPDFILYV

>105

GTDGIITYEKDRLGNIVPGTEQVSQQTVDGKDVYTTISSTLQSFMETQMNAFQEKVKGKYMTATLVSAKTGEILATTQRPTFDADTKEGLTKDFVWRDILYQSNYEPGSTMKVMTLASSIDNNTFPSGEYFNSSELKIADATIRDWDVNDGLTTGGMMTFLQGFAHSSNVGMSLLEQKMGDATWLDYLNRFKFGVPTRFGLTDEYAGQLPADNIVNIAMSAFGQGISVTQTQMLRAFTAIANDGVMLEPKFISAIYDTNNQSVRKSQKEIVGNPVSKEAASTTRNHMILVGTDPLYGTMYNHYTGKPIITVPGQNVAVKSGTAQIADEKNGGYLVGSTNYIFSVVTMNPAENPDFILYV

>106

GTDGIITYEKDRLGNIVPGTEQVSQRTMDGKDVYTTISSPLQSFMETQMDAFQEKVKGKYMTATLVSAKTGEILATTQRPTFDADTKEGITEDFVWRDILYQSNYEPGSTMKVMMLAAAIDNNTFPGGEVFNSSELKIADATIRDWDVNEGLTGGRMMTFSQGFAHSSNVGMTLLEQKMGDATWLDYLNRFKFGVPTRFGLTDEYAGQLPADNIVNIAQSSFGQGISVTQTQMIRAFTAIANDGVMLEPKFISAIYDPNDQTARKSQKEIVGNPVSKDAASLTRTNMVLVGTDPVYGTMYNHSTGKPTVTVPGQNVALKSGTAQIADEKNGGYLVGLTDYIFSAVSMSPAESPDFILYV

>107

GTDGIITYEKDRLGNIVPGTEQVSQRTMDGKDVYTTISSPLQSFMETQMDAFQEKVKGKYMTATLVSAKTGEILATTQRPTFDADTKEGITEDFVWRDILYQSNYEPGSTMKVMTLASSIDNNTFPSGEYFNSSELKIADATIRDWDVNDGLTTGGMMTFLQGFAHSSNVGMSLLEQKMGDATWLDYLNRFKFGVPTRFGLTDEYSGQLPADNIVNIAMSAFGQGISVTQTQMLRAFTAIANDGVMLEPKFISAIYDPNDQTARKSQKEVVGNPVSKDAASLTRTNMILVGTDPVYGTMYNHSTGKPTVTVPGQNVALKSGTAQIADEKNGGYLVGLTNYIFSAVSMNPAENPDFILYV

>108

GKDGIITYEKDRLGNIVPGTEQVSQQIVDGKDVYTTISSTLQSFMETQMNAFQEKVKGKYMTATLVSAKTGEILATTQRPTFDADTKEGLTKDFVWRDILYQSNYEPGSTMKVMTLAAAIDNNTFPGGEVFNSSELKVADVTIRDWDVNEGLTGGGMMTFSQGFALSSNVGMTLLEQKMGDATWLDYLSRFKFGVPTRFGLTDEYAGQLPADNIVNIAQSSFGQGISVTQTQMIRAFTAIANDGVMLEPKFISAIYDPNDQTARKSQKEVVGNPVSKAAASSTREHMVMVGTDPVYGTMYNHSTGKPNVNVPGQNVALKSGTAEIADEKNGGYLTGETNNIFSVVSMHPAENPDFILYV

>109

GKDGIITYEKDRLGNIVPGTEQVSQQTVDGKDVYTTLSSPLQSFMETQMDAFQEKLKGKYMTATLVSAKTGEILATTQRPTFNADTKEGITEDFVWRDILYQSNYEPGSAMKVMTLASSIDNNTFPSGEYFNSSEFKIADATTRDWDVNAGLTTGGMMTFLQGFAHSSNVGMSLLEQKMGDATWLDYLKRFKFGVPTRFGLTDEYAGQLPADNIVSIAQSSFGQGISVTQTQMLRAFTAIANDGVMLEPKFISAIYDTNNQSVRKSQKEIVGNPVSKEAASTTRNHMILVGTDPLYGTMYNHYTGKPIITVPGQNVAVKSGTAQIADEKNGGYLVGSTNYIFSAVTMNPAENPDFILYV

>110

GTDGIITYEKDRLGNIVPGTEQVSQRTMDGKDVYTTISSPLQSFMETQMDAFQEKVKGKYMTATLVSAKTGEILATTQRPTFDADTKEGITEDFVWRDILYQSNYEPGSTMKVMMLAAAIDNNTFPGGEVFNSSELKIADATIRDWDVNEGLTGGRMMTFSQGFAHSSNVGMTLLEQKMGDATWLDYLNRFKFGVPTRFGLTDEYAGQLPADNIVNIAQSSFGQGISVTQTQMIRAFTAIANDGVMLEPKFISAIYDPNDQTARKSQKEIVGNPVSKDAASLTRTNMVLVGTDPVYGTMYNHSTGKPTVTVPGQNVALKSGTAEIADEKNGGYLVGSTNNIFSVVAMNPAEDPDFILYV

>111

GTDGIITYEKDRLGNIVPGTEQASQHTVDGKDVYTTLSSPLQSFMETQMDAFQEKVKGKYMTATLVSAKTGEILATTQRPTFNADTKDGITKDFVWRDILYQSNYEPGSAMKVMTLASAIDNNTFPGGEYFNSSELKIADATIRDWDVNDGLTTGGMMTFSQGFAHSSNVGMSLLEQKMGDATWLDYLNRFKFGVPTRFGLTDEYTGQLPADNIVNIAMSAFGQGISVTQTQMLRAFTAIANDGVMLEPKFISALYDPNDQSVRKSQKEIVGNPVSKAAASSTREHMVMVGTDPVYGTMYNHSTGKPNVNVPGQNVALKSGTAQIADEKNGGYLTGETNYIFSVVSMHPAENPDFILYV

>112

GKDGIITYEKDRLGNIVPGTEQVSQQTVDGKDVYTTLSSPLQSFMETQMDAFLEKVKGKYMTATLVSAKTGEILATTQRPTFNADTKEGITEDFVWRDILYQSNYEPGSAMKVMTLASSIDNNTFPSGEYFNSSEFKIADATTRDWDVNEGLTTGGMMTFLQGFAHSSNVGMSLLEQKMGDATWLDYLKRFKFGVPTRFGLTDEYAGQLPADNIVSIAQSSFGQGISVTQTQMLRAFTAIANDGVMLEPKFISAIYDTNNQSVRKSQKEIVGNPVSKEAASTTRNHMILVGTDPLYGTMYNHYTGKPIITVPGQNVAVKSGTAQIADEKNGGYLVGSTNYIFSVVTMNPAENPDFILYV

>113

GTDGIITYEKDRVGNIVPGTELVSQQTVDGKDVYTTLSSPLQSFMETQMDAFLQKVKGKYMTATLVSAKTGEILATTQRPTFNADTKEGITEDFVWRDILYQSNYEPGSAFKVMMLASSIDNNTFPSGEYFNSSEFKIADATTRDWDVNAGLTTGGMMTFLQGFAHSSNVGTSLLEQKMGDATWLDYLKRFKFGVPTRFGLTDEYAGQLPADNIVSIAQSSFGQGISVTQTQMLRAFTAIANDGVMLEPKFISAIYDTNNQSVRKSQKEIVGNPVSKEAASTTRNHMILVGTDPLYGTMYNHYTGKPIITVPGQNVAVKSGTAQIADEKNGGYLVGSTNYIFSVVTMNPAENPDFILYV

>114

GTDGIITYEKDRLGNIVPGTEQVSQRTMDGKDVYTTISSPLQSFMETQMDAFQEKVKGKYMTATLVSAKTGEILATTQRPTFDADTKEGITEDFVWRDILYQSNYEPGSAMKVMTLASSIDNNTFPSGEYFNSSEFKIADATTRDWDVNEGLTTGGMMTFLQGFAHSSNVGMSLLEQKMGDATWLDYLKRFKFGVPTRFGLTDEYAGQLPADNIVSIAQSSFGQGISVTQTQMLRAFTAIANDGVMLEPKFISAIYDTNNQSVRKSQKEIVGNPVSKEAASTTRNHMILVGTDPLYGTMYNHYTGKPIITVPGQNVAVKSGTAQIADEKNGGYLVGSTNYIFSAVTMNPAENPDFILYV

>115

GKDGIITYEKDRVGNIVPGTELVSQQTVDGKDVYTTLSSPLQSFMETQMDAFLEKVKGKYMTATLVSAKTGEILATTQRPTFNADTKEGITEDFVWRDILYQSNYEPGSAFKVMMLASSIDNNTFPSGEYFNSSEFKIADATTRDWDVNAGLTTGGMMTFLQGFAHSSNVGTSLLEQKMGDATWLDYLKRFKFGVPTRFGLTDEYAGQLPADNIVSIAQSSFGQGISVTQTQMLRAFTAIANDGVMLEPKFISAIYDTNNQSVRKSQKEIVGNPVSKEAASTTRNHMILVGTDPLYGTMYNHYTGKPIITVPGQNVAVKSGTAQIADEKNGGYLVGSTNYIFSVVTMNPAENPDFILYV

>116

GTDGIITYEKDRLGNIVPGTEQVSQQTVDGKDVYTTISSPLQSFMETQMDAFQEKVKGKYMTATLVSAKTGEILATTQRPTFDADTKEGITEDFVWRDILYQSNYEPGSPMKVMMLAAAIDNNTFPGGEVFNSSELKIADVTIRDWDVNEGLTGGRMMTFSQGFAHSSNVGMTLLEQKMGDATWLDYLNRFKFGVPTRFGLTDEYAGQLPADNIVNIAQSSFGQGISVTQTQMIRAFTAIANDGVMLEPKFISAIYDPNDQTARKSQKEIVGNPVSKDAASLTRTNMVLVGTDPVYGTMYNHSTGKPTVTVPGQNVALKSGTAQIADEKNGGYLVGLTNYIFSAVSMNPAENPDFILYV

>117

GTDGIITYEKDRLGNIVPGTEQVSQQTVDGKDVYTTISSPLQSFMETQMDAFQEKVKGKYMTATLVSAKTGEILATTQRPTFDADTKEGITEDFVWRDILYQSNYEPGSSMKVMTLAAAIDNNTFPGGEVFNSSELKVADTTTRDWDVNEGLTGGGMMTFSQGFAHSSNVGMTLLEQKMGDATWLDYLNRFKFGVPTRFGLTDEYAGQLPADNIVNIAQSSFGQGISVTQTQMIRAFTAIANDGVMLEPKFISAIYDPNDQTARKSQKEVVGNPVSKDAASLTRTHMVLVGTDPVYGTMYNHKTGKPTVTVPGQNVALKSGTAQIADEKNGGYLVGLTNYIFSAVSMNPAENPDFILYV

>118

GTDGIITYEKDRVGNIVPGTELVSQQTVDGKDVYTTLSSPLQSFMETQMDAFLEKVKGKYMTATLVSAKTGEILATTQRPTFNADTKEGITEDFVWRNILYQSNYEPGSAMKVMTLASSIDNNTFPSGEYFNSSEFKIADATTRDWDVNEGLTTGGMMTFLQGFAHSSNVGMSLLEQKMGDATWLDYLKRFKFGVPTRFGLTDEYAGQLPADNIVSIAQSSFGQGISVTQTQMLRAFTAIANDGVMLEPKFISAIYDTNNQSVRKSQKEIVGKPVSEDTASLTRTNMILVGTDPLYGTMYNHYTGKPIITVPGQNVAVKSGTAQIADEKNGGYLVGSTNYIFSAVTMNPAENPDFILYV
