# Supplementary material for: Strain features and distributions in pneumococci from children with invasive disease before and after 13-valent conjugate vaccine implementation in the USA
Source: Clin Microbiol Infect. 2016 Jan;22(1):60.e9–60.e29. doi: 10.1016/j.cmi.2015.08.027 (PMC4721534; doi:10.1016/j.cmi.2015.08.027)
Supplement: Table S6 — Identifiers for accession of fastq files provided by Sanger for this work at the European Nucleotide Archive (http://www.ebi.ac.uk/ena) and for fastq files provided by the CDC Biotechnology Core Facility at http://www.ncbi.nlm.nih.gov/genbank/genomessubmit [file mmc6.docx]

**sTable 6.**

STable 6. Accession of fastq files at <http://www.ebi.ac.uk/ena> (Err designations, Bioproject [PRJEB3084](http://www.ebi.ac.uk/ena/data/view/PRJEB3084)) and <http://www.ncbi.nlm.nih.gov/genIbank/> (SAMN designations, Bioproject PRJNA284954)

CDC Lab ID Accession isolation year

2009206314 ERR742343 2008

2009201154 ERR742344 2008

2009201416 ERR742345 2008

2009202614 ERR742346 2008

2008232452 ERR742399 2008

2008232504 ERR742400 2008

2008235379 ERR742401 2008

2008235380 ERR742402 2008

2009203640 ERR742403 2008

2008228002 ERR742404 2008

2008229142 ERR742405 2008

2008237702 ERR742406 2008

2008238089 ERR742407 2008

2009201003 ERR742408 2008

2008230642 ERR742410 2008

2008232792 ERR742411 2008

2008232831 ERR742412 2008

2008232885 ERR742413 2008

2009202419 ERR742414 2008

2008236096 ERR742415 2008

2009203420 ERR742471 2008

2008229069 ERR742507 2008

2009201605 ERR742508 2008

2008230676 ERR742509 2008

2008237194 ERR742512 2008

2009209487 ERR425441 2009

2009214138 ERR425443 2009

2009212746 ERR425444 2009

2009215824 ERR425446 2009

2009216796 ERR425447 2009

2009217657 ERR425448 2009

2009216885 ERR425449 2009

2009218340 ERR425450 2009

2009218344 ERR425451 2009

2010206278 ERR425452 2009

2010207747 ERR425453 2009

2010205337 ERR425454 2009

2010216300 ERR425456 2009

2010216301 ERR425457 2009

2010208449 ERR425459 2009

2010208464 ERR425460 2009

2010220066 ERR425461 2009

2010220077 ERR425462 2009

2010220093 ERR425463 2009

2009209134 ERR425464 2009

2009210878 ERR425465 2009

2009210894 ERR425466 2009

2009210924 ERR425467 2009

2009210929 ERR425468 2009

2009210939 ERR425469 2009

2009206436 ERR425470 2009

2009206439 ERR425471 2009

2009210956 ERR425472 2009

2009211969 ERR425473 2009

2009212151 ERR425474 2009

2009212157 ERR425475 2009

2009214547 ERR425476 2009

2009214084 ERR425477 2009

2009214110 ERR425478 2009

2009214119 ERR425479 2009

2009211097 ERR425481 2009

2009211109 ERR425482 2009

2009211113 ERR425483 2009

2009211298 ERR425484 2009

2009212737 ERR425485 2009

2009212749 ERR425486 2009

2009215659 ERR425489 2009

2009215693 ERR425490 2009

2009216681 ERR425491 2009

2009216712 ERR425492 2009

2009216731 ERR425493 2009

2009216784 ERR425494 2009

2009218018 ERR425495 2009

2009219216 ERR425497 2009

2009217627 ERR425498 2009

2009212654 ERR433603 2009

2009215893 ERR433605 2009

2009219217 ERR433606 2009

2009218376 ERR433609 2009

2010200701 ERR433611 2009

2010205250 ERR433612 2009

2010203158 ERR433613 2009

2010206305 ERR433614 2009

2010216160 ERR433616 2009

2010216216 ERR433617 2009

2010216329 ERR433618 2009

2010225202 ERR433619 2009

2010210038 ERR433621 2009

2010210040 ERR433622 2009

2010208344 ERR433623 2009

2010208391 ERR433624 2009

2010216336 ERR433625 2009

2010220141 ERR433626 2009

2009210864 ERR433627 2009

2009208549 ERR433628 2009

2009210882 ERR433629 2009

2009210885 ERR433630 2009

2009214095 ERR433632 2009

2009214096 ERR433633 2009

2009211089 ERR433635 2009

2009211239 ERR433636 2009

2009211240 ERR433637 2009

2009211245 ERR433638 2009

2009211299 ERR433639 2009

2009212727 ERR433640 2009

2009214002 ERR433641 2009

2009215630 ERR433647 2009

2009215639 ERR433648 2009

2009215663 ERR433649 2009

2009215833 ERR433650 2009

2009215894 ERR433651 2009

2009215906 ERR433653 2009

2009216744 ERR433654 2009

2009216752 ERR433655 2009

2009217629 ERR433657 2009

2010220088 ERR433697 2009

2009209148 ERR433698 2009

2009210875 ERR433699 2009

2009214113 ERR433700 2009

2009213970 ERR433701 2009

2010205421 ERR433702 2009

2010205400 ERR433704 2009

2010216214 ERR433705 2009

2010216340 ERR433706 2009

2010220100 ERR433707 2009

2010220104 ERR433708 2009

2009210937 ERR433710 2009

2009215139 ERR433711 2009

2009211232 ERR433713 2009

2009211242 ERR433714 2009

2009212753 ERR433716 2009

2009212770 ERR433717 2009

2009212800 ERR433718 2009

2009214056 ERR433720 2009

2009215629 ERR433721 2009

2009215690 ERR433722 2009

2009215743 ERR433723 2009

2009216680 ERR433724 2009

2009216788 ERR433725 2009

2009217663 ERR433901 2009

2009216816 ERR433902 2009

2009216718 ERR433903 2009

2009215718 ERR433905 2009

2009214077 ERR433907 2009

2009214041 ERR433911 2009

2009212842 ERR433912 2009

2009212813 ERR433913 2009

2009211285 ERR433914 2009

2009211248 ERR433915 2009

2009211142 ERR433916 2009

2009211102 ERR433917 2009

2009211095 ERR433918 2009

2009209868 ERR433919 2009

2009214185 ERR433920 2009

2009214180 ERR433921 2009

2009214152 ERR433923 2009

2009214100 ERR433925 2009

2009214092 ERR433926 2009

2009216160 ERR433927 2009

2009206423 ERR433929 2009

2009210907 ERR433930 2009

2010220061 ERR433931 2009

2010208390 ERR433933 2009

2009205598 ERR433934 2009

2010225245 ERR433935 2009

2010216328 ERR433936 2009

2010216179 ERR433937 2009

2010208312 ERR433938 2009

2010200609 ERR433939 2009

2009215863 ERR433940 2009

2009210231 ERR433941 2009

2009219991 ERR433943 2009

2009209140 ERR433944 2009

2009212862 ERR433945 2009

2009214170 ERR433966 2009

2009209968 ERR433967 2009

2009213982 ERR433968 2009

2009214049 ERR433969 2009

2009215635 ERR433970 2009

2009215714 ERR433971 2009

2009216739 ERR433972 2009

2009216746 ERR433973 2009

2009217662 ERR433974 2009

2009217704 ERR433975 2009

2009218384 ERR433976 2009

2009218395 ERR433977 2009

2010204328 ERR433978 2009

2010206332 ERR433979 2009

2010220285 ERR433980 2009

2010220311 ERR433981 2009

2010223357 ERR433983 2009

2010216155 ERR433984 2009

2010216259 ERR433985 2009

2010216272 ERR433986 2009

2010216283 ERR433987 2009

2010216320 ERR433988 2009

2010216335 ERR433989 2009

2010209958 ERR433991 2009

2010220049 ERR433992 2009

2010220067 ERR433993 2009

2009209280 ERR433995 2009

2009210224 ERR433996 2009

2009212155 ERR433997 2009

2009214410 ERR433998 2009

2009216993 ERR434000 2009

2009211258 ERR434001 2009

2009212648 ERR434002 2009

2009212708 ERR434003 2009

2009212769 ERR434004 2009

2009212859 ERR434006 2009

2009213981 ERR434007 2009

2009214014 ERR434009 2009

2009215660 ERR434015 2009

2009215701 ERR434016 2009

2009215771 ERR434017 2009

2009215888 ERR434018 2009

2009215804 ERR449188 2009

2009215889 ERR460198 2009

2009218325 ERR460199 2009

2009210908 ERR460200 2009

2009210936 ERR460201 2009

2010206720 ERR505730 2009

2010206726 ERR505731 2009

2010206729 ERR505732 2009

2010207743 ERR505733 2009

2010207769 ERR505734 2009

2010205298 ERR505735 2009

2010205338 ERR505736 2009

2010205342 ERR505737 2009

2010205347 ERR505738 2009

2010205398 ERR505740 2009

2010205404 ERR505741 2009

2010208283 ERR505742 2009

2010208292 ERR505743 2009

2010208307 ERR505744 2009

2010208327 ERR505745 2009

2010205423 ERR505746 2009

2010206270 ERR505747 2009

2010206275 ERR505748 2009

2010206276 ERR505749 2009

2010206713 ERR505750 2009

2010206716 ERR505751 2009

2010205418 ERR505753 2009

2010208330 ERR505754 2009

2010220270 ERR505755 2009

2010220276 ERR505756 2009

2010220340 ERR505758 2009

2010220349 ERR505759 2009

2010223233 ERR505760 2009

2010223244 ERR505761 2009

2010223245 ERR505762 2009

2010216166 ERR505763 2009

2010216187 ERR505764 2009

2010216188 ERR505765 2009

2010216219 ERR505766 2009

2010216221 ERR505767 2009

2010216229 ERR505768 2009

2010216231 ERR505769 2009

2010216248 ERR505770 2009

2010216251 ERR505771 2009

2010216304 ERR505772 2009

2010216334 ERR505773 2009

2009211518 ERR505774 2009

2010220051 ERR505776 2009

2009203268 ERR505777 2009

2009203513 ERR505778 2009

2009205599 ERR505779 2009

2009205600 ERR505780 2009

2009207275 ERR505781 2009

2009207277 ERR505782 2009

2010209966 ERR505786 2009

2010209973 ERR505788 2009

2010209984 ERR505789 2009

2010209989 ERR505790 2009

2010209998 ERR505791 2009

2010210051 ERR505792 2009

2010208343 ERR505793 2009

2010208351 ERR505794 2009

2010208354 ERR505795 2009

2010208358 ERR505796 2009

2010208374 ERR505797 2009

2010208412 ERR505799 2009

2010208429 ERR505800 2009

2010208437 ERR505801 2009

2010216075 ERR505803 2009

2010216081 ERR505804 2009

2010216092 ERR505805 2009

2010216114 ERR505806 2009

2010216128 ERR505807 2009

2010216137 ERR505808 2009

2010203401 ERR505843 2009

2010220110 ERR505846 2009

2010220116 ERR505847 2009

2010220126 ERR505848 2009

2010220131 ERR505849 2009

2009209482 ERR505855 2009

2009211070 ERR505874 2009

2010220245 ERR506035 2009

2010223246 ERR506042 2009

2010225299 ERR506056 2009

2010206274 ERR568758 2009

2010220298 ERR568759 2009

2010216163 ERR568760 2009

2009217665 ERR578019 2009

2009217675 ERR578022 2009

2009217687 ERR578023 2009

2009217709 ERR578025 2009

2009217714 ERR578026 2009

2009217716 ERR578027 2009

2009217744 ERR578031 2009

2009217747 ERR578032 2009

2010200695 ERR586316 2009

2010200704 ERR586317 2009

2009217751 ERR586384 2009

2009216881 ERR586388 2009

2009216946 ERR586390 2009

2009216956 ERR586391 2009

2009218212 ERR586392 2009

2009218219 ERR586393 2009

2009218231 ERR586394 2009

2009218297 ERR586395 2009

2009218333 ERR586396 2009

2009218397 ERR586398 2009

2009218402 ERR586399 2009

2009218403 ERR586400 2009

2009219960 ERR586401 2009

2009219987 ERR586402 2009

2009219995 ERR586403 2009

2010200663 ERR586404 2009

2010200669 ERR586405 2009

2010200683 ERR586406 2009

2010200696 ERR586407 2009

2010200690 ERR586409 2009

2010200703 ERR586411 2009

2009219983 ERR586413 2009

2010200029 ERR586414 2009

2010200031 ERR586415 2009

2010200604 ERR586416 2009

2010201630 ERR586417 2009

2010201909 ERR586419 2009

2010201916 ERR586420 2009

2010200718 ERR586421 2009

2010200746 ERR586422 2009

2010200750 ERR586423 2009

2010200769 ERR586424 2009

2010200772 ERR586425 2009

2010200804 ERR586426 2009

2010200810 ERR586428 2009

2010200814 ERR586429 2009

2010200835 ERR586431 2009

2010200836 ERR586432 2009

2010200837 ERR586433 2009

2010205248 ERR586435 2009

2010202227 ERR586437 2009

2010202234 ERR586439 2009

2010203149 ERR586440 2009

2010203153 ERR586441 2009

2010203159 ERR586443 2009

2010203160 ERR586444 2009

2010203475 ERR586445 2009

2010203480 ERR586446 2009

2010203932 ERR586447 2009

2010204335 ERR586448 2009

2010203356 ERR586449 2009

2010203382 ERR586450 2009

2010206293 ERR586451 2009

2010206325 ERR586452 2009

2010206334 ERR586453 2009

2010206338 ERR586454 2009

2010206353 ERR586455 2009

2010206361 ERR586456 2009

2010206372 ERR586457 2009

2010203406 ERR586458 2009

2010206717 ERR586459 2009

2009218327 ERR600092 2009

2010200823 ERR600110 2009

2010223273 ERR600213 2009

2009209135 ERR742607 2009

2009210887 ERR742608 2009

2009210808 ERR742609 2009

2009214073 ERR742610 2009

2009215598 ERR742611 2009

2009216705 ERR742612 2009

2009217643 ERR742613 2009

2009218296 ERR742614 2009

2010200686 ERR742615 2009

2010205351 ERR742616 2009

2010205399 ERR742617 2009

2010208284 ERR742618 2009

2010223248 ERR742619 2009

2009203695 ERR742620 2009

2009207274 ERR742621 2009

2010208432 ERR742622 2009

2010220052 ERR742623 2009

2009208020 ERR742624 2009

2009208544 ERR742625 2009

2009210923 ERR742626 2009

2009212612 ERR742627 2009

2009214140 ERR742628 2009

2009214145 ERR742629 2009

2009209907 ERR742630 2009

2009209974 ERR742631 2009

2009211127 ERR742632 2009

2009211133 ERR742633 2009

2009212807 ERR742634 2009

2009212823 ERR742635 2009

2009212860 ERR742636 2009

2009213994 ERR742637 2009

2009215898 ERR742638 2009

2009215911 ERR742639 2009

2009218157 ERR742640 2009

2009218419 ERR742641 2009

2009217666 ERR742642 2009

2010202229 ERR742663 2009

2013202610 ERR600112 2012

2013202641 ERR600113 2012

2013205806 ERR600114 2012

2012224830 ERR600115 2012

2012224843 ERR600116 2012

2012224849 ERR600117 2012

2012224855 ERR600118 2012

2012224867 ERR600119 2012

2012224883 ERR600120 2012

2012224895 ERR600122 2012

2013209063 ERR600123 2012

2013209049 ERR600124 2012

2013215778 ERR600125 2012

2013210858 ERR600126 2012

2013211438 ERR600127 2012

2013211698 ERR600128 2012

2013211701 ERR600129 2012

2013211747 ERR600130 2012

2013211724 ERR600131 2012

2013212244 ERR600132 2012

2013212457 ERR600133 2012

2013201122 ERR600134 2012

2013208476 ERR600135 2012

2013208478 ERR600136 2012

2013208489 ERR600137 2012

2013208490 ERR600138 2012

2013208509 ERR600139 2012

2013208527 ERR600140 2012

2013208535 ERR600141 2012

2013208537 ERR600142 2012

2013208538 ERR600143 2012

2013212823 ERR600144 2012

2013212827 ERR600145 2012

2012211937 ERR600146 2012

2012212663 ERR600147 2012

2012213285 ERR600148 2012

2012213946 ERR600149 2012

2012213994 ERR600150 2012

2012214035 ERR600151 2012

2012214417 ERR600152 2012

2012214435 ERR600153 2012

2012215849 ERR600154 2012

2012217274 ERR600155 2012

2012217544 ERR600156 2012

2012217851 ERR600157 2012

2012214442 ERR600158 2012

2012214475 ERR600159 2012

2012214919 ERR600160 2012

2012214924 ERR600161 2012

2012214932 ERR600162 2012

2012215792 ERR600163 2012

2012215830 ERR600164 2012

2012215805 ERR600165 2012

2012217982 ERR600166 2012

2013201331 ERR600167 2012

2013210881 ERR600168 2012

2013209135 ERR600169 2012

2013206378 ERR600170 2012

2013206373 ERR600171 2012

2013206212 ERR600172 2012

2013206201 ERR600173 2012

2012225592 ERR600174 2012

2012223303 ERR600175 2012

2013202261 ERR600176 2012

2013202258 ERR600177 2012

2013201628 ERR600178 2012

2013201224 ERR600180 2012

2013201223 ERR600181 2012

2012225750 ERR600182 2012

2012222470 ERR600183 2012

2012222457 ERR600184 2012

2012221540 ERR600185 2012

2012221546 ERR600186 2012

2012221404 ERR600187 2012

2012221324 ERR600188 2012

2012221315 ERR600189 2012

2012220659 ERR600190 2012

2012224918 ERR600191 2012

2012222918 ERR600192 2012

2012220613 ERR600193 2012

2012220587 ERR600194 2012

2012220570 ERR600195 2012

2012220439 ERR600196 2012

2012220440 ERR600197 2012

2012220414 ERR600198 2012

2012220354 ERR600199 2012

2012220353 ERR600200 2012

2012219980 ERR600201 2012

2012219972 ERR600202 2012

2012219021 ERR600203 2012

2012218663 ERR600204 2012

2012218039 ERR600206 2012

2012218036 ERR600207 2012

2012212051 ERR600208 2012

2012220098 ERR600209 2012

2012219141 ERR600211 2012

2012219138 ERR600212 2012

2012211517 ERR600214 2012

2012211938 ERR600215 2012

2012212660 ERR600216 2012

2012214450 ERR600218 2012

2012214497 ERR600220 2012

2012214490 ERR600221 2012

2012214930 ERR600222 2012

2012214921 ERR600223 2012

2012215019 ERR600224 2012

2012215791 ERR600225 2012

2012221164 ERR600228 2012

2012221165 ERR600229 2012

2012218235 ERR600231 2012

2012219977 ERR600232 2012

2012222280 ERR600235 2012

2012224338 ERR600236 2012

2012221583 ERR600238 2012

2012222487 ERR600239 2012

2012224315 ERR600240 2012

2013206355 ERR600242 2012

2013208587 ERR600243 2012

2013209090 ERR600244 2012

2013210876 ERR600245 2012

2013205808 ERR600247 2012

2013205805 ERR600248 2012

2013206298 ERR600250 2012

2013208721 ERR600252 2012

2013215781 ERR600253 2012

2013211404 ERR600254 2012

2013211429 ERR600256 2012

2013212446 ERR600258 2012

2013212860 ERR600259 2012

2013218411 SAMN03764924 2013

2014203093 SAMN03764925 2013

2013224378 SAMN03764926 2013

2013219299 SAMN03764927 2013

2013214882 SAMN03764928 2013

2013213837 SAMN03764929 2013

2013210938 SAMN03764930 2013

2014202035 SAMN03764931 2013

2013218180 SAMN03764932 2013

2013216690 SAMN03764933 2013

2013222813 SAMN03764934 2013

2014200696 SAMN03764935 2013

2014205022 SAMN03764936 2013

2013217256 SAMN03764937 2013

2013221606 SAMN03764938 2013

2014202193 SAMN03764939 2013

2014202199 SAMN03764940 2013

2014202558 SAMN03764941 2013

2014202538 SAMN03764942 2013

2014203075 SAMN03764943 2013

2013217514 SAMN03764944 2013

2013218080 SAMN03764945 2013

2014202550 SAMN03764946 2013

2013222074 SAMN03764947 2013

2014202576 SAMN03764948 2013

2013220175 SAMN03764949 2013

2013226310 SAMN03764950 2013

2013214888 SAMN03764951 2013

2013215520 SAMN03764952 2013

2013218767 SAMN03764953 2013

2014203848 SAMN03764954 2013

2013224419 SAMN03764955 2013

2014200801 SAMN03764956 2013

2014202556 SAMN03764957 2013

2014204010 SAMN03764958 2013

2013218240 SAMN03764959 2013

2013224358 SAMN03764960 2013

2014200864 SAMN03764961 2013

2013227862 SAMN03764962 2013

2013218738 SAMN03764963 2013

2013219514 SAMN03764964 2013

2013223642 SAMN03764965 2013

2014202041 SAMN03764966 2013

2014202885 SAMN03764967 2013

2014204525 SAMN03764968 2013

2013218751 SAMN03764969 2013

2013227863 SAMN03764970 2013

2014202157 SAMN03764971 2013

2014203083 SAMN03764972 2013

2014201466 SAMN03764973 2013

2013218334 SAMN03764974 2013

2013213344 SAMN03764975 2013

2014201545-c SAMN03764976 2013

2013216246 SAMN03764977 2013

2013213851 SAMN03764978 2013

2014201553-c SAMN03764980 2013

2014204584 SAMN03764981 2013

2014201763 SAMN03764982 2013

2014200702 SAMN03764983 2013

2013216695 SAMN03764984 2013

2013223640 SAMN03764985 2013

2014200763 SAMN03764986 2013

2014202166 SAMN03764987 2013

2013218176 SAMN03764988 2013

2013224801 SAMN03764989 2013

2013228088 SAMN03764990 2013

2014203816 SAMN03764991 2013

2013228259 SAMN03764992 2013

2013222842 SAMN03764993 2013

2014204536 SAMN03764994 2013

2013224917 SAMN03764995 2013

2014202503 SAMN03764996 2013

2013218341 SAMN03764997 2013

2014203994 SAMN03764998 2013

2013213852 SAMN03764999 2013

2013227583 SAMN03765000 2013

2014200597 SAMN03765001 2013

2014202234 SAMN03765002 2013

2013222043 SAMN03765003 2013

2014203084 SAMN03765004 2013

2014201854 SAMN03765005 2013

2013214880 SAMN03765006 2013

2013218407 SAMN03765007 2013

2014204541 SAMN03765008 2013

2013219473 SAMN03765009 2013

2014205695 SAMN03765010 2013

2014202525 SAMN03765011 2013

2014204514 SAMN03765012 2013

2013221281 SAMN03765013 2013

2014204552 SAMN03765014 2013

2014202569 SAMN03765015 2013

2014206986 SAMN03765016 2013

2014202149 SAMN03765017 2013

2013218174 SAMN03765018 2013

2013213977 SAMN03765019 2013

2014204530 SAMN03765020 2013

2014202560 SAMN03765021 2013

2013223617 SAMN03765022 2013

2013217306 SAMN03765023 2013

2013212937 SAMN03765024 2013

2013215817 SAMN03765025 2013

2014202158 SAMN03765026 2013

2013221965 SAMN03765027 2013

2014203868 SAMN03765028 2013

2013219315 SAMN03765029 2013

2014203865 SAMN03765030 2013

2014200662 SAMN03765031 2013

2014202506 SAMN03765032 2013

2013218394 SAMN03765033 2013

2013219484 SAMN03765034 2013

2013220191 SAMN03765035 2013

2014201418 SAMN03765036 2013

2014203844 SAMN03765037 2013

2013214876 SAMN03765038 2013

2013214076 SAMN03765039 2013

2013219866 SAMN03765040 2013

2013218100 SAMN03765041 2013

2013217515 SAMN03765042 2013

2014201481-c SAMN03765043 2013

2013221993 SAMN03765044 2013

2014202195 SAMN03765045 2013

2013221808 SAMN03765046 2013

2013217175 SAMN03765047 2013

2013217974 SAMN03765048 2013

2013219512 SAMN03765049 2013

2014204553 SAMN03765051 2013

2014200767 SAMN03765052 2013

2014201462 SAMN03765053 2013

2013218759 SAMN03765054 2013

2014201852 SAMN03765055 2013

2013218190 SAMN03765056 2013

2014203857 SAMN03765057 2013

2014205714 SAMN03765058 2013

2013222836 SAMN03765059 2013

2013211370 SAMN03765060 2013

2013214720 SAMN03765061 2013

2013214893 SAMN03765062 2013

2013222075 SAMN03765063 2013

2014205018 SAMN03765064 2013

2014204528 SAMN03765065 2013

2013224446 SAMN03765066 2013

2013222011 SAMN03765067 2013

2014204535 SAMN03765068 2013

2013216323 SAMN03765069 2013

2013226872 SAMN03765070 2013

2013224047 SAMN03765071 2013

2013218429 SAMN03765072 2013

2013219479 SAMN03765073 2013

2013219534 SAMN03765074 2013

2013226345 SAMN03765075 2013

2014202148 SAMN03765076 2013

2014202160 SAMN03765077 2013

2014202554 SAMN03765078 2013

2013218081 SAMN03765079 2013

2013213726 SAMN03765080 2013

2013219859 SAMN03765081 2013

2013222062 SAMN03765082 2013

2013221820 SAMN03765083 2013

2014201773 SAMN03765084 2013

2014201413 SAMN03765085 2013

2013226323 SAMN03765086 2013

2014203834 SAMN03765087 2013

2013218247 SAMN03765088 2013

2013226318 SAMN03765089 2013

2013224921 SAMN03765090 2013

2013218743 SAMN03765091 2013

2013216682 SAMN03765092 2013

2013213707 SAMN03765093 2013

2013222801 SAMN03765094 2013

2013213343 SAMN03765095 2013

2013221282 SAMN03765096 2013

2014201471 SAMN03765097 2013

2013224752 SAMN03765098 2013

2013226869 SAMN03765099 2013

2013228260 SAMN03765100 2013

2013219289 SAMN03765101 2013

2013214718 SAMN03765102 2013

2013221978 SAMN03765103 2013

2013222025 SAMN03765104 2013

2013218169 SAMN03765105 2013

2014202869 SAMN03765106 2013
