# Supplementary material for: Strain features and distributions in pneumococci from children with invasive disease before and after 13-valent conjugate vaccine implementation in the USA
Source: Clin Microbiol Infect. 2016 Jan;22(1):60.e9–60.e29. doi: 10.1016/j.cmi.2015.08.027 (PMC4721534; doi:10.1016/j.cmi.2015.08.027)
Supplement: Doc. S1 — Bioinformatic methods. [file mmc7.docx]

Supplement 1: Bioinformatic Methods.

An in-house wrapper script called JanOw_Spn was created to obtain MLST (<http://pubmlst.org/spneumoniae/> ), serotype/pili and drug resistance information from Illumina paired-end whole genome sequence.  The main program used to detect sequence types and document the presence of genes related to both serotype classification and antibiotic drug resistance was the short read typing tool SRST2 [1]. Some serotype, drug resistance predictions, and PBP transpeptidase typing required the extraction of specific sequence for further analysis.  For low-divergent genomic targets where a reference was available, this sequence was generated using a pipeline that fed the sorted bam alignment file from SRST2 to the FreeBayes[3] variant caller and then the vcf-consensus program provided by VCFtools [4].

Although read mapping worked well in detecting most of the loci used in our typing strategy, a different approach was needed when extracting potentially polymorphic mosaic regions.  These regions include FolA-1 (a marker for trimethoprim and cotrimoxazole resistance), RPLD-1 (a marker for macrolide+lincosamide+quinupristin/dalfopristin resistance), as well as the three PBP genes associated with beta-lactam resistance.  This alternative strategy employed a script called loTrac_gene that wrapped around a pipeline built upon the adapter trimming tool Cutadapt v1.6[ref5], the VelvetOptimiser v2.2.5[ref6] assembler, the Prodigal v2.60[ref7] gene predictor and BLAST v2.2.29[ref8].

This in-house tool pre-processed the paired-end reads with Cutadapt v1.6 by (1) calling the ‘-q 20’ option to remove low quality bases from the end of the read, (2) trimming the Illumina TruSeq universal and index adapters with the ‘-b’ argument and (3) filtering out reads less than 50 bases with the ‘--minimum-length’ option.  De novo assembly was carried out with Velvet v1.2.10 as optimized by the VelvetOptimiser v2.2.5 wrapper.  Scaffolding was turned off and the Perl script VelvetK (<http://bioinformatics.net.au/software.velvetk.shtml>) was used to estimate the best kmer length given a 2.1M size genome.  Gene predictions were performed by Prodigal v2.60 with the -c option called and three output files specified using the ‘-a’, ‘-d’ and ‘-f gff’ options.  A BLAST database was generated from the predicted gene nucleotide sequence and a representative query sequence passed to loTrac_gene as an argument was blasted against the database using blastn with ‘-word_size’ set to 7 and output format designated by ‘-outfmt 6’.  A best blast hit was determined by sorting the blast output by bit score, percent identity and alignment length and extracting alignment information from the first row.  A match was called if the query aligned against the best blast hit with at least 70% sequence identity and 25% coverage.  By default, loTrac_gene output the nucleotide and amino acid sequence of the predicted gene that matched the query sequence.  However, if the ‘-p’ flag was given, then the script would also extract the section of the target sequence that corresponds to just the query fragment using the bedtools v2.17[ref 9] getfasta command.  If the best blast hit did not include the entire query fragment, then the code would calculate the expected start/end coordinates of the complete fragment and would attempt to extract the full fragment from the predicted gene sequence.

The current version of this wrapper script was written in the bash programming language and requires 12 dependencies.  Although not designed as an end-user application, this code can be accessed at the following GitHub account: https://github.com/BenJamesMetcalf/JanOw_Spn.

The following provides further explanation of how each tool was used for each typing method as well as what options were selected.

MLST:

The short read typing tool SRST2 was used to detect sequence types using *Streptococcus* *pneumoniae* MLST sequences and definitions obtained from the PubMLST database (<http://pubmlst.org/data/>).  A slight modification was made to the SRST2 code to allow anomalous reads when generating the mpileup file (-A option).

Serotype and Pili:

Employing the modified SRST2 script described above, serotyping and pili detection was carried out using the gene database outlined in sTable 1 as a reference.  Additionally, SRST2 was called with the ‘--min_coverage’ option set to 99 and the ‘--max_divergence’ set to 5.  When a typing target required the extraction of loci sequence, the script generated a consensus sequence by passing the sorted bam file from SRST2 to the FreeBayes variant caller [with --min-base-quality set to 20] and using the VCFtools program vcf-consensus to apply those variants to the reference sequence. The actual serotype calling was handled by the script seroType_pred using the fullgenes output table from SRST2 and the serotype target consensus sequence.

PBP transpeptidase region typing:

Because many of the PBP alleles associated with resistance contain mosaic regions created by recombination events, characterizing them could only be done reliably with the loTrac_gene program.  A query file containing representative fragments of the three PBP genes was passed to loTrac_gene along with the samples’ paired-end sequence files.  The ‘-p’ flag was called and the three extracted PBP amino acid sequence fragments were then compared against their respective databases using a script called bLactam-PBP_Typer.  This program worked similarly to loTrac_gene with each PBP fragment being blasted against its database using the blastp program [employing the ‘-outfmt 6’ option] and the best blast hit found by ranking the results by bit score, percent identity and alignment length.  If a perfect match was found [percent identity = 100 and alignment length = query length] then that allele number would be assigned to the sequence.  A new allele would be assigned ‘NA’ and PBP sequences that couldn’t be extracted because of assembly problems were designated as ‘NF’.

Non β lactam Drug Resistance:

Non β lactam antibiotic resistance typing was performed using a combination of the tools discussed above.  Most of the genomic targets described in sTable 2 could be typed using a reference-based approach based upon the modified SRST2 script.  For loci diverging very little from the reference sequence, SRST2 was called with the ‘--min_coverage’ option set to 99 and the ‘--max_divergence’ set to 5.  For more variable regions (like those encompassed by the FolP query) or those without any observed precedence in pneumococci like the van operon targets, the ‘--max_divergence’ threshold was lowered to 20.  Similar to the serotype and pili pipeline, target consensus sequence was generated by calling variants with FreeBayes [with --min-base-quality set to 20] and using the VCFtools program vcf-consensus to apply those variants to the reference sequence. For the case of FolP, we used the vcf file directly to determine if an indel was present within the target region.

Two genomic markers for non-beta lactam resistance – FolA-1 (Trimethoprim/cotrimoxazole) and RPLD-1 (macrolide+lincosamide+quinupristin/dalfopristin) - had alleles that contained mosaic regions that prevented them from being reliably characterized using SRST2.  These loci were detected with the loTrac_gene script described above.

The calling of non β lactam resistance was performed by the script ‘miscRes_Typer’ using the fullgenes output table from SRST2, the non-beta lactam target consensus sequence and the FolA and RPLD-1 genomic loci sequence extracted by loTrac_gene.

References:

1. M. Inouye *et al.*, SRST2: Rapid genomic surveillance for public health and hospital microbiology labs. *Genome Med* **6**, 90 (2014).

2. E. Garrison, G. Marth, Haplotype-based variant detection from short-read sequencing. *arXiv e-prints* **1207**, 3907 (2012).

3. P. Danecek *et al.*, The variant call format and VCFtools. *Bioinformatics* **27**, 2156-2158 (2011).

4. M. Martin, Cutadapt removes adapter sequences from high-throughput sequencing reads. *EMBnet* **17**, 10-12 (2011).

5. D. R. Zerbino, E. Birney, Velvet: algorithms for de novo short read assembly using de Bruijn graphs. *Genome Res* **18**, 821-829 (2008).

6. D. Hyatt *et al.*, Prodigal: prokaryotic gene recognition and translation initiation site identification. *BMC Bioinformatics* **11**, 119 (2010).

7. S. F. Altschul, W. Gish, W. Miller, E. W. Myers, D. J. Lipman, Basic local alignment search tool. *J Mol Biol* **215**, 403-410 (1990).

8. A. R. Quinlan, I. M. Hall, BEDTools: a flexible suite of utilities for comparing genomic features. *Bioinformatics* **26**, 841-842 (2010).


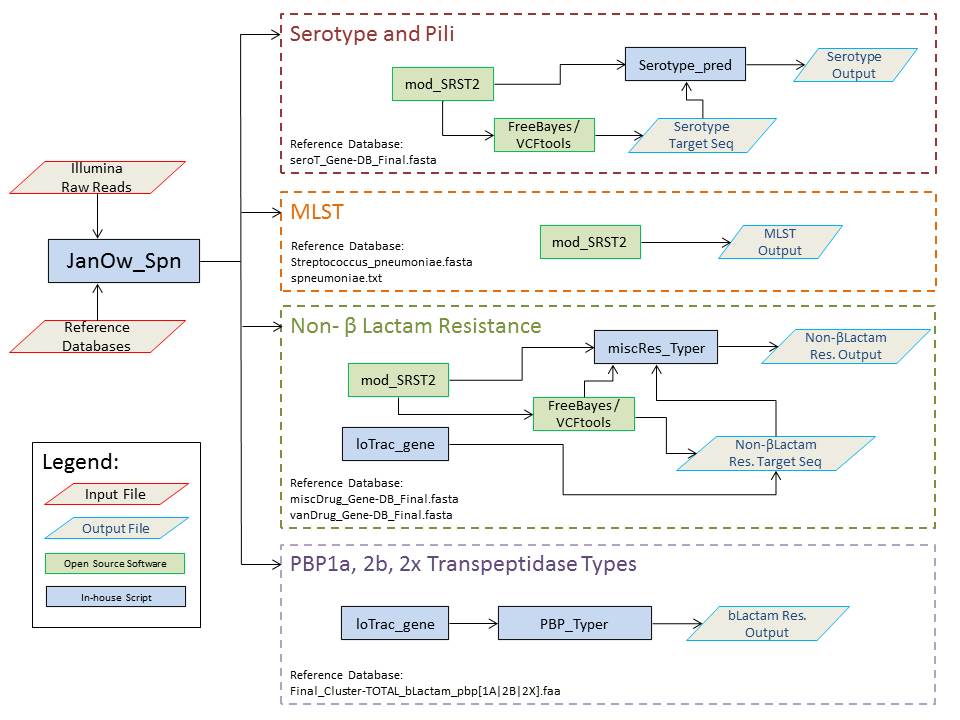
sFig.1.

This flow diagram represents the overall structure of the JanOw_Spn wrapper script which handles the code responsible for obtaining MLST, serotype, pili and drug resistance information from Illumina paired-end whole genome sequence.  The absolute path locations of both the Illumina sequence directory and the reference database directory are given as inputs to the JanOw_Spn wrapper.  After validating those arguments, the code channels the appropriate input files to each of the four typing pipelines: (1) Serotype and Pili, (2) MLST, (3) Non β Lactam Resistance and (4) PBP 1a, 2b, 2x Transpeptidase Typing.  These individual pipelines characterize Streptococcus pneumoniae strains using a combination of both in-house scripts and open source bioinformatics tools.
